# A Concise Process to Lenacapavir Sodium: Demonstration of One-Pot Sequential Heck/Suzuki-Miyaura Couplings in Lenacapavir API Synthesis

**DOI:** 10.1021/acs.oprd.5c00443

**Published:** 2026-03-03

**Authors:** Daryl Guthrie, Anand H. Shinde, Ramesh Vediyappan, Aline Nunes De Souza, Rama Krishna Sayini, John M. Saathoff, Nagaraju Sakkani, Naeem Asad, Samuel R. Hochstetler, Barrack Stubbs, Justina M. Burns, B. Frank Gupton, Ryan Littich, Limei Jin

**Affiliations:** Medicines for All Institute, 6889 Virginia Commonwealth University, Richmond, Virginia 23284-3068, United States

**Keywords:** lenacapavir, one-pot Heck/Suzuki-Miyaura sequence, API synthesis, process development, Pd sequestration

## Abstract

This article presents a new three-step process for lenacapavir sodium, a first-in-class HIV capsid inhibitor developed by Gilead Sciences. Lenacapavir has received FDA approval for both the treatment of multidrug-resistant HIV (Sunlenca^Ⓡ^, 2022) and PrEP (Yeztugo^Ⓡ^, 2025). Previous medicinal chemistry synthetic routes relied on separate Pd-catalyzed couplings and involved atropisomeric intermediates, resulting in low efficiency. Gilead’s 2024 four-step process improved scalability but retained discrete Pd-catalyzed steps and early stage incorporation of the costly chiral pyrazole carboxylic acid (**Frag C**). Our streamlined approach begins with a chiral bromopyridine (**Frag A**) and features telescoped Heck/Suzuki-Miyaura coupling and late-stage amidation with **Frag C**. This marks the first reported one-pot Heck/Suzuki-Miyaura sequence in the synthesis of lenacapavir, minimizing atropisomer handling and simplifying Pd sequestration. The process affords lenacapavir API in up to 55% overall yield, with >99.9 wt % purity, and Pd content below 4.2 ppm.

## Graphical abstract

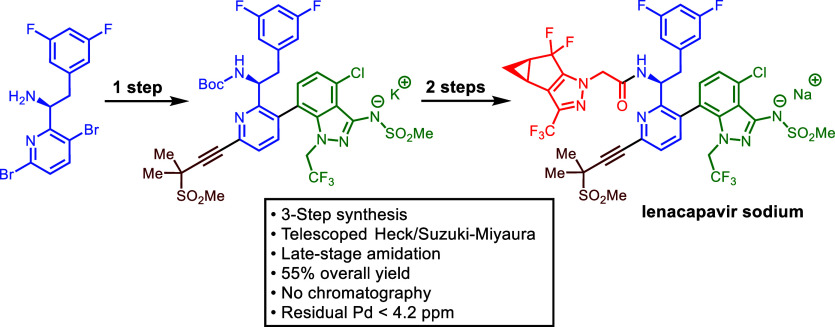

## Introduction

The Human Immunodeficiency Virus (HIV) continues to pose one of the most urgent and enduring challenges to global public health. It is estimated that more than 600,000 people die from HIV-related illnesses each year, with over 40 million deaths recorded since the beginning of the epidemic.^1^ Currently, approximately 40 million individuals are living with HIV worldwide, including 1.5 million children; more than 1 million new infections occur annually.^2,3^ Among the most promising therapies for HIV treatment is lenacapavir, a first-in-class antiviral that targets the HIV capsid protein.^4–7^ This novel approach disrupts multiple stages of the viral life cycle. Lenacapavir’s long-acting nature and availability in both oral and injectable forms have positioned it as a first-line treatment for HIV infection. In 2022, the U.S. Food and Drug Administration (FDA) approved lenacapavir for the treatment of multidrug-resistant HIV.^8^ Notably, the drug also demonstrated strong efficacy in preventing HIV. Marketed under the trade name *Yeztugo* ^Ⓡ^, lenacapavir was approved by the FDA in 2025 for use as a pre-exposure prophylaxis (PrEP) against HIV.^9^

Despite its clinical success, lenacapavir has faced high cost issues.^10–12^ According to 2024 data, the cost of goods (COG) for this active pharmaceutical ingredient (API) stands at $64,480 per kilogram.^11^ To make lenacapavir more accessible, it requires reducing the generic manufacturing cost of the API. An innovative synthesis and process provide the key to achieving the goals.

Lenacapavir was first reported by Gilead Sciences in a family of patents and publications in 2018–2023.^13–17^ In 2020, Gilead Sciences disclosed the medicinal chemistry route to lenacapavir free acid.^15,17^ The synthesis commenced from the N-Boc-protected chiral bis-bromopyridine core and proceeded through a series of transformations, including Sonogashira coupling (with a cocatalyst Cu­(I)), Suzuki-Miyaura cross-coupling, and bis-methanesulfonylation. This was followed by Boc deprotection, amidation, and selective monodemesylation to yield the API. This route involved two separate Pd-catalyzed coupling reactions and required careful management of intermediates exhibiting atropisomerism (atropisomers about the coupled **Frag A** and **Frag B** bond). To decrease the analytical and processing complexity associated with atropisomeric compounds and enhance synthetic efficiency, Gilead revealed a scale-up process for lenacapavir sodium in 2024,^18^ which is characterized by the convergence of three advanced intermediates (or **Frag A**, **B**, **C**) and a propargyl sulfone (DMPS or **Frag D**, in Figure 1). The central fragment of lenacapavir is **Frag A** (chiral amine), which is joined to **Frag D** through Heck alkynylation (without a cocatalyst Cu­(I)),^19^ to **Frag C** (chiral carboxylic acid) using a T3P-promoted amide coupling, and then to **Frag B** (indazole boronic ester) using the Suzuki-Miyaura reaction (Scheme 1a).

**Figure 1:**
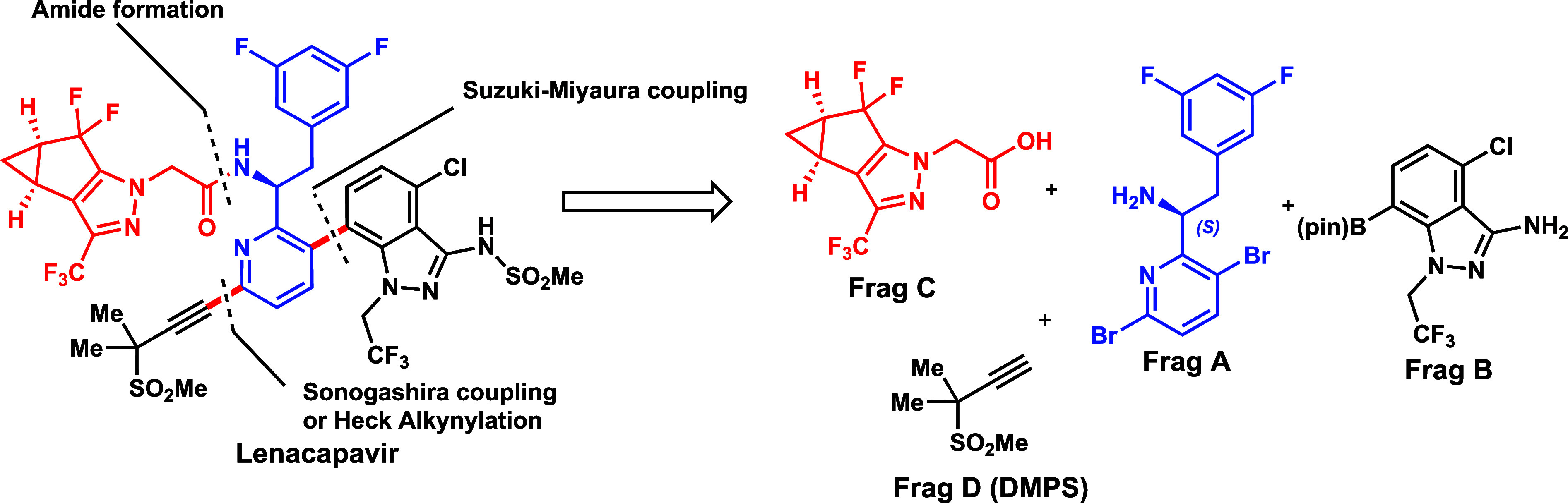
Retrosynthetic disconnections for lenacapavir and its constituents for chemical synthesis.

**Scheme 1:**
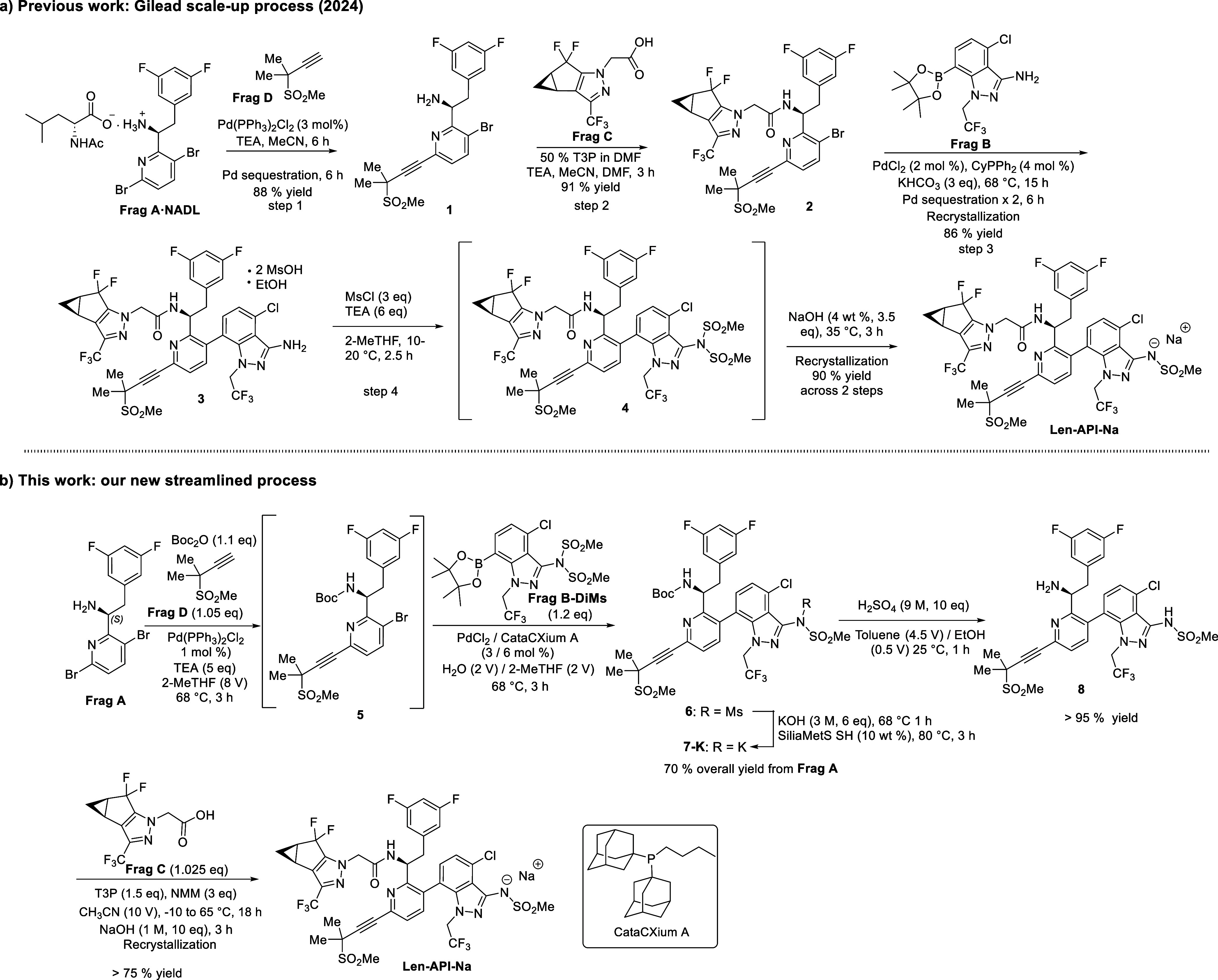
a) Gilead’s Scale-Up Process for the Synthesis of Lenacapavir (Reproduced/Adapted with Permission from Ref.^18^ Copyright 2024 ACS.); b) Our New Process for the Synthesis of Lenacapavir

Gilead’s groundbreaking synthetic approach enabled the successful construction of the complex lenacapavir API molecule, representing a significant advancement in HIV drug development. However, this approach faces three major issues that challenge its cost-effectiveness. First, the early stage introduction of **Frag C**, itself a key cost driver, increases the raw material costs. Second, the process utilizes two discrete palladium-catalyzed couplings (Heck alkynylation; Suzuki-Miyaura), which add to raw material and operational costs (e.g., late-stage palladium sequestration to meet pharmaceutical purity standards). Third, the workflow is hindered by demanding multiple purification processes, reducing throughput and increasing production complexity. To address opportunities for complexity and cost reduction, the development of a new synthesis pathway is essential.

With ongoing support from the Gates Foundation, M4ALL devised a cost-effective synthesis of lenacapavir API sodium salt (**Len-API-Na**), as illustrated in Scheme 1 (b).^20^ Our new process features a streamlined one-pot Heck/Suzuki-Miyaura sequence and late-stage **Frag C** ^21^ incorporation. The synthesis begins with the coupling of **Frag A** ^22,23^ and **Frag D** in the presence of TEA, Boc_2_O and a catalytic amount of PdCl_2_(PPh_3_)_2_. The resulting Heck product undergoes a telescoped Suzuki-Miyaura coupling with **Frag B-DiMs** (derivatized from **Frag B** ^24,25^ with one step), followed by KOH treatment to yield the key intermediate **7-K**. Subsequent Boc-deprotection and T3P-mediated amidation with **Frag C** produce **Len-API-Na**, finalized through recrystallization. Compared to the known process, this new process poses several advantages as summarized below: 1) The one-pot Heck/Suzuki-Miyaura sequence lowers total Pd consumption and reduces intermediate purification steps; 2) streamlined Pd removal and fewer isolations enhance space-time yield and reduce effluent; 3) the process reduces steps for handling of atropisomeric intermediates; 4) late-stage amidation with **Frag C** likely lowers overall synthesis costs. Herein, we present a detailed R&D process development of **Len-API-Na**.

## Results and Discussion

Our initial efforts focused on developing Pd-free couplings and amine-protection-free approaches,^26–28^ proved instrumental to developing the topical process. Attempts to install the alkyne moiety via the nucleophilic aromatic substitution (S_N_Ar) of **Frag D-Na** (generated from **Frag D** and NaH) with **Frag A** were unsuccessful. Similarly, CuI-catalyzed alkynylation trials resulted in the recovery of the starting materials. While the unprotected amine in **Frag A** was well tolerated in Heck alkynylation, subsequent Suzuki-Miyaura coupling with either **Frag B-DiMs** or **Frag B** failed to incorporate the indazole fragment. One key takeaway from our study was that a protected amine in **Frag A** was necessary for the Pd-catalyzed Suzuki-Miyaura reaction. As shown in Scheme 2, Suzuki-Miyaura coupling between Boc-protected amine **5** and either **Frag B** or **Frag B-DiMs** proceeded smoothly, yielding the desired coupling products. Notably, the reaction with **Frag B-DiMs** resulted in in situ demesylation, eliminating the need for a separate demesylation step. Although the overall yield was moderate, these early findings informed our development of a more process-friendly, one-pot Pd-catalyzed coupling strategy.

**Scheme 2:**
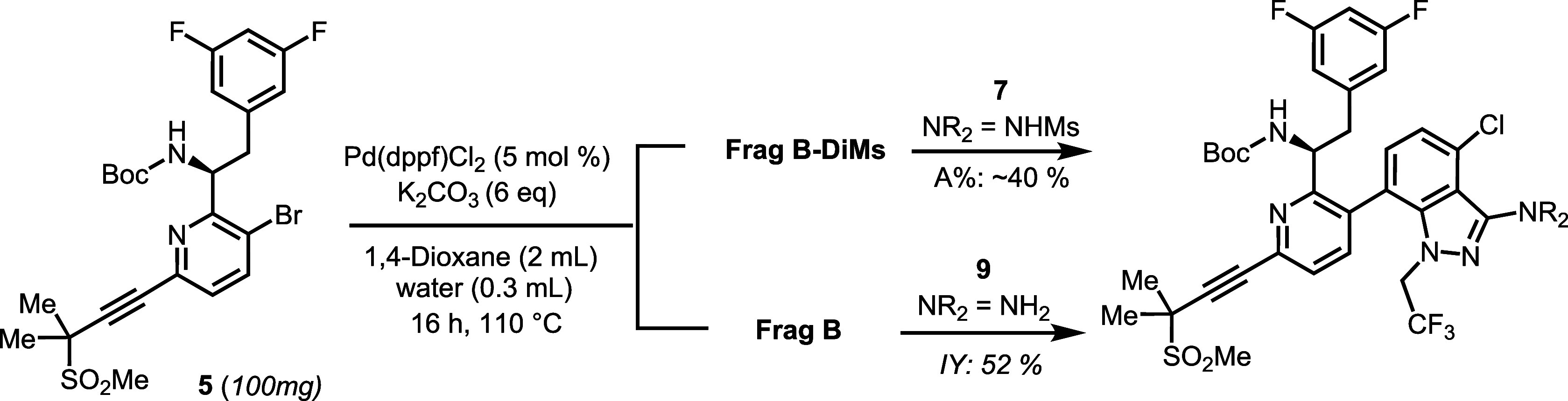
Evaluation of **5** (Boc-Protected Amine) in Suzuki-Miyaura Coupling with **Frag B** and **Frag B-DiMs**

An initial demonstration of the in situ Boc protection combined with the Heck reaction was conducted to assess the feasibility of the one-pot approach.^29–31^ The reaction of **Frag A** with **Frag D**, TEA, and Boc_2_O, in the presence of 0.6 mol % of PdCl_2_(PPh_3_)_2_, yielded the desired Boc-protected Heck coupling product **5** in approximately 95 A % (275 nm) with approximately 5 A % (275 nm) of bisalkynylated products being detected (Scheme S1 in Supporting Information). Notably, the Heck reaction proceeded more slowly in the absence of Boc_2_O, highlighting the beneficial role of in situ Boc protection in promoting reaction efficiency.

To assess the feasibility and practicality of a telescoped one-pot Heck/Suzuki-Miyaura sequence to **7**, an initial reaction with (*rac*)-**10** was conducted using Pd­(PPh_3_)_2_Cl_2_ (5 mol %) as a single catalyst (Table 1). This sequential transformation afforded **7** in approximately 30 A %, with 14 A % (275 nm) of the intermediate **5** remaining. Further catalyst screening showed that Pd_2_(dba)_3_/PPh_3_ delivered the highest yield of **7**, albeit with quantities of **5** remaining unconverted, as summarized in Table 1. Variations in base and solvent did not improve yields of **7** and adjusting the reaction temperature from room temperature (rt) to 100 °C failed to enhance the performance (data not shown). Notably, under Suzuki-Miyaura conditions, one of the methylsulfonamide groups was cleaved during the reaction, yielding the desired product without the need for an additional hydrolysis step.

**Table 1:**
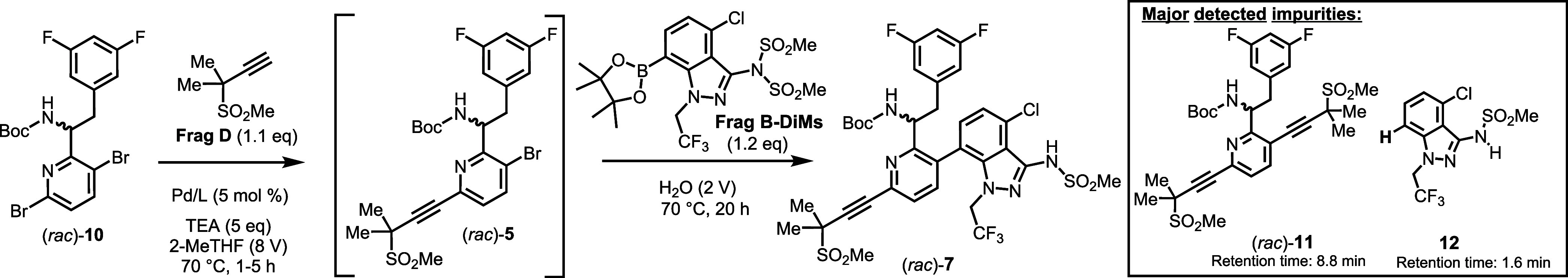
Single-Dosed Pd/L Catalyst Screen for the One-Pot Heck/Suzuki-Miyaura Reaction

		In-process analysis (A %, 275 nm)^b^				
		Step 1 (alkynylation)			Step 2 (Suzuki-Miyaura)	
#^a^	Pd/L (5 mol %)	(*rac*)-**10**	(*rac*)-**5**	(*rac*)-**11**	(*rac*)-7^c^	(*rac*)-**5**
1	Pd(PPh_3_)_2_Cl_2_	4	90	1	30	14
2	Pd(PPh_3_)_4_	8	79	-	20	25
3	Pd(dppf)Cl_2_	11	87	2	15	49
4	PdCl_2_ /PPh_3_	3.2	81	0.6	34	15
5	PdCl_2_ /TFP	2	91	3	30	24
6	Pd_2_(dba)_3_/PPh_3_	0.1	72	5	50	15
7	Pd_2_(dba)_3_/TFP	ND	77	3	39	19

a All reactions were performed on 100 mg scale of (*rac*)-**10**.

b A % refers to UV area % measured at 275 nm by HPLC.

c Two atropisomers (minor/major (A %) ∼ 1/5) were observed.

While this study demonstrated that a single-dose Pd catalyst strategy could, in principle, effect the telescoped Heck/Suzuki-Miyaura sequence, it was ultimately limited by its Suzuki-Miyaura coupling performance.

Modest conversion to (*rac*)-**7** and the persistent presence of intermediate (*rac*)-**5** highlighted the need for improvement in the activity of the catalyst in the Suzuki-Miyaura reaction. Building on the benefits observed from the alkynylation-*co*-Boc protection step using Pd­(PPh_3_)_2_Cl_2_ (1 mol %) as a catalyst, a double-dose catalyst approach was pursued. This involved introducing a second Pd catalyst dose specifically to enhance the Suzuki-Miyaura coupling efficiency.

A variety of preformed Pd-catalysts and Pd/L combinations were investigated as a second dose of the catalyst in the Suzuki-Miyaura reaction. As summarized in Table 2, this dual-catalyst strategy addressed the limitations of the single-dosed system, offering a more robust and higher-yielding approach for the telescoped Heck/Suzuki-Miyaura sequence. Catalysts well-suited for sterically hindered Suzuki-Miyaura reactions, such as PdCl_2_/CataCXium A^32^ and PdCl_2_/BIDIME,^33^ performed well in this sequence. Triethylamine (TEA) was the base for the Heck/Suzuki-Miyaura sequence. Notably, PdCl_2_/CataCXium A delivered 73 A % (275 nm) of **7**. Evaluation of various Pd sources showed PdCl_2_ was the optimal choice for the transformation (Table 2, Entries 7 and 15–18). Notably, across all double-dosed sequences, 1 mol % of Pd­(PPh_3_)_2_Cl_2_ was used as the catalyst in the Heck reaction, consistently yielding **5** in 85–95 A % (275 nm). Across all catalytic systems tested, a notable amount of **6** was observed, indicating the need for an optimized base to enable efficient demesylation.

**Table 2:**
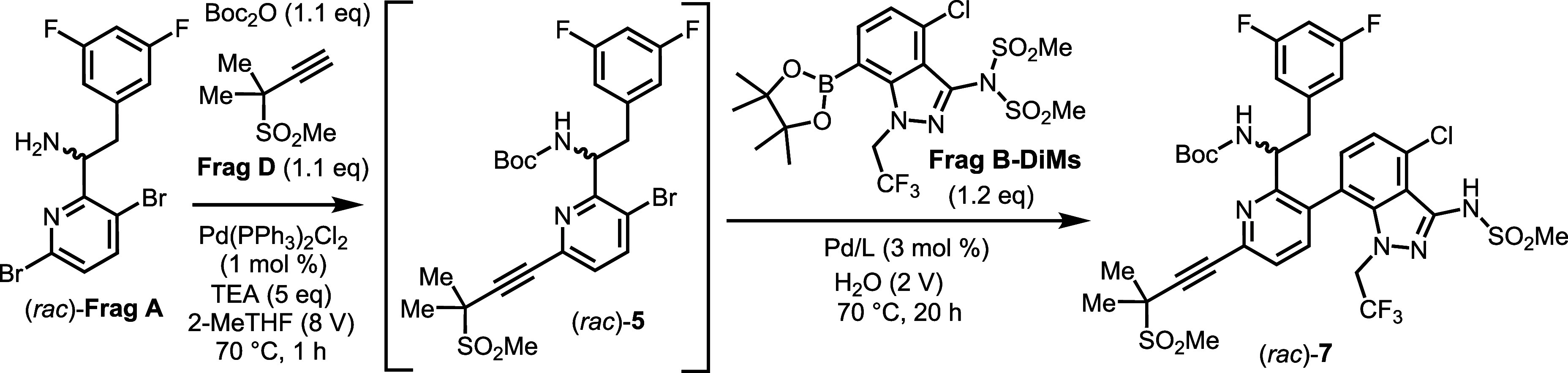
Double-Dosed Pd/L Catalyst Screen for One-Pot Heck/Suzuki-Miyaura Reaction

		In-process analysis (A %, 275 nm)^b^	
#^a^	Pd/L	(*rac*)-7^c^	(*rac*)-**5**
1	PdCl_2_ /CyPPh_2_	59	7
2	PdCl_2_ /XPhos	56	15
3	PdCl_2_ /SPhos	55	13
4	PdCl_2_ /RuPhos	55	15
5	PdCl_2_ /tBuBrettPhos	53	17
6	PdCl_2_/dppf	60	ND
7	PdCl_2_ /CataCXium A	73	<5
8	PdCl_2_ /BIDIME	62	<5
9	Pd(PPh_3_)_2_Cl_2_	52	<5^d^
10	Pd(PPh_3_)_4_	54	<8^d^
11	Pd(dppf)Cl_2_	54	<4^d^
12	XPhos Pd G2	50	<14^d^
13	XPhos Pd G3	51	<16^d^
14	PdCl_2_ /TFP	49	<13^d^
15	Pd(OAc)_2_ /CataCXium A	52	<16^d^
16	Pd_2_(dba)_3_ /CataCXium A	63	ND
17	Pd(allyl)Cl/CataCXium A	51	ND
18	Pd(dppf)Cl_2_ /CataCXium A	71	≤3^d^

a All reactions were performed on 100 mg scale of (*rac*)-**Frag A**; In situ Boc-protection and Heck coupling afforded (*rac*)-**5** in >90A % in all screens with 1 mol % of Pd­(PPh_3_)_2_Cl_2_ as catalyst.

b A % refers to UV area % measured at 275 nm by HPLC.

c Two atropisomers (minor/major (A %) ∼ 1/5) were observed.

d **5** coeluted with **6** isomer; ND = nondetect.

Base screening for demesylation revealed that KOH achieved excellent conversion in the demesylation step, with residual **6** remaining below 1 A %. Additionally, the concomitant formation of potassium salts aided precipitation during workup. Additionally, further optimization of equivalents of reagents was achieved by a one-factor-at-a-time (OFAT) strategy.

It was found that 1.05–1.06 equiv of **Frag D** were optimal in the Heck reaction to achieve full conversion of **10**, yielding approximately 95 A % (275 nm) **5** along with a manageable amount of side product **11**. Excess **Frag D** reacted with the meta-bromo position, leading to undesired bisalkynylation, whereas insufficient **Frag D** resulted in incomplete conversion of **10**, which would subsequently undergo bis-Suzuki-Miyaura reaction with **Frag B-DiMs** and complicating purification during the final API isolation.

In parallel, efforts on further optimization of the **Frag B-DiMs** quantity and catalyst loading in Suzuki-Miyaura reaction identified ideal scale-up conditions: 1.2 equiv of **Frag B-DiMs**, 3 mol % of PdCl_2_ and 6 mol % of CataCXium A.

With optimized reaction conditions established, efforts shifted toward developing an efficient purification process, such as liquid–liquid extraction (LLE) workup method for the removal of impurities, Pd residues, and product precipitation. To evaluate the solubility and partitioning behavior of **7**, its p*K*_a_ was measured in MTBE via acid–base titration^34^ and determined to be approximately 10.5. Partitioning between MTBE and water across varying pH levels was assessed by HPLC. **7** exhibited high solubility in MTBE at pH values below 10 (0.5 units below its p*K*_a_). Conversely, at pH values above 12 (1.5 units above its p*K*_a_), the alkaline salt form of **7-K** showed excellent solubility in the aqueous layer. These findings suggested that adjusting the reaction mixture to pH > 12.5 might enable effective removal of organic impurities by solvent wash. Various organic solvents (toluene, EtOAc, iPrOAc, 2-MeTHF, MEK, MIBK, etc.) were screened to purge organic impurities from the basic aqueous solution. Among the screened solvents, MTBE was optimal for purging impurities such as **11** and **5** (both of which lack free methylsulfonamide (−NHMs) groups). MIBK showed excellent solubility for **7-K** across the entire pH range. Although ineffective at purging nonpolar impurities, MIBK exhibited excellent phase separation and proved to be a highly effective solvent for extracting the desired product from the aqueous layer and purging polar impurity **12**. Initial application of MTBE in the LLE workup of alkaline **7-K**, to purge impurities, resulted in significant product loss. Residual 2-MeTHF from the telescoped coupling reaction was identified as the primary cause. To address this, azeotropic distillation with water was employed to remove 2-MeTHF from the system. Its removal effectively minimized product loss in subsequent MTBE washes. Three MTBE washes were sufficient to purge organic impurities, resulting in a crude of ∼95 A % (HPLC, 275 nm). Crude **7-K** was then retrieved from the aqueous layer via one MIBK extraction. To precipitate **7-K** from the MIBK solution, 5 volumes of MIBK and 10 volumes of heptane were identified as the optimal precipitation conditions.

Based on the established LLE workup and precipitation conditions, **7-K** was isolated as a light-yellow solid in 62–70% yield, with approximately 90 wt % purity (>97 A %, 275 nm).

To meet the Pd content specifications in the final API,^35,36^ a range of Pd scavengers was screened to reduce residual Pd in **7-K**. As summarized in (Table S1 SI), 20 mol % NAC and PIX treatments reduced Pd content from 395 to 61 ppm and 89 ppm, respectively.^37,38^ While initially promising, further reduction of Pd could not be achieved. In contrast, SiliaMetS scavengers showed superior performance.^39,40^ Most variants exhibited strong Pd removal capabilities, with 100 wt % SiliaMetS SH reducing the level of Pd to 13 ppm. Subsequent studies revealed temperature as a critical factor impacting scavenging efficiency (Table 3). At 50 °C, lower loadings (75 or 25 wt %) only reduced Pd to ∼100 ppm. However, at 80 °C, significant improvement was observed: both 5 and 10 wt % loadings reduced Pd to below 10 ppm after 20 h. Notably, a shorter 3 h treatment at this temperature was also sufficient to achieve Pd levels below 10 ppm. Importantly, the Pd removal process did not introduce additional impurities, and the purity profile remained unchanged or slightly improved.

**Table 3:**
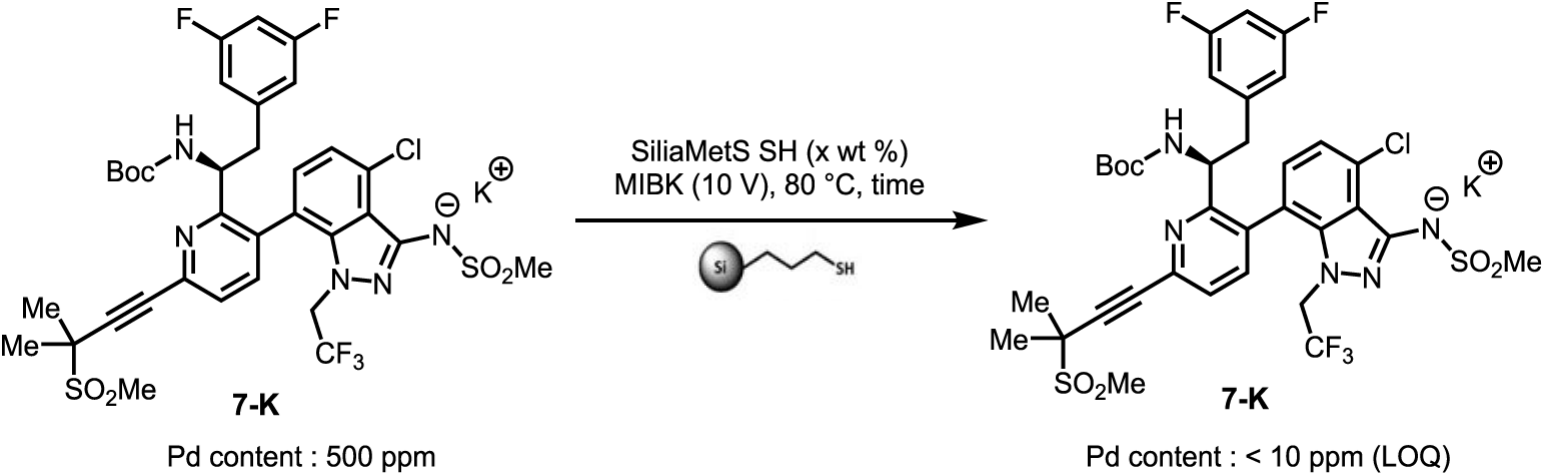
Efficiency Improvement of Pd Removal in **7-K**

#^a^	SiliaMetS SH (wt %)	Temp (°C)	Time (h)	Pd (ppm)	Recovery yield (%)
1^b^	-		-	500	-
2	75	50	3	129	95
3	25	50	3	188	83
4	5	80	20	<10 (LOQ)	92
5	10	80	20	<10 (LOQ)	93
6	5	80	3	<10 (LOQ)	95
7	10	80	3	<10 (LOQ)	95

a **7-K** (500 mg) was stirred with the respective scavenger in 10 volumes of MIBK. The resulting slurry was cooled down to 20 °C and filtered with 50 wt % of Celite using a disposable funnel (10 μm), and the solid was washed with MIBK (3 × 10 volumes). The combined organic filtrate was concentrated under reduced pressure at 50 °C and then dried in a vacuum tray dryer (VTD) at 75 °C for 16 hours for Pd residue measurement. LOQ: Limit of Quantitation.

b Source batch for Pd scavenger weight loading study.

The Pd removal process was thus implemented into the LLE workup, and the 10 wt % Pd scavenger loading was selected for scale-up, affording **7-K** with excellent recovery and <10 ppm Pd. It is worth noting that the potassium level decreased from 4% to approximately 2%, indicating that neutralization occurred during the SiliaMetS SH treatment. Reduction of potassium content also introduced challenges in the subsequent precipitation process. However, a basification step applied to the solution resulting from the SiliaMetS SH treatment successfully restored the potassium level, thereby enabling a smooth and efficient precipitation process.

Several verification batches were conducted on the decagram scale. The results are summarized in Table 4 (entries 1–3, 4–6). These trials highlighted the importance of effective deoxygenation during Heck/Suzuki-Miyaura reaction, as a low yield was obtained when it failed to achieve complete deoxygenation (entries 2, 5). The two atropisomers of **7** remained unchanged before and after purification, with a consistent minor-to-major isomer ratio of approximately 1:5, based on A % (275 nm). This work marks the development of the first one-pot catalytic system for constructing the core structure of the complex lenacapavir molecule.

**Table 4:**
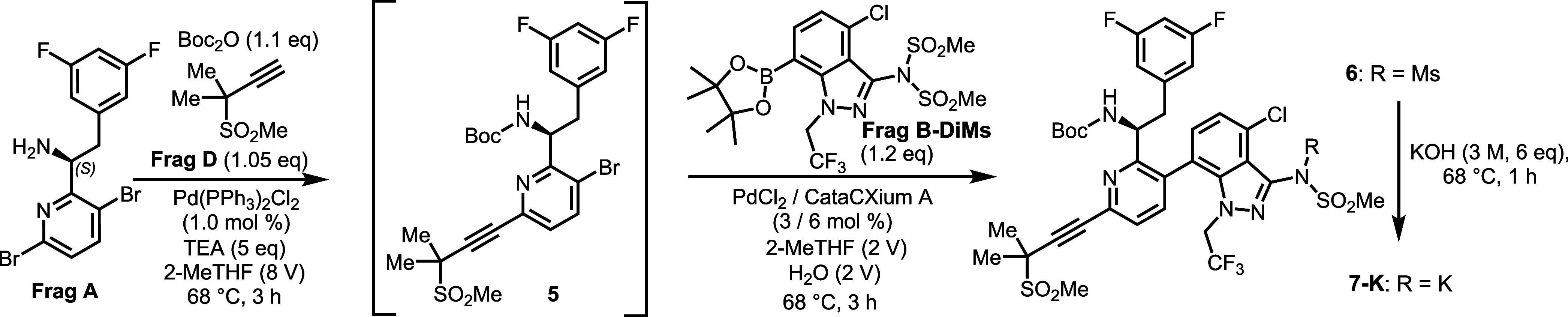
Decagram-Scale Verification of a One-Pot Double-Dose Heck/Suzuki-Miyaura Sequence

	In-process analysis data (A %)^b^									
	Step 1		Step 2							
							Major unknowns Retention Time (min)			
#^a^	**5**	**11**	7^c^	**5**	**11**	**12**	7.2	8.8	12.1	12.8
1^d^	94	6	83.3	3.2	5.1	5.5	0	0	0.4	0.5
2^e^,^f^	90	8	49.5	23.2	10.1	10.1	0.7	-^g^	0.2	0.2
3	94	6	84.0	2.3	4.6	5.8	0.2	0.2	0.6	0.4

	Output after LLE purification										
	HPLC data of isolated compound (A %)^b^										
			Major unknowns Retention Time (min)								
	7^c^	**12**	7.2	8.8	12.1	12.8	Mass (g)	wt %^h^	K (wt %)^i^	IY (%)^j^	Pd (ppm)^k^
4	98.7	0.5	ND	ND	0.8	ND	16.9	79	4.0	69	500^l^
5^e^	97	1.0	0.06	0.3	0.2	0.2	33.1	81	4.2	34	<11.4^m^
6	98.1	0.8	ND	ND	1.1	ND	65	86	4.7	70	8^n^

a All reactions were performed on 40g scale of **Frag A** (1.0 equiv), **Frag D** (1.05 equiv), Boc_2_O (1.1 equiv), and Pd­(PPh_3_)_2_Cl_2_ (1 mol %), unless otherwise stated; **10** was completely consumed in all Heck reactions.

b A % refers to UV area % measured at 275 nm by HPLC. A small amount of other Frag B-DiMs derivatives was observed in all experiments.

c Two atropisomers (minor/major (A %) ∼ 1/5) were observed.

d 10 g of **Frag A** was used, 1.1 eq of **Frag D** was used.

e Low yield due to the failure to achieve complete deoxygenation of the reaction system during Suzuki-Miyaura reaction.

f 1.1 eq of **Frag D** was used.

g Overlapped with **11** (8.5 min).

h Wt % based on free acid **7** was obtained by HPLC, containing 3–8 wt % of MIBK, 1–2 wt % of water.

i Potassium content was measured by LC-ELSD.

j Corrected by wt %.

k Obtained by ICP-OES.

l No retreatment with Pd scavengers.

m Treated with 100 wt % SiliaMetS SH at 80 °C for 3 h.

n Treated with 10 wt % SiliaMetS SH at 80 °C for 3 h. ND = nondetect.

With **7-K** in hand, Boc deprotection was initially carried out using trifluoroacetic acid (TFA),^15^ yielding the corresponding amine **8**. Subsequent coupling with **Frag C** proceeded smoothly, affording **Len-API-H** in >90% isolated yield after column chromatography (Scheme 3). The results supported the feasibility of the proposed synthetic route toward the API. Despite this initial success, several challenges had to be addressed to enable a process suitable for adoption by generic pharmaceutical companies. Although TFA-promoted Boc removal was efficient, the complete elimination of residual TFA during workup proved difficult. Remaining TFA reacts with the amino group in **8** to form trifluoroacetamide during the subsequent amidation, complicating purification of the final API. Increasing regulatory restrictions on PFAS compounds limit the use of TFA in pharmaceutical manufacturing.^41–44^

**Scheme 3:**
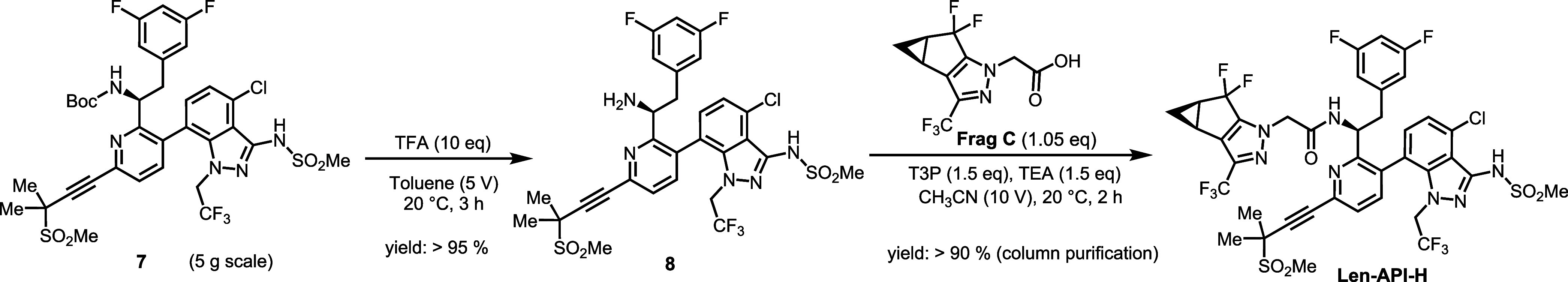
TFA-Promoted Boc-Deprotection and the Subsequent Amidation with **Frag C** for **Len-API-H** Synthesis

To overcome these issues, various mineral acids and methanesulfonic acid (MSA) were evaluated for Boc deprotection of **7-K**.^45^ As shown in Table 5, both H_2_SO_4_ (9M, 10 equiv) and MSA effectively cleaved the Boc group, affording **8** in 96–97 A % (275 nm). In contrast, HCl and H_3_PO_4_ were ineffective in deprotecting **7-K**. Notably, MSA produced a gel-like reaction mixture that complicated purification. Employment of less than 10 equiv or a lower concentration of H_2_SO_4_ was insufficient for complete Boc removal. Solvent screening identified a mixture of toluene (4.5 V) and EtOH (0.5 V) as optimal, providing a homogeneous solution of **7-K** prior to acid addition. Upon addition of H_2_SO_4_, a heterogeneous mixture formed, but the subsequent workup proceeded smoothly, yielding **8**. Single solvents such as MEK and MIBK provided high yields with H_2_SO_4_; concerns over the potential imine formation between the ketone solvent and **8** ultimately led to their deprioritization. EtOH enabled smooth deprotection, but the presence of inorganic salts in the final product led to low wt % purity. Ultimately, the combination of toluene (4.5 V) and EtOH (0.5 V) was selected as the preferred solvent system for the Boc-deprotection process. The free base (neutral) form of **8** was found to be critical for the success of the subsequent amidation step; otherwise, inconsistent amidation outcomes were observed, leading to the formation of API regioisomers and **13** impurity (Scheme S2 in Supporting Information).

**Table 5:**
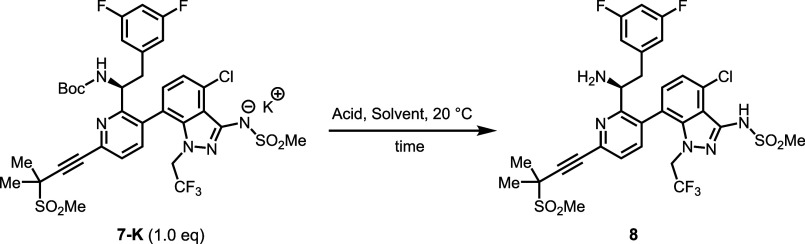
Acid Screening and Optimization for Boc Deprotection of **7-K**

				In-process A%^b^	
#^a^	Acid	Solvent	Time (h)	7-K^c^	8^c^
1	HCl in MeOH (3M, 20 equiv)	DCM (5 V)	24	61	30
2	aq H_3_PO_4_ (4M, 20 equiv)	DCM (5 V)	24	94	ND
3	MSA (10 equiv)	Toluene (5 V)	5	ND	97
4	aq H_2_SO_4_ (9M, 10 equiv)	Toluene (5 V)	5	ND	97
5	aq H_2_SO_4_ (9M, 2 equiv)	Toluene (5 V)	20	24	75
6	aq H_2_SO_4_ (2M, 5 equiv)	Toluene (5 V)	20	97	0.3
7	aq H_2_SO_4_ (5.5M, 8 equiv)	Toluene (5 V)	20	80	17
8	aq H2SO4 (16M, 10 equiv)	Toluene (5 V)	5	ND	97
9	aq H_2_SO_4_ (9M, 10 equiv)	MEK (5 V)	3	ND	97
10	aq H_2_SO_4_ (9M, 10 equiv)	water (5 V)	22	96	<1
11	aq H_2_SO_4_ (9M, 10 equiv)	EtOH (5 V)	6	<1	96
12	aq H_2_SO_4_ (9M, 10 equiv)	MIBK (5 V)	1	ND	98
13^d^	aq H_2_SO_4_ (9M, 10 equiv)	Toluene/EtOH (4.5 V/0.5 V)	1	ND	97
14^e^	aq H_2_SO_4_ (9M, 10 equiv)	Toluene/EtOH (4.5 V/0.5 V)	1	ND	97

a All reactions were performed on 100 mg scale of **7-K**, unless otherwise stated.

b A % refers to UV area % measured at 275 nm, with several other minor peaks observed.

c Two atropisomers (minor/major (A %) ∼ 1/5) were observed.

d 1 g scale of **7-K**.

e 10 g scale of **7-K**.

To address this issue, a practical and robust workup process—based on p*K*_a_-guided pH adjustment for free base isolation—was developed (vide infra) to ensure a reproducible and scalable approach to neutral **8**. The p*K*_a_ of **8** was determined to be between 9.8 and 10.2, guiding the adjustment of the reaction mixture to pH 7–8. This pH range effectively promoted the formation of the free base and enabled efficient extraction into the organic solvent. Among the organic solvents screened at pH 7.5, including EtOAc, iPrOAc, and 2-MeTHF, EtOAc demonstrated excellent solubilization of **8** and enabled clean phase separation. Notably, a single EtOAc extraction efficiently recovered **8** from the reaction mixture. This p*K*_a_-guided pH adjustment strategy proved to be a highly effective and robust approach for the purification of **8**.

For example, upon completion of Boc deprotection, the reaction mixture was diluted with water (1 V) to facilitate efficient phase separation during subsequent EtOAc (15 V) extraction. The acidic organic layer was then treated with NaOH (3M) to achieve pH 7–8. To ensure complete removal of residual Na^+^ and SO_4_^2–^ ions, two water washes were performed, with LC-ELSD monitoring confirming their absence. The resulting organic layer was concentrated and precipitated by the addition of heptane, yielding **8** with 92–97 wt % purity and 94–97% isolated yield. The process was successfully demonstrated on decagram scales and consistently delivered high yields and purity of **8**, as summarized in Table 6. The isolated compound **8** exhibited reproducible reactivity in the subsequent amidation step, confirming the robustness of the workup approach.

**Table 6:**
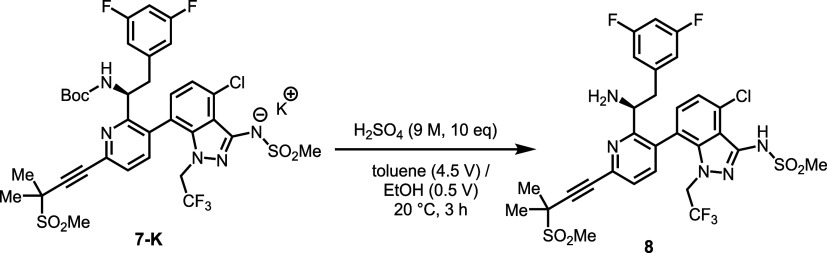
Results Summary of Boc Deprotection and p*K*_a_-Guided LLE Purification of **8** (Decagram Scale)

	**8** after LLE purification							
#^a^	A %^b^,^c^	Mass (g)	Wt %^d^	IY (%)^e^	Pd (ppm)^f^	Res. Sol.	Water (KF)	Salts
1	94	18	97	94	<10	ND	1	ND
2	96	18	92	95	10	-	-	ND
3	97	39	94	97	18	3	1	ND

a All reactions were performed at rt using **7-K** (25 g for entries 1 and 2; 50 g for entry 3) and H_2_SO_4_ (9M, 10 equiv), refer to experimental section for details.

b A % refers to UV area % measured at 275 nm by HPLC, multiple unknown peaks were observed.

c Two atropisomers (minor/major (A %) ∼ 1/5) were observed.

d Wt % was obtained by HPLC.

e Corrected by wt %.

f Obtained by ICP-OES. IY: isolated yield; Res. Sol.: residual solvents; ND = nondetect.

Amidation of **8** with **Frag C** ^21,46^ represents the final step in the synthesis of the target API, making it critical to establish reaction conditions that yield a clean purity profile and controllable impurity levels. Our initial approach to **Len-API-H** synthesis employed T3P-based amide coupling conditions pioneered by Gilead Sciences.^18^ Amidation of **8** with **Frag C** was carried out at 20 °C using T3P (1.5 equiv) as the coupling reagent and TEA (1.5 equiv) as the base (Table 7, entry 1). The desired **Len-API-H** was obtained in 85.9 A % (235 nm), accompanied by 1.3 A % (235 nm) of an overamidation side product **13**, and 2.3 A % (235 nm) of residual **8**. No monoamidation product on the methylsulfonamide (−NHMs) group was detected. Increasing TEA to 3 equiv, in a bid to reduce residual **8**, led to substantial quantities of **13**, even at 0 °C (Table 7, entry 2). Although subsequent NaOH treatment successfully decomposed **13** and removed **Frag C** after liquid–liquid extraction (LLE) workup, **8** persists. Given the p*K*_a_ of ∼10 for the -NHMs group in **8**, we hypothesized that TEA (p*K*_a_ = 10.8) could potentially deprotonate -NHMs and thereby promote undesired amidation to **13**. To achieve complete conversion of **8** while suppressing side reactions at the -NHMs site, our efforts focused on systematic base screening and reaction condition optimization (Table 7). Side reactions at the -NHMs moiety increased with the temperature. At 50 °C, TEA (1.5 equiv) generated 12.9 A % (235 nm) of **13** (Table 7, entry 3). DIPEA (3.0 equiv), with a similar p*K*_a_ to TEA, resulted in 15.4 A % (235 nm) **13** (Table 7, entry 4). NMM (p*K*_a_ = 7.4) emerged as the most reliable base. At −10 °C, 3.0 equiv of NMM delivered **Len-API-H** in 88 A %, with only 1.9 A % (235 nm) **13** (Table 7, entry 5). However, at 20 °C under similar conditions, **13** increased to 12.1% (235 nm) (Table 7, entry 6). Using pyridine (p*K*_a_ = 5.2) as the base, amidation proceeded slowly, yielding **Len-API-H** in 59 A % (235 nm), with significant residual **8** and 8.4 A % (235 nm) **13** (Table 7, entry 7). These findings underscore the importance of using NMM at a low temperature to suppress **13** formation.

**Table 7:**
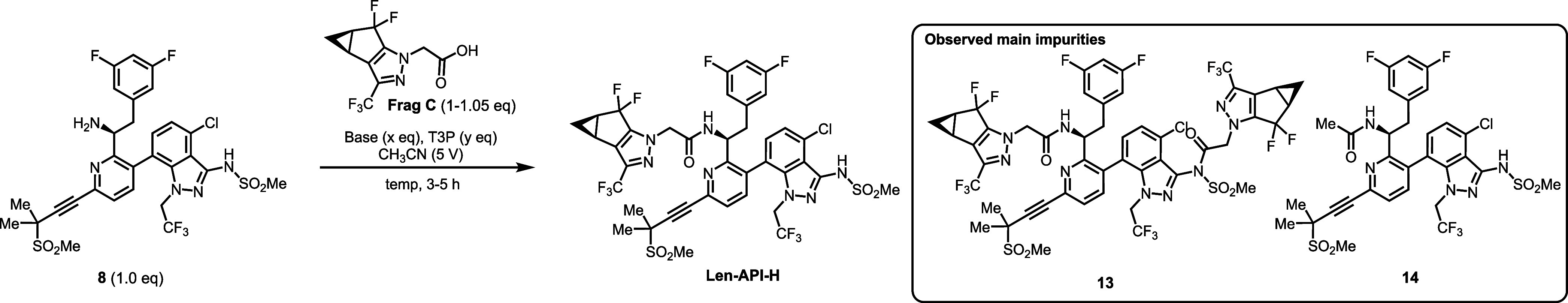
Base Screening and Reaction Condition Optimization for the Synthesis of Len-API

				In-process A % (235 nm)^b^			
#^a^	Base (eq)	T3P (eq)	Temp (°C)	**8**	**Frag C**	Len-API-H^c^	**13**
1	TEA (1.5)	1.5	20	2.3	-	85.9	1.3
2	TEA (3.0)	1.5	0	0.3	-	71.6	22.2
3	TEA (1.5)	1.5	50	0.7	3.2	74.7	12.9
4	DIPEA (3.0)	1.5	–10	0.4	0.7	78.2	15.4
5	NMM (3.0)	1.5	–10	2.2	2.0	88.0	1.9
6	NMM (3.0)	1.5	20	3.6	1.7	66.7	12.1
7	Py (3.0)	1.5	0	14.2	3.7	59.0	8.4
8	NMM (1.0)	1.0	–10	25.5	5.2	65.6	0.45
9	NMM (2.0)	1.25	–10	5.8	2.1	85.1	1.1
10	NMM (2.0)	1.25	–10	5.4	3.6	82.9	1.1
11	NMM (2.2)	1.25	–10	1.2	2.0	86.7	2.5

a All reactions were performed with **8** (0.5 g, 1 equiv), **Frag C** (1.05 equiv), T3P (50 wt % in DMF), base under conditions shown in the table.

b A % refers to UV area % measured at 275 nm by HPLC, with several other small unknown impurities along with 0.2–0.3 A % **14** in all reactions.

c Two atropisomers (minor/major (A %) ∼ 1/6) were observed.

Further optimization focused on varying the equivalents of NMM and T3P (Table 7, entries 8–11). The optimal conditions—2.2 equiv of NMM and 1.25 equiv of T3P at −10 °C—enabled reproducible high conversion of **8** with NMT 3 A % of **13**. Under these conditions, **Len-API-H** was obtained in 86.7 A % (235 nm), with 1.2 A % (235 nm) residual **8** and 2.5 A % (235 nm) **13** (Table 7, entry 11). Prolonging the reaction time did not improve the conversion. Despite achieving a manageable level of **13**, the presence of 1–2 A % (235 nm) **8** became a challenge for **Len-API-Na** purification. Following LLE workup and recrystallization, **8** could only be diminished to 0.2–0.5 A % (235 nm). Therefore, reducing **8** during amidation proved critical to achieving levels below 0.1 A % (235 nm) in the final API. Further optimization of the reaction conditions revealed that stepwise addition of T3P was essential to suppress **8** below 0.1 A % (235 nm). Specifically, an initial charge of T3P (1.0 equiv) was added to a mixture of **8** (1.0 equiv), **Frag C** (1.02 equiv), and NMM (3.0 equiv) at −10 °C, followed by heating to 60 °C for 4–5 h. In-process analysis (HPLC) of the reaction mixture showed approximately 2–3 A % (235 nm) of **8** remained. A second and third portion of T3P (0.3 and 0.2 equiv, respectively) were then introduced at 60 °C, which enabled complete conversion of **8** after 18 h. After amidation, the mixture was treated with 1 M aqueous NaOH (10 equiv) at 45 °C for 2 h, followed by partitioning into MIBK. The MIBK layer was concentrated and precipitated with heptane to yield the crude API with a sodium content of 2 wt % (measured by LC-ELSD), consistent with theoretical expectations based on the crude API composition (85 wt %). The crude product showed 94–97 A % (235 nm); however, under these conditions, **14** emerged as the predominant impurity (∼0.9 A %, 235 nm) (Table 8, entries 1 and 2). Its level was reduced to below 0.5 A % (235 nm) after recrystallizations from EtOH (10 V)/heptane (20 V) (Table 8, entries 3 and 4), and further reduced to below 0.2 A % (235 nm) after trituration from MIBK (5 V)/toluene (5 V) at 80 °C (Table 8, entry 5). Detailed LC-MS analysis of the advanced intermediates revealed that **14** was generated during the EtOAc workup in the Boc deprotection step and subsequently carried over into the API. Replacing EtOAc with 2-MeTHF during this workup effectively suppressed the formation of **14**, resulting in an API free of this impurity following amidation. Due to time constraints, further optimization and scale-up of this modified process were not fully pursued at this stage.

**Table 8:**
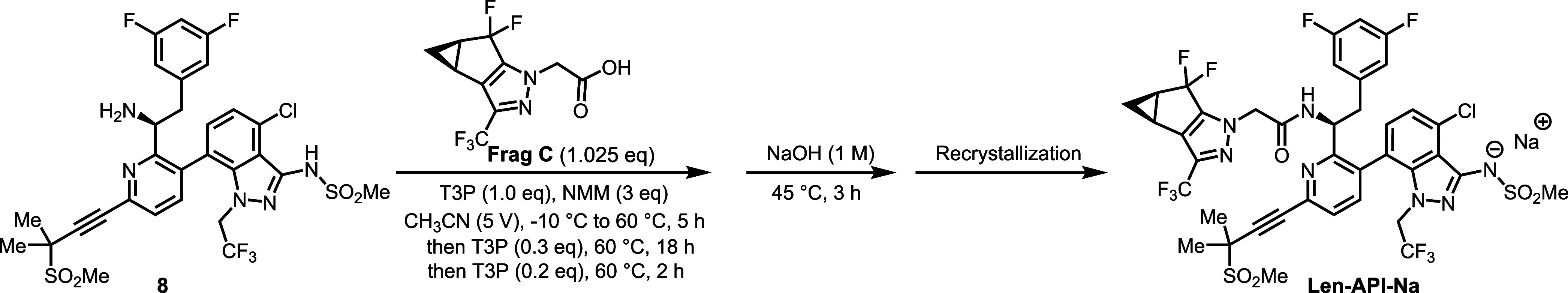
Synthesis of **Len-API-Na** and Purification by Recrystallization

**Synthesis of Len-API-Na**										
	Input **8**			Output crude Len-API-Na^b^,^c^						
#^a^	Mass (g)	Wt %	A %	IY%	Mass (g)	A %	Wt %	Na wt %	**14** (A %)	**8** (A %)
1	11	92	98	85	15	94.0	81	2.0	0.5	<0.2
2	35	94	97	92	53	93.7	81	2.0	1.0	0.3

**Characterization data of purified Len-API-Na after recrystallization/trituration**										
							A % (HPLC, 235 nm)			
		Mass (g)	IY%^e^	Wt %	Pd (ppm)^f^	Na wt %	**Len-API-Na**	**8**	**14**	12.89 min
3^d^	Recrystallization	10	70	>99.9	<4.2	1.96	98.9	0.1	0.6	0.3
4^d^		37.5	78	>99.9	<4.2	1.88	98.8	0.2	0.6	0.4
5^g^	Trituration	0.42	85	98.7	-	-	99.6	ND	0.2	0.2

a All amidations were carried out with **8** (1.0 equiv) and **Frag C** (1.025 equiv) under conditions shown in the scheme and table.

b Na content was measured by LC-ELSD, and wt % of the free acid material was measured by HPLC unless otherwise stated. A% refers to UV area % measured at 235 nm by HPLC, with corrected isolated yield (IY).

c Two atropisomers (minor/major (A%) ∼1/6) were observed.

d Crude material was recrystallized twice from EtOH (10 V)/heptane (20 V) from 80 to 20 °C for overnight.

e Corrected isolated yield (IY) after recrystallization.

f Pd content measured by ICP-OES.

g Material (0.5 g) from entry 3 was triturated from MIBK (5 V)/toluene (5 V) from 80 °C for 2 h then cooled to 20 °C for 3 h.

## Conclusions

In summary, relative to the established scale-up process,^18^ the new approach offers several key advantages: 1) The one-pot Heck/Suzuki-Miyaura sequence reduces the number of purification-requiring reaction steps from four to three; 2) The telescoped design lowers total Pd usage from 5 mol % to 4 mol % and consolidates Pd removal into a single operation rather than the three required previously; 3) Purification operations on intermediates are reduced from three to two; 4) The process allows for late-stage incorporation of the costly **Frag C**, thereby decreasing overall synthesis expenses. Building on these improvements, we developed a novel, streamlined, and efficient synthetic process for the production of lenacapavir API. A sequential Heck/Suzuki-Miyaura coupling strategy was employed for the first-time synthesis of the key intermediate **7-K**. A cost-effective and process-friendly Boc deprotection method was established, followed by a final-step amidation with **Frag C** to complete the synthesis of lenacapavir API.

An early stage Pd removal step ensured that the final API contained less than 4.2 ppm of residual Pd. This overall process delivers lenacapavir API in up to 55% overall yield with a purity up to >99.9 wt % and ∼99.6 A % (235 nm) after two recrystallizations. We hope these results will empower generic pharmaceutical companies to produce lenacapavir more efficiently and affordably, ultimately expanding access to patients in low- and middle-income countries.

## Experimental Section

### General Information

Reagents and solvents were obtained from commercial suppliers and used as received, unless otherwise indicated. Where applicable, reactions were conducted in oven-dried (120 °C) glassware, which was assembled while hot and cooled to ambient temperature under an inert atmosphere. Reactors were prerinsed with reaction solvent and subjected to evacuation/backfill cycles (3×) as necessary. All reactions were conducted under an inert atmosphere (nitrogen) unless otherwise noted. Reactions were monitored by TLC (precoated silica gel 60 F254 plates, EMD Chemicals), Agilent HPLC, GCMS, and Agilent GC-FID using various methods. GC-FID was used for the analysis of heptane and toluene levels. The sample was prepared in an acetonitrile/methanol mixed diluent in duplicate. Quantitation was performed by using a calibration curve. Ethyl acetate was used as an internal standard, and QC standards were analyzed throughout the sequence. The samples for Pd analysis were prepared in 10% HCl and analyzed by ICP-OES. A calibration curve for analysis was prepared with QC standards that were analyzed throughout the sequence. Weight assay was measured by LC-DAD. Salt content was measured by LC-ELSD. Water content was measured by KF titration. A % was measured by HPLC at 275 or 235 nm. Melting point was measured with a Stuart SMP10 melting point apparatus. TLC was visualized with UV light or by treatment with phosphomolybdic acid (PMA), ninhydrin, and/or KMnO_4_.^1^H NMR and ^13^C NMR spectra were routinely recorded on Bruker Avance III HD Ascend 600 MHz spectrometer. All chemical shifts are reported in parts per million (ppm) relative to residual DMSO (2.50 ppm for ^1^H, 39.52 ppm for ^13^C) or CHCl_3_ (7.26 ppm for ^1^H, 77.16 ppm for ^13^C). Coupling constants (J) are reported in hertz (Hz). The following abbreviations were used to designate signal multiplicity: s, singlet; d, doublet; t, triplet; q, quartet; p, pentet; dd, doublet of doublets; ddd, doublet of doublet of doublets; dt, double of triplets; ddt, doublet of doublet of triplets; m, multiplet; br, broad. Advanced intermediates **Frag A**, **Frag B**, and **Frag C-EE** were prepared according to reported methods.^21,23,25^

#### Synthesis of *N*-(4-Chloro-7-(4,4,5,5-tetramethyl-1,3,2-dioxaborolan-2-yl)-1-(2,2,2-trifluoroethyl)-1H-indazol-3-yl)-*N*-(methylsulfonyl)­methanesulfonamide (Frag B-Dims)

A 5L ChemRxnHub reactor was degassed with N_2_ and charged with **Frag B** (222.00 g, 561.53 mmol, 95.0 wt %), 2-MeTHF (2.2 L, 10 V), and triethylamine (250.0 mL, 1.80 mol, 3.2 equiv) at 25 °C. The mixture was stirred, and the temperature control unit (TCU) was set to −3.0 °C. Once the internal temperature reached NMT 1.0 °C, methanesulfonyl chloride (109.0 mL, 1.40 mol, 2.5 equiv) was added slowly over 20 min, maintaining the internal temperature at NMT 10.0 °C. The mixture was heated to 20 °C and stirred at the same temperature for 2 h. After the reaction was complete (monitored by TLC and ^1^H NMR), the resulting suspension was filtered, and the filter cake was washed with heptane (890 mL, 4 V). The solid was then transferred to the reactor, stirred in 2-MeTHF (890 mL, 4 V) for 30 min, and filtered. The filter cake was washed with 2-MeTHF (230 mL, 1 V). The filtrate was concentrated to 2–3 V, precipitated by addition of heptane (5 V). The suspension was stirred for 30 min and then filtered. The resulting solid was transferred to the reactor, stirred in deionized H_2_O (5 V) for 30 min, and filtered. The filter cake was washed with heptane (1 V) and dried *in vacuo* at 60 °C until constant weight to yield **Frag B-DiMs** (281.00 g, 100 wt % by qNMR) as a white solid (94.1% corrected yield).

^1^H NMR (600 MHz, DMSO-*d*_6_) δ 7.95 (d, *J* = 7.6 Hz, 1H), 7.50 (d, *J* = 7.6 Hz, 1H), 5.94 (q, *J* = 8.7 Hz, 2H), 3.66 (s, 6H), 1.37 (s, 12H).

^13^C NMR (151 MHz, DMSO-*d*_6_) δ 170.9, 143.6, 135.0, 125.9 (q, *J* = 281 Hz), 118.5, 110.7, 60.3, 49.9 (q, *J* = 35 Hz), 43.0, 21.2, 14.5.

^19^F NMR (565 MHz, DMSO-*d*_6_) δ −69.70 (t, *J* = 8.8 Hz, 3F).

A % (275 nm): 97.7%

HPLC wt % purity (275 nm): 100.3%

KF water content analysis: 0.023%

LC-MS (*m*/*z*) (M+H): 532

IR (ATR) νmax: 3045, 3017, 2988, 2935, 1573, 1416, 1398, 1368, 1351, 1331, 1314, 1264, 1245, 1206, 1161, 1105, 1090, 988, 967, 939, 887, 857, 833, 813, 773, 757, 703, 691

HRMS (ESI) *m*/*z*: calcd for C_17_H_22_BClF_3_N_3_O_6_S_2_·Na^+^= [M + Na]^+^ 554.0576, found 554.0588

Melting point: 220–222 °C.

#### Synthesis of 2-((3bs,4ar)-5,5-Difluoro-3-(trifluoromethyl)-3b,4,4a,5-tetrahydro-1H-cyclopropa­[3,4]­cyclopenta­[1,2-*c*]­pyrazol-1-yl)­acetic Acid (Frag C)

To a three-neck 1000 mL round-bottom flask was added **Frag C-EE** (56.00 g, 172.4 mmol, 1.0 eq, 95.5 wt %, 100%ee), EtOH (33.6 mL, 0.6 V), and 224 mL of H_2_O (4 V). Then, an aqueous solution of KOH (31.4 mL, 31 wt %, 224.1 mmol, 1.3 equiv) was added, and the mixture was stirred at 50 °C for overnight. After the reaction was complete, the mixture was added dropwise to a precooled aqueous HCl solution (1.7 M, 263.7 mL, 448.2 mmol, 2.6 equiv) at 0 °C. The resulting suspension was filtered, and the filter cake was washed with H_2_O (250 mL, 4.5 V) and dried under vacuum to yield **Frag C** (44.00 g, 95.8 wt % by qNMR, 100% ee) as a white solid (86.7% corrected yield).

^1^H NMR (600 MHz, DMSO-*d*_6_) δ 13.52 (brs, 1H), 5.02 (dd, *J* = 18.0, 44.0 Hz, 2H), 2.70–2.60 (m, 2H), 1.44–1.48 (m, 1H), 1.05–1.03 (m, 1H).

^13^C NMR (151 MHz, DMSO-*d*_6_) δ 167.8, 142.7 (t, *J* = 29.0 Hz), 134.0 (q, *J* = 39.0 Hz), 132.4 (m), 120.7 (q, *J* = 268.3 Hz), 120.2 (t, *J* = 243.4 Hz), 52.2, 27.6 (dd, *J* = 29.0, 5.7 Hz), 23.4, 11.7.

^19^F NMR (565 MHz, DMSO-*d*_6_) δ −60.3 (s, 3F), −79.8 (d, *J* = 253.0 Hz, 1F), −102.8 (d, *J* = 253.0 Hz, 1F).

A % (235 nm): 99.0%

HPLC wt % purity (235 nm): 100.2%

KF water content analysis: 0.009%

LC-MS (*m*/*z*) (M+H): 283

IR (ATR) νmax: 3075, 3014, 2965, 1767, 1735, 1538, 1443, 1411, 1390, 1344, 1321, 1273, 1241, 1182, 1129, 1109, 1047, 1016, 952, 926, 839, 805, 759, 714, 669

HRMS (ESI) *m*/*z*: calcd for C_10_H_7_F_5_N_2_O_2_·H^+^= [M + H]^+^ 283.0506, found 283.0504

Melting point: 149–151 °C.

#### Synthesis of Potassium (S)-(7-(2-(1-((tert-Butoxycarbonyl)­amino)-2-(3,5-difluorophenyl)­ethyl)-6-(3-methyl-3-(methylsulfonyl)­but-1-yn-1-yl)­pyridin-3-Yl)-4-chloro-1-(2,2,2-trifluoroethyl)-1H-indazol-3-yl)­(methylsulfonyl)­amide (7-K)

A 1L ChemRxnHub reactor was sequentially charged with **Frag A** (40.00 g, 100.1 mmol, 98.1 wt %, 98.8% ee), **Frag D** (15.84 g, 105.1 mmol, 1.05 equiv, 97 wt %), and bis­(triphenylphosphine)­palladium­(II) dichloride (0.70 g, 1.0 mmol, 0.01 equiv). The reactor was evacuated and backfilled with argon four times, then charged with degassed 2-MeTHF (360 mL, 7 V), a solution of di*tert*-butyl dicarbonate (24.27 g, 110.1 mmol, 1.1 equiv) in 2-MeTHF (40 mL, 1 V), and degassed triethylamine (70 mL, 500 mmol, 5.0 equiv). The contents were stirred and heated to 68 °C and held for 2–3 h. Once the Heck coupling was completed, degassed deionized H_2_O (40 mL, 1 V) was added to the reaction mixture.

A 2L ChemRxnHub reactor was successively charged with **Frag B-DiMs** (63.87 g, 120.1 mmol, 1.2 equiv, 100 wt %), CataCXium A (2.27 g, 6.00 mmol, 0.06 equiv), and palladium­(II) dichloride (0.53 g, 3.00 mmol, 0.03 equiv). The reactor was evacuated and backfilled with argon four times, then charged with degassed 2-MeTHF (80 mL, 2 V), and the contents stirred at 20 °C. The contents in Reactor 1 were then transferred to Reactor 2. Following complete transfer, a deionized H_2_O (40 mL, 1 V) rinse was added to Reactor 1 and transferred to Reactor 2. The contents of Reactor 2 were heated to 68 °C and stirred for 3 h. Once the Suzuki-Miyaura reaction was complete, 3 M KOH (200 mL, 600 mmol, 6.0 equiv) was added to Reactor 2 and stirred for 1 h at the same temperature. Upon hydrolysis completion, the contents were cooled to 20 °C.

The contents were filtered through a Celite plug (20 g) and returned to a clean reactor. Once the aqueous phase was separated, the organic phase was concentrated at 50 °C (NLT 100 Torr), diluted with deionized H_2_O (800 mL, 20 V), and further concentrated until the volatile organics were removed (NLT 55 Torr). The aqueous phase was pH-adjusted to pH ≥ 12.5 with aq. KOH (160 mL, 3 wt %) and washed thrice with MTBE (200 mL, 5 V each). The aqueous phase was then extracted once with MIBK (400 mL, 10 V), and the organic phase was treated with SilaMetS Thiol (8.40 g, 10 wt % of theoretical yield) as a slurry at 80 °C for 3 h. The contents were cooled to 20 °C, filtered through a Celite plug (20 g), and rinsed with MIBK (200 mL, 5 V). The filtrate is returned to a clean reactor, washed successively with 3 M KOH (40 mL, 1 V) and deionized H_2_O (40 mL, 1 V), concentrated at 50 °C (NLT 30 Torr) to ∼200 mL batch volume, and precipitated with heptane (400 mL, 10 V) over NLT 10 min. The contents were cooled to 20 °C, filtered, and the solid was dried in a vacuum oven at 75 °C, 30 Torr, for NLT 12 h to yield **7-K** (65.05 g, 90.4 wt % by HPLC) as a light-yellow solid (69.7% corrected yield).

^1^H NMR (600 MHz, DMSO-*d*_6_) δ 7.74–7.67* (m, 2H), 7.41–7.26* (d, J = 6 Hz, 1H), 7.08–6.94* (m, 3H), 6.51–6.41* (m, 2H), 4.33–4.03* (m, 3H), 3.36 (s, 3H), 3.24–3.23* (m, 3H), 2.86–2.77* (m, 4H), 1.72 (brs, 6H), 1.26–1.21* (s, 9H). *Signals from minor atropisomer included.

^13^C NMR (151 MHz, DMSO-*d*_6_) δ: 208.3, 162.8 (d, J = 13.6 Hz), 161.2 (d, J = 13.6), 160.7, 155.5, 152.1, 143.2–143.1 (m), 140.9, 139.8, 139.3, 131.4, 129.9, 128.2, 126.3, 124.7, 122.8, 119.2, 117.6, 117.5, 111.9–111.8 (m), 102.0–101.6 (m), 87.5, 84.8, 78.2, 57.3, 55.5, 51.8, 35.1, 30.1, 28.1, 24.9, 23.9, 22.5, 22.3.

^19^F NMR (565 MHz, DMSO-*d*_6_) δ −68.3 (t, J = 8.7 Hz, 1F), −68.6 (m, 0.3H)*, −68.8 (t, J = 9.3 Hz, 3F), −110.4 – −110.5 (m, 2F), −110.6 – −110.7* (m, 0.5F), −110.8 – −110.9* (m, 0.2F), −111.0 –

–111.1* (m, 0.2F). *Signals from minor atropisomer included.

A % (275 nm): 98.1%

Atropisomeric ratio based on A % (275 nm): major: 83.2% (retention time: 10.11 min); minor atropisomer: 16.8% (retention time: 9.76 min)

HPLC wt % purity (275 nm): 7 free acid (85.7 wt %)

GC-FID solvent analysis: MIBK (3.90 wt %); Heptane (0.13 wt %)

ICP-OES metals analysis: Pd (7.9 ppm)

KF water content analysis: H_2_O (1.32 wt %)

ELSD salt content analysis: 4.7 wt %

LC-MS (*m*/*z*) [M - K + H]^+^: 804

IR (ATR) νmax: 2978, 2935, 1703, 1625, 1595, 1577, 1508, 1482, 1366, 1305, 1228, 1198, 1157, 1103, 1042, 977, 841, 760

HRMS (ESI) *m*/*z*: calcd for C_34_H_34_ClF_5_KN_5_O_6_S_2_·H^+^= [M + H]^+^ 842.1269, found 842.1264

Melting point: 210–215 °C.

#### Synthesis of (S)-N-(7-(2-(1-Amino-2-(3,5-difluorophenyl)­ethyl)-6-(3-methyl-3-(methylsulfonyl)­but-1-yn-1-yl)­pyridin-3-yl)-4-chloro-1-(2,2,2-trifluoroethyl)-1H-indazol-3-yl)­methanesulfonamide (8)

To a 500 mL ChemRxnHub reactor was added **7-K** (50.00 g, 53.66 mmol, 1.0 eq, 90.4 wt %), toluene (225 mL, 4.5 V), and EtOH (25 mL, 0.5 V). The reaction was stirred for 20–30 min at 20 °C under a N_2_ atmosphere to obtain a clear solution. After which, 9 M sulfuric acid (59.6 mL, 536.6 mmol, 10 equiv) was added dropwise via syringe, and the reaction was stirred at 20 °C until Boc-deprotection was completed. After 2 h, EtOAc (750 mL, 15 V) and H_2_O (75 mL, 1.5 V) were added and stirred for 10 min. The layers were separated, and the organic layer was basified (pH ∼8.5) using 3 M NaOH (20 mL, 0.4 V). The layers were separated, and the organic layer was washed two times with deionized H_2_O (250 mL, 5 V each). The final organic layer was concentrated at 55 °C (NLT 50 Torr) to an ∼50 mL batch volume (1 V). Heptane (500 mL, 10 V) was added, and the slurry was stirred for 30 min. After which, the solid was collected via filtration using a disposable funnel, suction dried for 30 min and then dried in a vacuum oven at 75 °C, 30 Torr, for NLT 12 h to afford **8** (39 g, 94.1 wt % by HPLC) as a light-yellow solid (97% corrected yield).

^1^H NMR (600 MHz, DMSO-*d*_6_) (**Major isomer**) δ: 7.74 (d, *J* = 8.0 Hz, 1H), 7.71 (d, *J* = 7.9 Hz, 1H), 7.21 (d, *J* = 7.6 Hz, 1H), 7.03 – 6.93 (m, 1H), 6.63 (d, *J* = 7.6 Hz, 1H), 6.31 (dd, *J* = 8.3, 2.1 Hz, 2H), 4.91 (td, *J* = 16.8, 8.5 Hz, 1H), 4.32 (td, *J* = 16.9, 8.5 Hz, 1H), 3.62 (t, *J* = 6.8 Hz, 1H), 3.25 (s, 3H), 3.16 (s, 3H), 2.91 – 2.74 (m, 2H), 1.73 (s, 6H).

^1^H NMR (600 MHz, DMSO-*d*_6_) (**Minor isomer**) δ: 7.82 (d, J = 8.0 Hz, 1H), 7.71 (d, J = 7.9 Hz, 1H), 7.42 (d, J = 7.7 Hz, 1H), 7.36 (d, J = 7.7 Hz, 1H), 7.02 – 6.93 (m, 1H), 6.60 (dd, J = 8.3, 2.0 Hz, 1H), 6.31 (dd, J = 8.3, 2.1 Hz, 1H), 4.50 (dt, J = 24.3, 8.3 Hz, 1H), 4.10 – 3.96 (m, 1H), 3.70 (dd, J = 8.6, 5.1 Hz, 1H), 3.25 (s, 3H), 3.16 (s, 3H), 2.98 (dd, J = 13.4, 8.7 Hz, 1H), 2.90 – 2.83 (m, 1H), 1.73 (s, 6H).

^13^C NMR (151 MHz, DMSO-*d*_6_) δ 163.3 (d, *J* = 13.3 Hz), 161.7 (d, *J* = 13.4 Hz), 161.1, 143.0, 142.2, 141.5 (d, *J* = 177.2 Hz), 140.1, 139.95 – 139.8 (m), 139.7, 131.1 (d, *J* = 106.3 Hz), 129.0 (d, *J* = 104.6 Hz), 124.6, 122.7, 122.4, 119.7, 118.2, 117.8, 112.4 (d, *J* = 24.3 Hz), 102.2 (t, *J* = 25.6 Hz), 88.6, 85.1, 57.8, 55.3, 50.7 (d, *J* = 33.5 Hz), 44.4, 41.6 (s), 41.4, 35.5 (d, *J* = 3.6 Hz), 31.7, 28.8, 22.8 – 22.7 (m), 22.6, 14.4. (Atropisomers cannot be differentiated in ^13^CNMR).

^19^F NMR (565 MHz, DMSO-*d*_6_) (**Major isomer**) δ −68.95 (t, *J* = 8.6 Hz, 3F), −110.42 (t, *J* = 7.8 Hz, 2F).

^19^F NMR (565 MHz, DMSO-*d*_6_) (**Minor isomer**) δ −68.75 (t, *J* = 8.3 Hz, 3F), −110.42 (t, *J* = 7.8 Hz, 2F).

A % (275 nm): 96.8%

Atropisomeric ratio based on A % (275 nm): major: 83.0% (retention time: 7.77 min); minor atropisomer: 17.0% (retention time: 6.14 min)

HPLC wt % purity (275 nm): 94.1%

GC-FID solvent analysis: 2.49% Heptane; 0.40% Toluene

ICP-OES metals analysis: 18.2 ppm

KF water content analysis: 0.83%

ELSD salt content analysis: Not detected

LC-MS (*m*/*z*) [M + H]^+^: 704

IR (ATR) νmax: 3360, 3248, 3021, 2935, 1625, 1593, 1577, 1500, 1446, 1357, 1303, 1262, 1239, 1154, 1115, 1044, 973, 937, 885, 848, 829, 759

HRMS (ESI) *m*/*z*: calcd for C_29_H_27_ClF_5_N_5_O_4_S_2_·H^+^= [M + H]^+^ 704.1186, found 704.1183

Melting point: 126–127 °C.

#### Synthesis of Sodium (4-Chloro-7-(2-((S)-1-(2-((3bs,4ar)-5,5-difluoro-3-(trifluoromethyl)-3b,4,4a,5-tetrahydro-1H-cyclopropa­[3,4]­cyclopenta­[1,2-*c*]­pyrazol-1-yl)­acetamido)-2-(3,5-difluorophenyl)­ethyl)-6-(3-methyl-3-(methylsulfonyl)­but-1-yn-1-yl)­pyridin-3-Yl)-1-(2,2,2-trifluoroethyl)-1H-indazol-3-yl)­(methylsulfonyl)­amide (Len-API-Na)

To a 1000 mL ChemRxnHub Reactor 1 was added **8** (35 g, 1 eq, 46.72 mmol, 94 wt %) and **Frag C** (13.5 g, 1.025 eq, 47.89 mmol, 100 wt %). Reagent-grade acetonitrile (175 mL, 5 V) was added to Reactor 1, and the mixture is stirred at 25 °C under N_2_. The mixture was then cooled to an internal temperature of −10 °C. 4-Methylmorpholine (15.9 mL, 3.0 eq, 140.2 mmol) was added slowly (8 mL/min) to Reactor 1, at −10 °C (internal) under N_2_ and stirred for NLT 10 min, after which, a 50% solution of T3P (27.8 mL, 1.0 eq, 46.72 mmol, 50 wt %) in DMF was added slowly (8 mL/min) to Reactor 1 at −10 °C under N_2_. The reactor was then warmed to 25 °C and further heated to NLT 60 °C and stirred for NLT 4.5 h. After the allotted time, a second lot of 50% T3P (8.2 mL, 0.3 equiv, 14.0 mmol, 50 wt %) in DMF was added slowly (8 mL/min) to Reactor 1 at 60 °C under N_2_. The reactor was maintained under these conditions for NLT 18 h. After 18 h, a third lot of 50% T3P (5.44 mL, 0.2 equiv, 9.3 mmol, 50 wt %) in DMF was added slowly (8 mL/min) to Reactor 1 at 60 °C under N_2_. The reactor was maintained under these conditions for NLT 1 h.

The reaction mixture was basified (pH ∼ 13.7) with 1 M NaOH (383 mL, 8 equiv, 374 mmol) and then stirred and heated to 45 °C (internal) for NLT 3 h. To this reaction mixture, MIBK (358 mL, 10 V), and the mixture was stirred for NLT 15 min. A phase separation was undertaken, and the aqueous layer was discharged. The organics were concentrated via distillation (250 to 50 mbar) to 40 mL, ∼1 V, and then heptane (530 mL, 15 V) was added and stirred for 1 h. The resulting solid was filtered using a disposable fritted funnel and suction-dried for 1 h. The solid was transferred to 2000 mL of ChemRxnHub Reactor 2. To this, EtOH (530 mL, 10 V based on crude solid) was added and stirred at 70 °C for 15 min until a clear solution was observed. Then heptane (1060 mL, 20 V based on crude solid) was added dropwise over 3 h maintaining temperature at 70 °C. The mixture was stirred at 70 °C for 30 min and then slowly cooled to room temperature over 16 h. The solid was filtered with a disposable fritted funnel, washed with 1:2 EtOH:Heptane (530 mL, 10 V), and suction-dried for NLT 1 h. The obtained solid was charged back to Reactor 2. To this, EtOH (600 mL, 10 V based on crude solid) was added and stirred at 70 °C for 15 min until a clear solution was observed. Then heptane (1200 mL, 20 V based on crude solid) was added dropwise over 3 h maintaining temperature at 70 °C. The mixture was stirred at 70 °C for 30 min and then slowly cooled to room temperature over 16 h. The solid was filtered with a disposable fritted funnel, washed with 1:2 EtOH:Heptane (600 mL, 10 V), suction-dried for NLT 1 h and finally oven-dried at 75 °C under vacuum (30 Torr) for NLT 12 h until constant weight to obtain pure **Len-API-Na** as a yellow solid (37.5 g, 78.4% corrected yield, 100.6 ± 2.8 wt %).

^1^H NMR (600 MHz, DMSO*-d*_6_) (**Major isomer**) δ: 9.08 (d, *J* = 8.0 Hz, 1H), 7.69 (s, 2H), 7.04 – 6.96 (m, 1H), 6.93 (d, *J* = 7.6 Hz, 1H), 6.67 (d, *J* = 7.6 Hz, 1H), 6.44 (d, *J* = 6.3 Hz, 2H), 4.89 (d, *J* = 16.5 Hz, 1H), 4.76 (d, *J* = 16.5 Hz, 1H), 4.69 (td, *J* = 8.4, 5.1 Hz, 1H), 4.19 (dq, *J* = 16.6, 8.3 Hz, 1H), 3.81 (dq, *J* = 17.6, 8.9 Hz, 1H), 3.25 (s, 3H), 2.93 (ddd, *J* = 22.5, 13.6, 7.0 Hz, 2H), 2.78 (s, 3H), 2.61 – 2.51 (m, 2H), 1.74 (s, 3H), 1.73 (s, 3H), 1.39 (dd, *J* = 13.4, 7.2 Hz, 1H), 0.94 (d, *J* = 3.3 Hz, 1H).

^1^H NMR (600 MHz, DMSO-*d*_6_) (**Minor isomer**) δ: 8.92 (d, *J* = 8.5 Hz, 1H), 7.75 (dd, *J* = 21.8, 7.9 Hz, 2H), 6.95 (d, *J* = 12.0 Hz, 1H), 6.91 – 6.85 (m, 2H), 6.52 (d, *J* = 6.3 Hz, 2H), 4.83 – 4.78 (m, 2H), 4.63 (d, *J* = 16.5 Hz, 1H), 4.28 (dd, *J* = 16.0, 8.2 Hz, 1H), 3.73 (dd, *J* = 16.5, 8.1 Hz, 1H), 3.24 (s, 3H), 3.09 – 3.00 (m, 2H), 2.83 – 2.79 (s, 3H), 2.62 – 2.51 (m, 2H), 1.73 (d, J = 3.6 Hz, 6H), 1.45 – 1.41 (m, 1H), 0.98 (s, 1H).

^13^C NMR (151 MHz, CDCl3-*d*) δ 164.4, 163.4, 163.0, 162.9, 161.3, 161.2, 158.9, 158.2, 152.6, 151.9, 143.0 – 142.9 (m), 142.9 – 142.8 (m), 142.8 – 142.7 (m), 142.6, 142.4, 142.4 – 142.2 (m), 142.1 (d, *J* = 9.4 Hz), 141.4 (s), 141.1, 139.6 (s), 139.4, 134.0 (d, *J* = 38.6 Hz), 133.6, 133.2 – 133.0 (m), 132.1, 131.9, 131.7, 129.7, 129.6, 128.2, 127.9, 126.9, 126.8, 124.5, 123.5 – 123.1, 122.7, 121.6, 121.6 – 121.5 (m), 119.9, 119.9, 119.8, 118.9, 118.2 (d, *J* = 21.1 Hz), 117.5, 117.2, 117.0, 112.1, 111.97, 111.8, 102.2, 102.0, 101.8, 88.1, 88.0, 84.7, 57.3, 56.0, 53.2, 53.0, 52.8, 52.4, 49.5 (d, *J* = 32.1 Hz), 40.0, 38.6, 35.1, 27.7, 27.6, 27.3, 23.2, 22.3 (d, *J* = 18.7 Hz), 11.6. (Atropisomers cannot be differentiated in ^13^CNMR).

^19^F NMR (565 MHz, DMSO-*d*_6_) (**Major isomer**) δ: −60.01 (s, 3F), −68.55 (s, 3F), −79.33 (d, *J* = 254.0 Hz, 1F), −102.61 (d, *J* = 253.9 Hz, 1F), −109.89 (s, 2F).

^19^F NMR (565 MHz, DMSO-*d*_6_) (**Minor isomer**) δ: −59.97 (s, 3F), −68.03 (s, 3F), −79.58 (d, *J* = 254.2 Hz, 1F), −102.58 (d, *J* = 253.7 Hz, 1F), −109.97 (s, 2F).

A % (235 nm): 98.8A %

Atropisomeric ratio based on A % (235 nm): major: 85.9% (retention time: 11.96 min); minor atropisomer: 14.1% (retention time: 11.42 min)

HPLC wt % purity (235 nm): 100.6% ± 2.8%

GC-FID solvent analysis: 0.3% ± 0.04% (EtOH); 0.06% ± 0.01% (Heptane)

ICP-OES metals analysis (Pd content): < 4.2 ppm

KF water content analysis: 0.3% ± 0.01%

ELSD salt content analysis: 1.88% ± 0.08%

LC-MS (*m*/*z*) [M – Na + H]^+^: 968

IR (ATR) νmax: 3651, 3366, 3021, 2943, 1702, 1627, 1590, 1515, 1485, 1459, 1385, 1366, 1351, 1314, 1260, 1236, 1148, 1131, 1109, 1042, 1018, 978, 945, 842, 807, 762

HRMS (ESI) *m*/*z*: calcd for C_39_H_31_ClF_10_N_7_NaO_5_S_2_·H^+^= [M + H]^+^ 990.1327, found 990.1326

Melting point: 238–240 °C

${[\alpha ]}_{D}^{20}$ ­(deg·mL·g^–1^·dm^–1^) (MeOH (10 mg/mL) at 20 °C under 589 nm): −93.044.

## Supplementary Material



## Notes

Scheme S1, Scheme S2, Table S1, p*K*_a_ measurements of **7-K** and **8**, experimental details, synthesis and characterization of impurities, calorimetric data, procedure for process safety assessment, analytical methods, and spectroscopic data (PDF)
