# Supplementary material for: A Concise Process to Lenacapavir Sodium: Demonstration of One-Pot Sequential Heck/Suzuki-Miyaura Couplings in Lenacapavir API Synthesis
Source: Org Process Res Dev. 2026 Mar 3;30(3):692–706. doi: 10.1021/acs.oprd.5c00443 (PMC13010305; doi:10.1021/acs.oprd.5c00443)
Supplement: Supplementary file 1 [file op5c00443_si_001.pdf]

Supporting Information

A Concise Process to Lenacapavir Sodium: Demonstration of One-Pot Sequential Heck/Suzuki-Miyaura Couplings in Lenacapavir API Synthesis

*Daryl Guthrie,‡ Anand H. Shinde,‡ Ramesh Vediyappan, ‡ Aline Nunes De Souza, ‡ Rama Krishna Sayini, ‡ John M. Saathoff, ‡ Nagaraju Sakkani, Naeem Asad, Samuel R. Hochstetler, Barrack Stubbs, Justina M. Burns, B. Frank Gupton, Ryan Littich, Limei Jin**

Medicines for All Institute, Virginia Commonwealth University, Richmond, VA, 23284-3068.

‡These authors contributed equally

*Email: jinl3@vcu.edu

Medicines for All Institute, Virginia Commonwealth University, Richmond, VA, 23284-3068.

Contents

1. GENERAL METHODS S-2
2. Scheme S1 .... S-3
3. Scheme S2 .... S-3
4. Table S1 .... S-3
5. p*K*a measurement of 7 .... S-4
6. p*K*a measurement of 8 .... S-5
7. Synthesis of key intermediates S-6
8. Telescoped synthesis of API S-12
9. Synthesis and characterization of major impurities S-19
10. Process safety assessment S-24
11. Analytical Method Development S-29
12 NMR and HPLC Spectra S-55

1. GENERAL METHODS

Reagents and solvents were obtained from commercial suppliers and used as received unless otherwise indicated. Where applicable, reactions were conducted in oven-dried (120 °C) glassware, which was assembled while hot, and cooled to ambient temperature under an inert atmosphere. Reactors were pre-rinsed with reaction solvent and subjected to evacuation/back-fill cycles (3×) as necessary. All reactions were conducted under an inert atmosphere (Nitrogen) unless otherwise noted. Reactions were monitored by TLC (precoated silica gel 60 F254 plates, EMD Chemicals), Agilent HPLC, GCMS, or Agilent GC-FID using various methods. GC-FID was used for analysis of heptane and toluene levels. The sample was prepared in an acetonitrile/methanol mixed diluent, in duplicate. Quantitation was performed using a calibration curve. Ethyl acetate was used as an internal standard and QC standards were analyzed throughout the sequence. The samples for Pd analysis were prepared in 10% HCl and analyzed by ICP-OES. A calibration curve for analysis was prepared with QC standards analyzed throughout the sequence. Weight Assay was measured by LC-DAD. Salt Content was measured by LC-ELSD. Water content was measured by KF titration. A% was measured by HPLC at 275 nm or 235 nm. Melting point was measured with a Stuart SMP10 melting point apparatus. TLC was visualized with UV light or by treatment with phosphomolybdic acid (PMA), ninhydrin, and/or $KMnO_4$. ^{1}H NMR and ^{13}C NMR spectra were routinely recorded on Bruker Avance III HD Ascend 600 MHz spectrometer. All chemical shifts are reported in parts per million (ppm) relative to residual DMSO (2.50 ppm for ^{1}H, 39.52 ppm for ^{13}C) or $CHCl_3$ (7.26 ppm for ^{1}H, 77.16 ppm for ^{13}C). Coupling constants J are reported in hertz (Hz). The following abbreviations were used to designate signal multiplicity: s, singlet; d, doublet; t, triplet; q, quartet, p, pentet; dd, doublet of doublets; ddd, doublet of doublet of doublets; dt, double of triplets; ddt, doublet of doublet of triplets; m, multiplet; br, broad. Advanced intermediates **Frag A**, **Frag B**, and **Frag C-EE** were prepared according to Y1 Len PDRs.[1–3]

2. Scheme S1

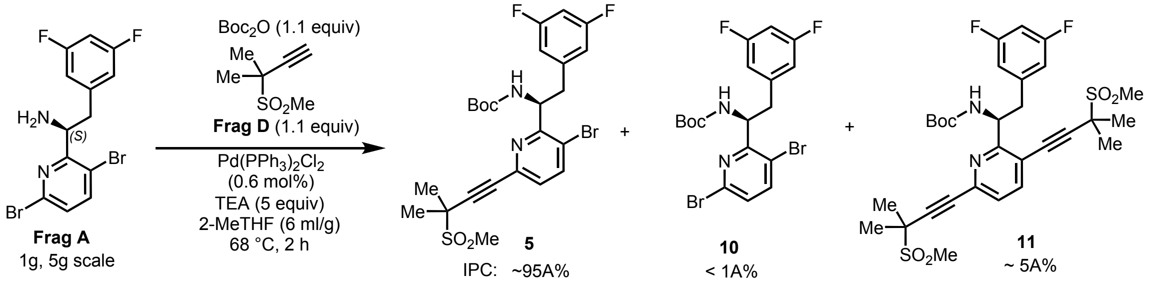

Scheme S1 Evaluation of one-pot *in-situ* Boc protection and Heck reaction with **Frag A**.

3. Scheme S2

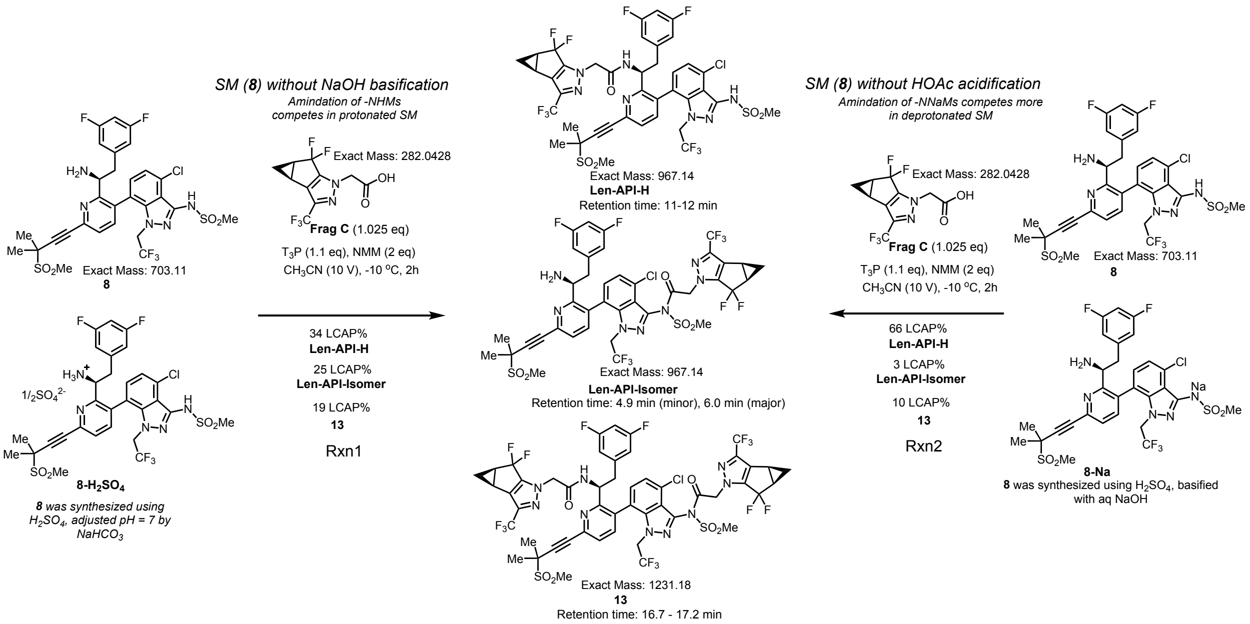

Scheme S2 Purity profile of amidation using protonated or deprotonated **8** as the starting material. Major impurities of **Len-API-Isomer** and **13** were formed.

4. Table S1

Table S1 Pd scavenger screen for removal of Pd residue from crude **7-K**.

#[a]	Scavenger (0.2 g)	A%, (275 nm)	Pd (ppm)[b]	Recovery yield
1	Source batch	97	**395**[c]	NA
2[d]	aq NAC (20 mol%)	98	61	ND
3[e]	aq PIX (20 mol%)	98	89	ND
4	Source batch	97	**261**[c]	NA
5	(CYS) Cysteine	95	105	85%

6	(DMT) Dimercaptotriazine	95	18	88%
7	(IMI) Imidazole	95	21	88%
8	(THU) Thiourea	96	16	89%
9	(TRI) Triamine	94	59	84%
10	(SH) Thiol	94	13	74%

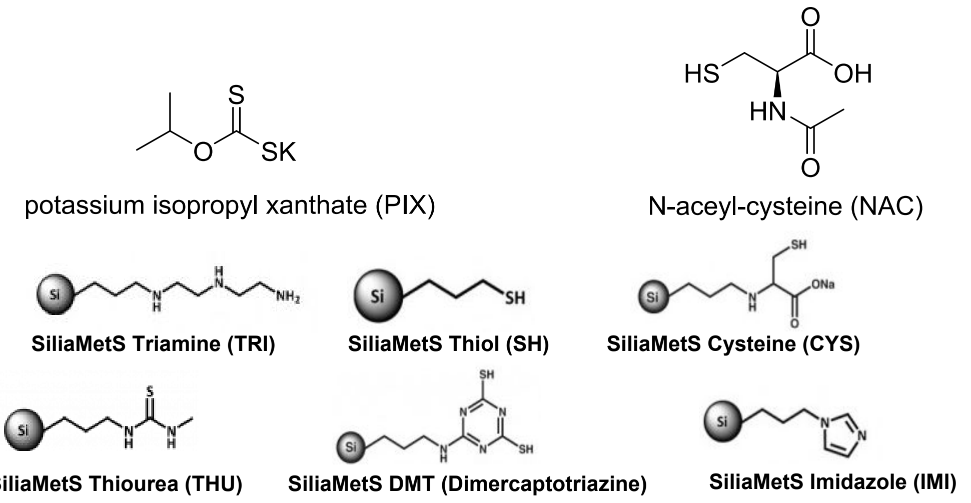

[a]**7-K** (200 mg) was stirred with the respective scavenger (1:1, wt/wt) in 10 volumes of MIBK at 50 °C for 2 hours, unless otherwise stated in the table. The resulting slurry was cooled down to 20 °C and filtered using a disposable funnel (10 micron), and the solid was washed with MIBK (3 × 10 volumes). The combined organic filtrate was concentrated under reduced pressure at 50 °C and then dried in a vacuum tray dryer (VTD) at 75 °C for 16 hours for Pd residue measurement; [b]Pd content was measured by ICP-OES; [c]No Pd scavenger treatment; [d]A mixture of **7-K** in MIBK and 20 mol% NAC in 3% w/w KOH at 50 °C for 2h; [e]A mixture of **7-K** in MIBK and 20 mol% PIX in 3% w/w KOH at 50 °C for 2h.

5. p*K*a measurement of 7

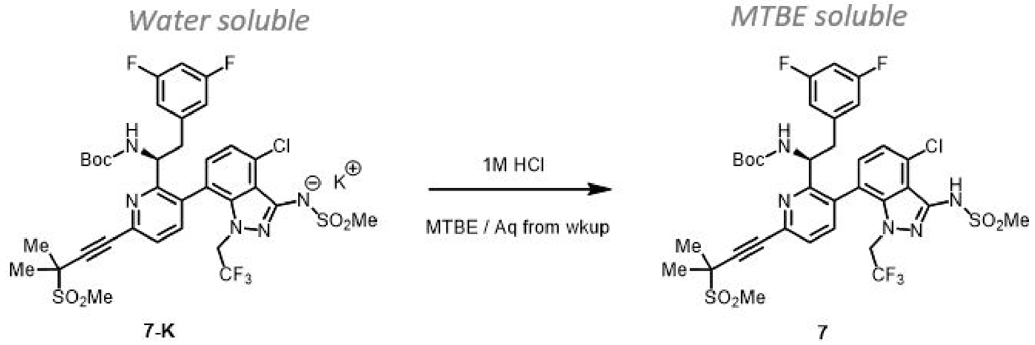

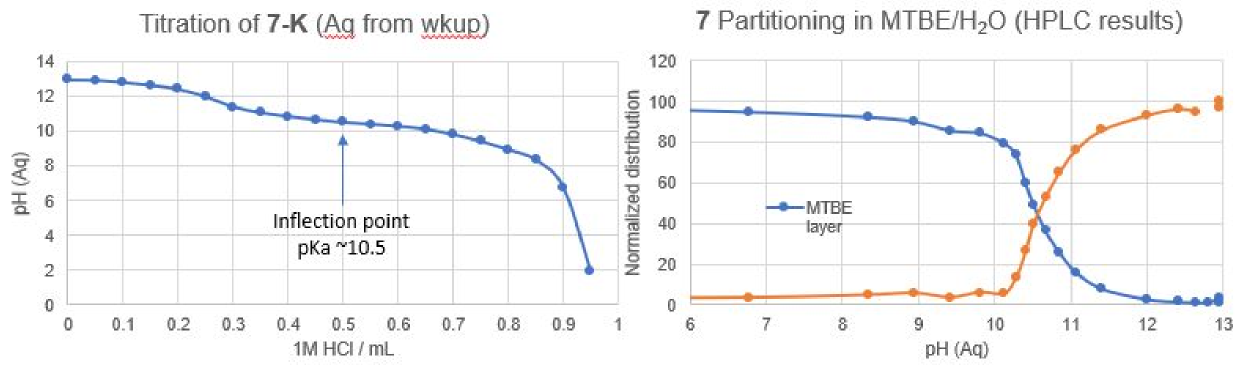

Figure S1 p*K*a measurement of **7** and partitioning in MTBE and H_2O layer. MTBE effectively rejects **7-K**, while readily dissolving organic impurities, at pH > 12.5. Titration experiment: 0.1M **7-K** in 5 mL MTBE / 10 mL water, titrated with 1M HCl at 21°C, after each 0.1 mL addition of HCl, both organic and aqueous layers were sampled (30 µL into 1.00 mL CH_3CN) for HPLC analysis. Peak areas were normalized using the first and last data points. Inflection point in the titration curve corresponds to a p*K*a of ~10.5 for **7**.

6. p*K*a measurement of 8

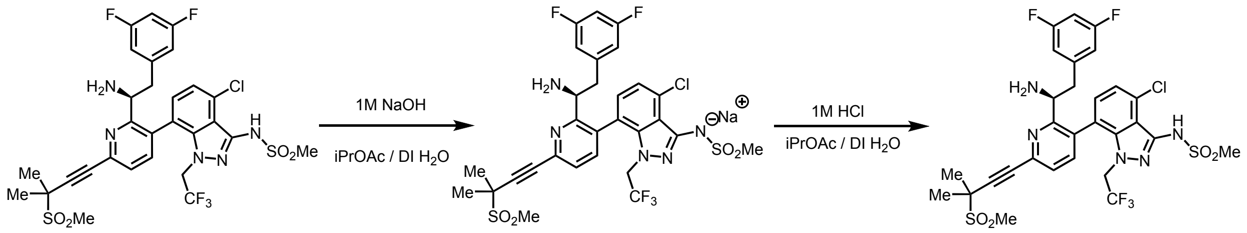

8 **8-Na** **8**

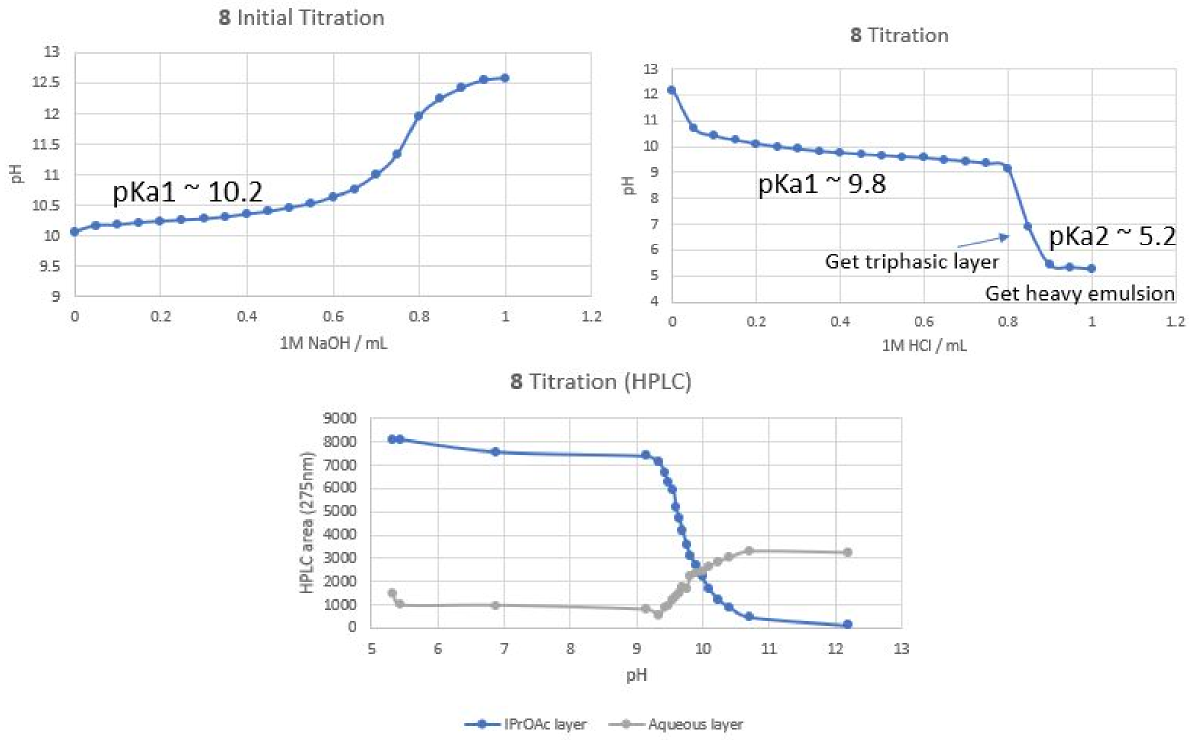

Figure S2 p*K*a measurement of **8** and partitioning in iPrOAc and H_2O layer. iPrOAc effectively dissolves **8** at pH < 9. Titration experiment: 0.1M **8** in 5 mL iPrOAc / 10 mL water, titrated with 1M HCl at 21°C, after each 0.05 mL addition of HCl, both organic and aqueous layers were sampled (30 µL into 1.00 mL CH_3CN) for HPLC analysis. Inflection point in the titration curve corresponds to a p*K*a of ~10.2 for **8** during basification, while a p*K*a of ~9.8 was observed during acidification.

7. Synthesis of key intermediates

Synthesis of *N*-(4-chloro-7-(4,4,5,5-tetramethyl-1,3,2-dioxaborolan-2-yl)-1-(2,2,2-trifluoroethyl)-1H-indazol-3-yl)-*N*-(methylsulfonyl)methanesulfonamide (Frag B-DiMs)

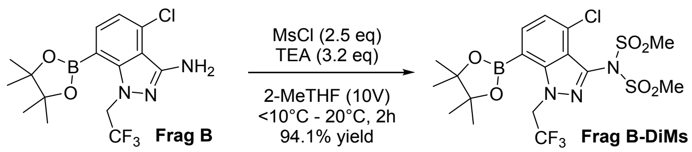

A 5L ChemRxnHub reactor was degassed with N_2 and charged with **Frag B**[2] (222.00 g, 561.53 mmol, 95.0 wt%), 2-MeTHF (2.2 L, 10V), and triethylamine (250.0 mL, 1.80 mol, 3.2 eq) at 25 °C. The mixture was stirred, and the temperature control unit (TCU) was set to -3.0 °C. Once the

internal temperature reached NMT 1.0 °C, methanesulfonyl chloride (109.0 mL, 1.40 mol, 2.5 eq) was added slowly over 20 minutes, maintaining the internal temperature at NMT 10.0 °C. The mixture was heated to 20 °C and stirred at the same temperature for 2 h. After the reaction was complete (monitored by TLC and ^{1}H NMR), the resulting suspension was filtered and the filter cake was washed with heptane (890 mL, 4V). The solid was then transferred to the reactor, stirred in 2-MeTHF (890 mL, 4V) for 30 min, and filtered. The filter cake was washed with 2-MeTHF (230 mL, 1V). The filtrate was concentrated to 2-3V, precipitated by addition of heptane (5V). The suspension was stirred for 30 min and then filtered. The resulting solid was transferred to the reactor, stirred in deionized H_2O (5V) for 30 min, and filtered. The filter cake was washed with heptane (1V) and dried *in vacuo* at 60 °C until constant weight to yield **Frag B-DiMs** (281.00 g, 100 wt% by qNMR) as a white solid (94.1% corrected yield).

^{1}H NMR (600 MHz, DMSO-d_6) δ 7.95 (d, J = 7.6 Hz, 1H), 7.50 (d, J = 7.6 Hz, 1H), 5.94 (q, J = 8.7 Hz, 2H), 3.66 (s, 6H), 1.37 (s, 12H).

^{13}C NMR (151 MHz, DMSO-d_6) δ 170.9, 143.6, 135.0, 125.9 (q, J = 281 Hz), 118.5, 110.7, 60.3, 49.9 (q, J = 35 Hz), 43.0, 21.2, 14.5.

^{19}F NMR (565 MHz, DMSO-d_6) δ -69.70 (t, J = 8.8 Hz, 3F).

A% (275 nm): 97.7 %

HPLC wt% purity (275 nm): 100.3 %

KF water content analysis: 0.023 %

LC-MS (m/z) (M+H): 532

IR (ATR) νmax: 3045, 3017, 2988, 2935, 1573, 1416, 1398, 1368, 1351, 1331, 1314, 1264, 1245, 1206, 1161, 1105, 1090, 988, 967, 939, 887, 857, 833, 813, 773, 757, 703, 691

HRMS (ESI) m/z: calcd for $C_{17}H_{22}BClF_3N_3O_6S_2\cdot Na^+$= $[M+Na]^+$ 554.0576, found 554.0588

Melting point: 220-222 °C.

Synthesis of 2-((3bS,4aR)-5,5-difluoro-3-(trifluoromethyl)-3b,4,4a,5-tetrahydro-1H-cyclopropa[3,4]cyclopenta[1,2-c]pyrazol-1-yl)acetic acid (Frag C)

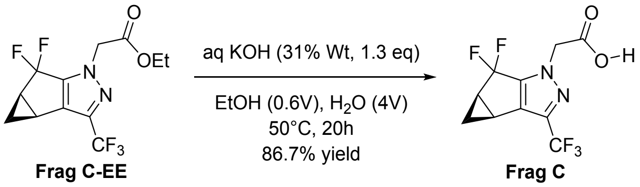

To a three-neck 1000 mL round-bottom flask was added **Frag C-EE**[1] (56.00 g, 172.4 mmol, 1.0 eq, 95.5 wt%, 100%ee), EtOH (33.6 mL, 0.6V) and 224 mL of H_2O (4V). Then, an aqueous solution of KOH (31.4 mL, 31 wt%, 224.1 mmol, 1.3 eq) was added, and the mixture was stirred at 50 °C for overnight. After reaction was complete, the mixture was added dropwise to a pre-cooled aqueous HCl solution (1.7M, 263.7 mL, 448.2 mmol, 2.6 eq) at 0 °C. The resulting suspension was filtered, and the filter cake was washed with H_2O (250 mL, 4.5V) and dried under vacuum to yield **Frag C** (44.00 g, 95.8 wt% by qNMR, 100% ee) as a white solid (86.7% corrected yield).

^{1}H NMR (600 MHz, DMSO-d_6) δ 13.52 (brs, 1H), 5.02 (dd, J = 18.0, 44.0 Hz, 2H), 2.70 – 2.60 (m, 2H), 1.44 – 1.48 (m, 1H), 1.05 – 1.03 (m, 1H).

^{13}C NMR (151 MHz, DMSO-d_6) δ 167.8, 142.7 (t, J = 29.0 Hz), 134.0 (q, J = 39.0 Hz), 132.4 (m), 120.7 (q, J = 268.3 Hz), 120.2 (t, J = 243.4 Hz), 52.2, 27.6 (dd, J = 29.0, 5.7 Hz), 23.4, 11.7.

^{19}F NMR (565 MHz, DMSO-d_6) δ -60.3 (s, 3F), -79.8 (d, J = 253.0 Hz, 1F), -102.8 (d, J = 253.0 Hz, 1F).

A% (235 nm): 99.0 %

HPLC wt% purity (235 nm): 100.2 %

KF water content analysis: 0.009 %

LC-MS (m/z) (M+H): 283

IR (ATR) νmax: 3075, 3014, 2965, 1767, 1735, 1538, 1443, 1411, 1390, 1344, 1321, 1273, 1241, 1182, 1129, 1109, 1047, 1016, 952, 926, 839, 805, 759, 714, 669

HRMS (ESI) m/z: calcd for $C_{10}H_7F_5N_2O_2{\cdot}H^+$= $[M+H]^+$ 283.0506, found 283.0504

Melting point: 149-151 °C.

Synthesis of 3-methyl-3-(methylsulfonyl)but-1-yne (Frag D)[4]

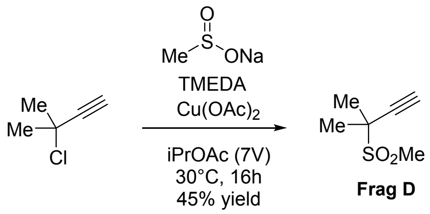

To a 500 mL round-bottom flask was added sodium methanesulfinate (36.29 g, 341.26 mmol, 1.4 eq, 96 wt%), copper (II) acetate (2.26 g, 12.19 mmol, 0.05 eq, 98 wt%), TMEDA (3.67 mL, 24.38 mmol, 0.1 eq, 99.5 wt%), and isopropyl acetate (175 mL, 7V). The suspension was agitated at <25 °C, and 3-chloro-3-methylbut-1-yne (25.00 g, 27.4 mL, 243.76 mmol) was added dropwise to maintain a temperature < 25 °C. The reaction mixture was then heated at 30 °C for 16 h, cooled to RT, diluted with isopropyl acetate (125 mL, 5V), and washed twice with deionized H_2O (125 mL, 5V each). The combined aqueous layers were extracted thrice with isopropyl acetate (125 mL, 5V each). The combined organic layers were washed with water (125 mL, 5V), brine (125 mL, 5V), and concentrated in vacuo to give a solid. The solid was then dried under a high vacuum at RT to yield **Frag D** (17.00 g, 94.05 wt% purity by GCMS) as a white solid (45% corrected yield).

All Frag D batches and commercial batches of Fragment D (Ambeed) were combined and purified through recrystallization to afford a singular batch to use in API process development.

Procedure: **Frag D** (165 g, 88 wt% by GCMS) was suspended in MTBE (1.65 L, 10V). The mixture was heated to 55 °C until dissolved, stirred for 20 minutes, and filtered (hot-filtration) to remove a brown sticky solid. The solution was cooled to 0 °C and stirred for an additional 2 h. The resultant solid was filtered and washed with cold MTBE. The solid was dried overnight in a high vacuum to yield purified **Frag D** (130 g, 97.3 wt% by GCMS) as a white solid.

^{1}H NMR (600 MHz, DMSO-d_6) δ 3.70 (s, 1H), 3.10 (s, 3H), 1.54 (s, 6H)

^{13}C NMR (151 MHz, DMSO-d_6) δ 82.1, 77.4, 56.5, 34.5, 22.3.

GCMS TIC A%: 99.2%

GCMS wt% purity: 97.3 wt%

GC-FID solvent analysis: MTBE 0.67 wt%

IR (ATR) νmax: 3263, 3034, 3017, 2991, 2937, 1452, 1415, 1316, 1293, 1217, 1165, 1113, 964, 945, 800, 757, 719, 669

Melting point: 123-125 °C.

Synthesis of *tert*-butyl (1-(3,6-dibromopyridin-2-yl)-2-(3,5-difluorophenyl)ethyl)carbamate ((*rac*)-10):

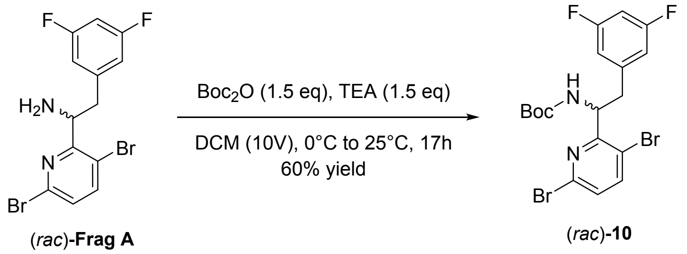

To a 250 mL round-bottom flask was charged (*rac*)-**Frag A** (10.0 g, 25.51 mmol) and DCM (100 mL, 10V). The reaction mixture was cooled to 0 ^{o}C, TEA (5.33 mL, 38.3 mmol, 1.5 eq) and di-tert-butyl dicarbonate (8.35 g, 38.3 mmol, 1.5 eq) were added in order. The reaction mixture was allowed to warm to RT while stirring for 17 hours. After completion, the solvent was evaporated via distillation to afford a crude solid. The crude solid was dissolved in EtOAc (2V), and hexane (8V) was added dropwise to precipitate (*rac*)**-10** (7.90 g, 95.0 wt% by qNMR) as a white solid (60.0% corrected yield).

^{1}H NMR (600 MHz, DMSO-d_6) δ 7.99 (d, *J* = 8.25 Hz, 1H), 7.51 (dd, *J* = 8.44, 12.65 Hz, 2H), 7.07 (t, *J* = 9.35 Hz, 1H), 7.01 (d, *J* = 6.60 Hz, 2H), 5.05 (dt, *J* = 4.40, 9.08 Hz, 1H), 2.86-2.99 (m, 2H), 1.29 (s, 9H).

^{13}C NMR (151 MHz, CDCl3-*d*) δ 162.9 (d, *J* = 13.2 Hz), 161.3 (d, *J* = 13.2 Hz), 160.0, 155.2, 143.6, 142.7 (t, *J* = 9.5 Hz), 139.6, 128.5, 119.3, 112.3 (dd, *J* = 20.0, 4.4 Hz), 101.9 (t, *J* = 25.8 Hz), 56.6, 55.6, 38.3, 28.0, 27.6.

^{19}F NMR (565 MHz, DMSO-d6) δ -110.59 (s, 2F).

A% (275 nm): 99.4 %

LC-MS (m/z) $[M+H]^+$: 491

IR (ATR) νmax: 3379, 2982, 2935, 1690, 1629, 1593, 1459, 1441, 1420, 1368, 1347, 1308, 1269, 1252, 1239, 1174, 1129, 1111, 1055, 1008, 971, 928, 891, 848, 829, 777, 762, 725

HRMS (ESI) m/z: calcd for $C_{18}H_{18}Br_2F_2N_2O_2 \cdot Na^+$= $[M+Na]^+$ 512.9595, found 512.9452

Melting point: 130-132 °C.

Synthesis of *tert*-butyl (1-(3-bromo-6-(3-methyl-3-(methylsulfonyl)but-1-yn-1-yl)pyridin-2-yl)-2-(3,5-difluorophenyl)ethyl)carbamate ((*rac*)-5)

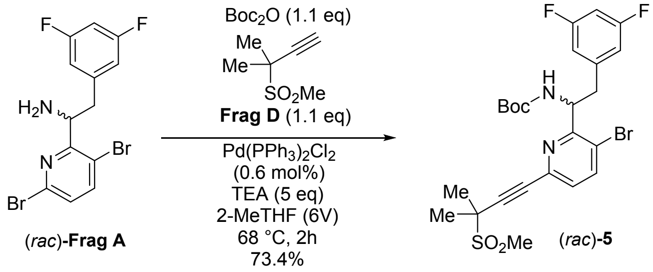

To a 25 mL round-bottom flask was charged (*rac*)-**Frag A** (1.0 g, 2.55 mmol), **Frag D** (410.2 mg, 2.81 mmol, 1.1 eq), bis(triphenylphosphine)palladium (II) dichloride (10.7 mg, 15.3 μmol, 0.006 eq), and degassed 2-MeTHF (5.0 mL, 5V). The mixture was evacuated and backfilled with N_2 three times. Then degassed TEA (1.78 mL, 12.8 mmol, 5.0 eq) was added under N_2 atmosphere at 20 °C, followed a solution of di-tert-butyl dicarbonate (612.4 mg, 2.81 mmol, 1.1 eq) in degassed 2-MeTHF (1 mL, 1V). The reaction mixture was heated to 68 °C and maintained for 2 h. Upon completion, the reaction mixture was partitioned between EtOAc (20V) and deionized H_2O (20V). The aqueous layer was extracted thrice with EtOAc (10V each). The combined organic layers were washed thrice with deionized H_2O (10V each), brine (10V), and concentrated to dryness. The resulting crude solid was dissolved in EtOAc (2V), and then hexane (8V) was added dropwise to yield (*rac*)-**5** as a brown solid (1.04 g, 73.4 % uncorrected yield).

^{1}H NMR (600 MHz, DMSO-d_6) δ 8.10 (d, J = 8.07 Hz, 1H), 7.47 (dd, J = 8.40, 13.20 Hz, 2H), 7.07 (t, J = 9.45 Hz, 1H), 7.02 (d, J = 6.42 Hz, 2H), 5.08 - 5.13 (m, J = 4.03 Hz, 1H), 3.20 (s, 3H), 2.86 - 2.97 (m, 2H), 1.68 (s, 6H), 1.28 (s, 9H).

^{13}C NMR (151 MHz, DMSO-d_6) δ 163.0, 161.3, 159.2, 155.2, 142.9, 141.3, 139.7, 128.0, 120.1, 112.4, 101.9, 88.0, 84.1, 78.1, 69.0, 57.2, 55.8, 38.5, 35.0, 28.0, 22.3, 22.2.

^{19}F NMR (565 MHz, DMSO-d_6) δ -110.64 (m, 2F).

A% (275 nm): 97.6 %

LC-MS (m/z) (M+H): 557

IR (ATR) νmax: 3373, 3008, 2982, 2932, 1702, 1627, 1597, 1519, 1448, 1431, 1392, 1366, 1305, 1269, 1256, 1241, 1165, 1116, 1060, 1038, 1016, 971, 855, 766

8. Telescoped synthesis of API

Telescoped synthesis of 7-K (Step 1: Coupling Fragments A, D, and B-DiMs)

Synthesis of potassium (S)-(7-(2-(1-((tert-butoxycarbonyl)amino)-2-(3,5-difluorophenyl)ethyl)-6-(3-methyl-3-(methylsulfonyl)but-1-yn-1-yl)pyridin-3-yl)-4-chloro-1-(2,2,2-trifluoroethyl)-1H-indazol-3-yl)(methylsulfonyl)amide (7-K)

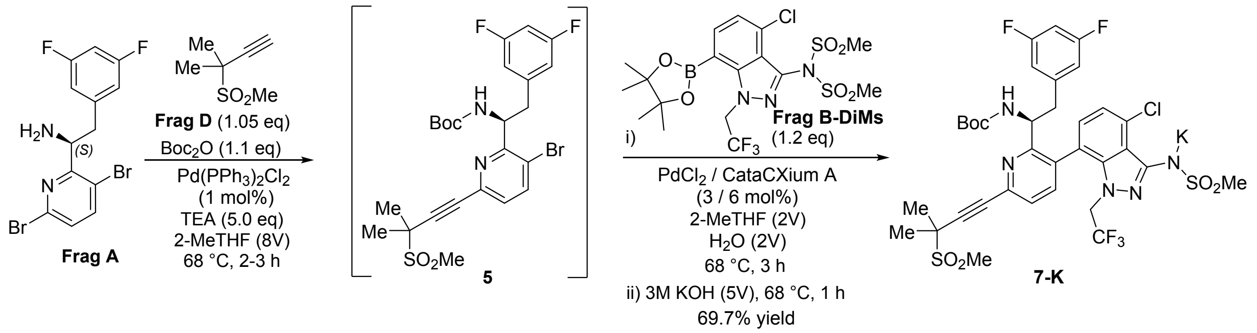

A 1L ChemRxnHub reactor was sequentially charged with **Frag A**[3] (40.00 g, 100.1 mmol, 98.1 wt%, 98.8 %ee), **Frag D** (15.84 g, 105.1 mmol, 1.05 eq, 97 wt%), and bis-(triphenylphosphine)palladium (II) dichloride (0.70 g, 1.0 mmol, 0.01 eq). The reactor was evacuated and backfilled with Argon four times, then charged with degassed 2-MeTHF (360 mL, 7V), a solution of di-tert-butyl dicarbonate (24.27 g, 110.1 mmol, 1.1 eq) in 2-MeTHF (40 mL, 1V), and degassed triethylamine (70 mL, 500 mmol, 5.0 eq). The contents were stirred and heated to 68 °C and held for 2-3 hours. Once the Heck coupling was completed, degassed deionized H_2O (40 mL, 1V) was added to the reaction mixture.

A 2L ChemRxnHub reactor was successively charged with **Frag B-DiMs** (63.87 g, 120.1 mmol, 1.2 eq, 100 wt%), CataCXium A (2.27 g, 6.00 mmol, 0.06 eq), and palladium (II) dichloride (0.53 g, 3.00 mmol, 0.03 eq). The reactor was evacuated and backfilled with Argon four times, then charged with degassed 2-MeTHF (80 mL, 2V), and the contents stirred at 20 °C. The contents in Reactor 1 were then transferred to Reactor 2. Following complete transfer, a deionized H_2O (40 mL, 1V) rinse was added to Reactor 1 and transferred to Reactor 2. The contents of Reactor 2 were heated to 68 °C and stirred for 3 hours. Once the Suzuki reaction was complete, 3M KOH

(200 mL, 600 mmol, 6.0 eq) was added to Reactor 2 and stirred for 1 hour at the same temperature. Upon hydrolysis completion, the contents were cooled to 20 °C.

The contents were filtered through a celite plug (20 g) and returned to a clean reactor. Once the aqueous phase was separated, the organic phase was concentrated at 50 °C (NLT 100 torr), diluted with deionized H_2O (800 mL, 20V), and further concentrated until the volatile organics were removed (NLT 55 torr). The aqueous phase was pH-adjusted to pH ≥ 12.5 with aq. KOH (160 mL, 3 wt%) and washed thrice with MTBE (200mL, 5V each). The aqueous phase was then extracted once with MIBK (400 mL, 10V), and the organic phase was treated with SilaMetS Thiol (8.40 g, 10 wt% of theoretical yield) as a slurry at 80 °C for 3 hours. The contents were cooled to 20 °C, filtered through a celite plug (20 g), and rinsed with MIBK (200 mL, 5V). The filtrate is returned to a clean reactor, washed successively with 3M KOH (40 mL, 1V) and deionized H_2O (40 mL, 1V), concentrated at 50 °C (NLT 30 torr) to ~200 mL batch volume, and precipitated with heptane (400 mL, 10V) over NLT 10 minutes. The contents were cooled to 20 °C, filtered, and the solid was dried in a vacuum oven at 75 °C, 30 torr, for NLT 12 hours to yield **7-K** (65.05 g, 90.4 wt% by HPLC) as a light-yellow solid (69.7% corrected yield).

^{1}H NMR (600 MHz, DMSO-d_6) δ 7.74 – 7.67* (m, 2H), 7.41 – 7.26* (d, J = 6 Hz, 1H), 7.08 – 6.94* (m, 3H), 6.51 – 6.41* (m, 2H), 4.33 – 4.03* (m, 3H), 3.36 (s, 3H), 3.24 – 3.23* (m, 3H), 2.86 – 2.77* (m, 4H), 1.72 (brs, 6H), 1.26 – 1.21* (s, 9H). *Signals from minor atropisomer included.

^{13}C NMR (151 MHz, DMSO-d_6) δ 208.3, 162.8 (d, J = 13.6 Hz), 161.2 (d, J =13.6), 160.7, 155.5, 152.1, 143.2 – 143.1 (m), 140.9, 139.8, 139.3, 131.4, 129.9, 128.2, 126.3, 124.7, 122.8, 119.2, 117.6, 117.5, 111.9 – 111.8 (m), 102.0 – 101.6 (m), 87.5, 84.8, 78.2, 57.3, 55.5, 51.8, 35.1, 30.1, 28.1, 24.9, 23.9, 22.5, 22.3.

^{19}F NMR (565 MHz, DMSO-d_6) δ -68.3 (t, J = 8.7 Hz, 1F), -68.6 (m, 0.3H)*, -68.8 (t, J = 9.3 Hz, 3F), -110.4 – -110.5 (m, 2F), -110.6 – -110.7* (m, 0.5F), -110.8 – -110.9* (m, 0.2F), -111.0 – -111.1* (m, 0.2F). *Signals from minor atropisomer included.

A% (275 nm): 98.1%

Atropisomeric ratio based on A% (275 nm): major: 83.2% (retention time: 10.11min); minor atropisomer: 16.8% (retention time: 9.76 min)

HPLC wt% purity (275 nm): L1.3-Boc free acid (85.7 wt%)

GC-FID solvent analysis: MIBK (3.90 wt%); Heptane (0.13 wt%)

ICP-OES metals analysis: Pd (7.9 ppm)

KF water content analysis: H_2O (1.32 wt%)

ELSD salt content analysis: 4.7 wt%

LC-MS (m/z) $[M-K+H]^+$: 804

IR (ATR) νmax: 2978, 2935, 1703, 1625, 1595, 1577, 1508, 1482, 1366, 1305, 1228, 1198, 1157, 1103, 1042, 977, 841, 760

HRMS (ESI) m/z: calcd for $C_{34}H_{34}ClF_5KN_5O_6S_2 \cdot H^+ = [M+H]^+$ 842.1269, found 842.1264

Melting point: 210-215 °C.

Synthesis of 8 (Step 2: Boc-Deprotection of 7-K)

Synthesis of (S)-N-(7-(2-(1-amino-2-(3,5-difluorophenyl)ethyl)-6-(3-methyl-3-(methylsulfonyl)but-1-yn-1-yl)pyridin-3-yl)-4-chloro-1-(2,2,2-trifluoroethyl)-1H-indazol-3-yl)methanesulfonamide (8).

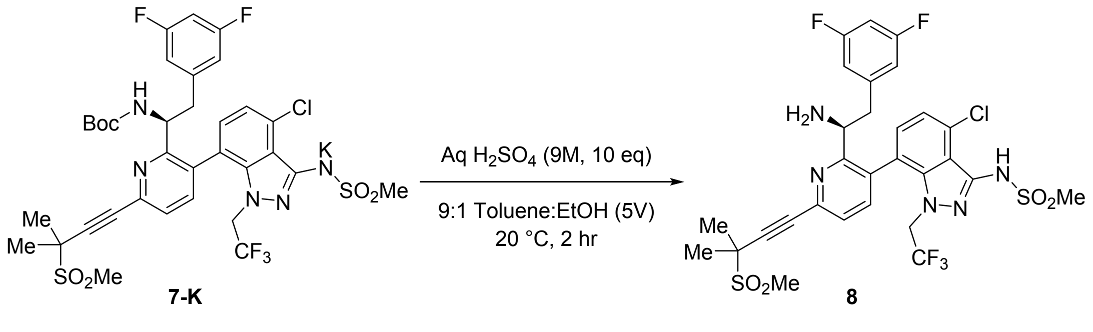

To a 500 mL ChemRxnHub reactor was added **7-K** (50.00g, 53.66 mmol, 1.0 eq, 90.4 wt%), toluene (225 mL, 4.5V), and EtOH (25 mL, 0.5V). The reaction was stirred for 20-30 min at 20

°C under N_2 atmosphere to obtain a clear solution. After which, 9M sulfuric acid (59.6 mL, 536.6 mmol, 10 eq) was added dropwise via syringe, and the reaction was stirred at 20 °C until Boc-deprotection was completed. After 2 hours, EtOAc (750 mL, 15V) and H_2O (75 mL, 1.5V) were added and stirred for 10 minutes. The layers were separated, and the organic layer was basified (pH ~8.5) using 3M NaOH (20 mL, 0.4V). The layers were separated, and the organic layer was washed two times with deionized H_2O (250 mL, 5V each). The final organic layer was concentrated at 55 °C (NLT 50 torr) to ~50 mL batch volume (1V). Heptane (500 mL, 10V) was added, and the slurry was stirred for 30 minutes. After which, the solid was collected via filtration using a disposable funnel, suction dried for 30 minutes and then dried in a vacuum oven at 75 °C, 30 torr, for NLT 12 hours to afford **8** (39 g, 94.1 wt% by HPLC) as a light-yellow solid (97 % corrected yield).

^{1}H NMR (600 MHz, DMSO-*d$_6$*) (**Major isomer**) δ: 7.74 (d, *J* = 8.0 Hz, 1H), 7.71 (d, *J* = 7.9 Hz, 1H), 7.21 (d, *J* = 7.6 Hz, 1H), 7.03 – 6.93 (m, 1H), 6.63 (d, *J* = 7.6 Hz, 1H), 6.31 (dd, *J* = 8.3, 2.1 Hz, 2H), 4.91 (td, *J* = 16.8, 8.5 Hz, 1H), 4.32 (td, *J* = 16.9, 8.5 Hz, 1H), 3.62 (t, *J* = 6.8 Hz, 1H), 3.25 (s, 3H), 3.16 (s, 3H), 2.91 – 2.74 (m, 2H), 1.73 (s, 6H).

^{1}H NMR (600 MHz, DMSO-*d$_6$*) (**Minor isomer**) δ: 7.82 (d, J = 8.0 Hz, 1H), 7.71 (d, J = 7.9 Hz, 1H), 7.42 (d, J = 7.7 Hz, 1H), 7.36 (d, J = 7.7 Hz, 1H), 7.02 – 6.93 (m, 1H), 6.60 (dd, J = 8.3, 2.0 Hz, 1H), 6.31 (dd, J = 8.3, 2.1 Hz, 1H), 4.50 (dt, J = 24.3, 8.3 Hz, 1H), 4.10 – 3.96 (m, 1H), 3.70 (dd, J = 8.6, 5.1 Hz, 1H), 3.25 (s, 3H), 3.16 (s, 3H), 2.98 (dd, J = 13.4, 8.7 Hz, 1H), 2.90 – 2.83 (m, 1H), 1.73 (s, 6H).

^{13}C NMR (151 MHz, DMSO-*d$_6$*) δ 163.3 (d, *J* = 13.3 Hz), 161.7 (d, *J* = 13.4 Hz), 161.1, 143.0, 142.2, 141.5 (d, *J* = 177.2 Hz), 140.1, 139.95 – 139.8 (m), 139.7, 131.1 (d, *J* = 106.3 Hz), 129.0 (d, *J* = 104.6 Hz), 124.6, 122.7, 122.4, 119.7, 118.2, 117.8, 112.4 (d, *J* = 24.3 Hz), 102.2 (t, *J* = 25.6 Hz), 88.6, 85.1, 57.8, 55.3, 50.7 (d, *J* = 33.5 Hz), 44.4, 41.6 (s), 41.4, 35.5 (d, *J* = 3.6 Hz), 31.7, 28.8, 22.8 – 22.7 (m), 22.6, 14.4. (Atropisomers cannot be differentiated in ^{13}CNMR).

^{19}F NMR (565 MHz, DMSO-*d$_6$*) (**Major isomer**) δ -68.95 (t, *J* = 8.6 Hz, 3F), -110.42 (t, *J* = 7.8 Hz, 2F).

^{19}F NMR (565 MHz, DMSO-*d$_6$*) (**Minor isomer**) δ -68.75 (t, *J* = 8.3 Hz, 3F), -110.42 (t, *J* = 7.8 Hz, 2F).

A% (275 nm): 96.8 %

Atropisomeric ratio based on A% (275 nm): major: 83.0% (retention time: 7.77 min); minor atropisomer: 17.0% (retention time: 6.14 min)

HPLC wt% purity (275 nm): 94.1 %

GC-FID solvent analysis: 2.49 % Heptane; 0.40 % Toluene

ICP-OES metals analysis: 18.2 ppm

KF water content analysis: 0.83 %

ELSD salt content analysis: Not detected

LC-MS (m/z) $[M+H]^+$: 704

IR (ATR) νmax: 3360, 3248, 3021, 2935, 1625, 1593, 1577, 1500, 1446, 1357, 1303, 1262, 1239, 1154, 1115, 1044, 973, 937, 885, 848, 829, 759

HRMS (ESI) m/z: calcd for $C_{29}H_{27}ClF_5N_5O_4S_2 \cdot H^+ = [M+H]^+$ 704.1186 , found 704.1183

Melting point: 126-127 °C.

Synthesis of Len-API-Na (Step 3: Amidation of 8 with Frag C)

Synthesis of Sodium (4-chloro-7-(2-((S)-1-(2-((3bS,4aR)-5,5-difluoro-3-(trifluoromethyl)-3b,4,4a,5-tetrahydro-1H-cyclopropa[3,4]cyclopenta[1,2-c]pyrazol-1-yl)acetamido)-2-(3,5-difluorophenyl)ethyl)-6-(3-methyl-3-(methylsulfonyl)but-1-yn-1-yl)pyridin-3-yl)-1-(2,2,2-trifluoroethyl)-1H-indazol-3-yl)(methylsulfonyl)amide (Len API-Na)

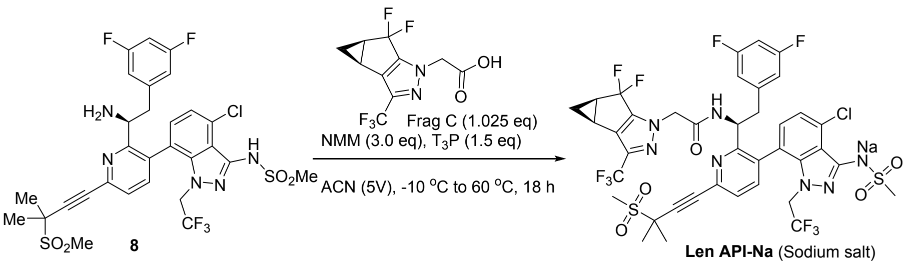

To a 1000 mL ChemRxnHub Reactor 1 was added **8** (35g, 1eq, 46.72 mmol, 94 wt%) and **Frag C** (13.5g, 1.025 eq, 47.89 mmol, 100 wt%). Reagent-grade acetonitrile (175mL, 5V) was added to Reactor 1, and the mixture is stirred at 25 °C under N_2. The mixture was then cooled to an internal

temperature of -10 °C. 4-Methylmorpholine (15.9mL, 3.0 eq, 140.2 mmol) was added slowly (8 mL/min) to Reactor 1, at -10 °C (internal) under N_2 and stirred for NLT 10 minutes, after which, a 50% solution of T3P (27.8mL, 1.0 eq, 46.72 mmol, 50 wt%) in DMF was added slowly (8 mL/min) to Reactor 1, at -10 °C under N_2. The reactor was then warmed to 25 °C and further heated to NLT 60 °C and stirred for NLT 4.5 hours. After the allotted time, a second lot of 50% T3P (8.2 mL, 0.3 eq, 14.0mmol, 50wt%) in DMF was added slowly (8 mL/min) to Reactor 1 at 60 °C under N_2. The reactor was maintained under these conditions for NLT 18 hours. After 18h, a third lot of 50% T3P (5.44 mL, 0.2 eq, 9.3mmol, 50wt%) in DMF was added slowly (8 mL/min) to Reactor 1 at 60 °C under N_2. The reactor was maintained under these conditions for NLT 1 hour.

The reaction mixture was basified (pH ~13.7) with 1M NaOH (383mL, 8 eq, 374mmol), then stirred and heated to 45 °C (internal) for NLT 3 hours. To this reaction mixture was added MIBK (358mL, 10V), and the mixture was stirred for NLT 15 minutes. A phase separation was undertaken, and the aqueous layer was discharged. The organics were concentrated via distillation (250 to 50 mbar) to 40mL, ~1V, and then heptane (530mL, 15V) was added, stirred for 1 hour. The resulting solid was filtered using a disposable fritted funnel and suction-dried for 1 hour. The solid was transferred to 2000 mL ChemRxnHub Reactor 2. To this, EtOH (530mL, 10V based on crude solid) was added and stirred at 70 °C for 15 min until a clear solution was observed. Then heptane (1060mL, 20V based on crude solid) was added dropwise over 3h maintaining temperature 70 °C. The mixture was stirred at 70 °C for 30 minutes, and then slowly cooled to room temperature over 16 hours. The solid was filtered with a disposable fritted funnel, washed with 1:2 EtOH:Heptane (530mL, 10V), suction dried for NLT 1h. The obtained solid was charged back to Reactor 2. To this, EtOH (600mL, 10V based on crude solid) was added and stirred at 70 °C for 15 min until a clear solution was observed. Then heptane (1200mL, 20V based on crude solid) was added dropwise over 3h maintaining temperature 70 °C. The mixture was stirred at 70 °C for 30 minutes, and then slowly cooled to room temperature over 16 hours. The solid was filtered with a disposable fritted funnel, washed with 1:2 EtOH:Heptane (600mL, 10V), suction dried for NLT 1h and finally oven dried at 75 °C under vacuum (30 torr) for NLT 12 h until constant weight to obtain pure **Len-API-Na** sodium salt as a yellow solid in (37.5 g, 78.4% corrected yield, 102.48wt%)

^{1}H NMR (600 MHz, DMSO-d_6) (**Major isomer**) δ: 9.08 (d, *J* = 8.0 Hz, 1H), 7.69 (s, 2H), 7.04 – 6.96 (m, 1H), 6.93 (d, *J* = 7.6 Hz, 1H), 6.67 (d, *J* = 7.6 Hz, 1H), 6.44 (d, *J* = 6.3 Hz, 2H), 4.89 (d, *J* = 16.5 Hz, 1H), 4.76 (d, *J* = 16.5 Hz, 1H), 4.69 (td, *J* = 8.4, 5.1 Hz, 1H), 4.19 (dq, *J* = 16.6, 8.3 Hz, 1H), 3.81 (dq, *J* = 17.6, 8.9 Hz, 1H), 3.25 (s, 3H), 2.93 (ddd, *J* = 22.5, 13.6, 7.0 Hz, 2H), 2.78 (s, 3H), 2.61 – 2.51 (m, 2H), 1.74 (s, 3H), 1.73 (s, 3H), 1.39 (dd, *J* = 13.4, 7.2 Hz, 1H), 0.94 (d, *J* = 3.3 Hz, 1H).

^{1}H NMR (600 MHz, DMSO-d_6) (**Minor isomer**) δ: 8.92 (d, *J* = 8.5 Hz, 1H), 7.75 (dd, *J* = 21.8, 7.9 Hz, 2H), 6.95 (d, *J* = 12.0 Hz, 1H), 6.91 – 6.85 (m, 2H), 6.52 (d, *J* = 6.3 Hz, 2H), 4.83 – 4.78 (m, 2H), 4.63 (d, *J* = 16.5 Hz, 1H), 4.28 (dd, *J* = 16.0, 8.2 Hz, 1H), 3.73 (dd, *J* = 16.5, 8.1 Hz, 1H), 3.24 (s, 3H), 3.09 – 3.00 (m, 2H), 2.83 – 2.79 (s, 3H), 2.62 – 2.51 (m, 2H), 1.73 (d, J = 3.6 Hz, 6H), 1.45 – 1.41 (m, 1H), 0.98 (s, 1H).

^{13}C NMR (151 MHz, CDCl3-*d*) δ 164.4, 163.4, 163.0, 162.9, 161.3, 161.2, 158.9, 158.2, 152.6, 151.9, 143.0 – 142.9 (m), 142.9 – 142.8 (m), 142.8 – 142.7 (m), 142.6, 142.4, 142.4 – 142.2 (m), 142.1 (d, *J* = 9.4 Hz), 141.4 (s), 141.1, 139.6 (s), 139.4, 134.0 (d, *J* = 38.6 Hz), 133.6, 133.2 – 133.0 (m), 132.1, 131.9, 131.7, 129.7, 129.6, 128.2, 127.9, 126.9, 126.8, 124.5, 123.5 – 123.1, 122.7, 121.6, 121.6 – 121.5 (m), 119.9, 119.9, 119.8, 118.9, 118.2 (d, *J* = 21.1 Hz), 117.5, 117.2, 117.0, 112.1, 111.97, 111.8, 102.2, 102.0, 101.8, 88.1, 88.0, 84.7, 57.3, 56.0, 53.2, 53.0, 52.8, 52.4, 49.5 (d, *J* = 32.1 Hz), 40.0, 38.6, 35.1, 27.7, 27.6, 27.3, 23.2, 22.3 (d, *J* = 18.7 Hz), 11.6. (Atropisomers cannot be differentiated in ^{13}CNMR).

^{19}F NMR (565 MHz, DMSO-d_6) (**Major isomer**) δ: -60.01 (s, 3F), -68.55 (s, 3F), -79.33 (d, *J* = 254.0 Hz, 1F), -102.61 (d, *J* = 253.9 Hz, 1F), -109.89 (s, 2F).

^{19}F NMR (565 MHz, DMSO-d_6) (**Minor isomer**) δ: -59.97 (s, 3F), -68.03 (s, 3F), -79.58 (d, *J* = 254.2 Hz, 1F), -102.58 (d, *J* = 253.7 Hz, 1F), -109.97 (s, 2F).

A% (235 nm): 98.8A%

Atropisomeric ratio based on A% (235 nm): major: 85.9% (retention time: 11.96 min); minor atropisomer: 14.1% (retention time: 11.42 min)

HPLC wt% purity (235 nm): 100.6%±2.8%

GC-FID solvent analysis: 0.3%±0.04% (EtOH); 0.06%±0.01% (Heptane)

ICP-OES metals analysis (Pd content): < 4.2 ppm

KF water content analysis: 0.3%±0.01%

ELSD salt content analysis: 1.88%±0.08%

LC-MS (m/z) $[M - Na + H]^+$: 968

IR (ATR) νmax: 3651, 3366, 3021, 2943, 1702, 1627, 1590, 1515, 1485, 1459, 1385, 1366, 1351, 1314, 1260, 1236, 1148, 1131, 1109, 1042, 1018, 978, 945, 842, 807, 762

HRMS (ESI) m/z: calcd for $C_{39}H_{31}ClF_{10}N_7NaO_5S_2 \cdot H^+ = [M+H]^+$ 990.1327, found 990.1326

Melting point: 238-240 °C.

$[\alpha]_D^{20}$ (deg·mL·g^{-1}·dm^{-1}) (MeOH (10mg/mL) at 20 °C under 589nm): -93.044

9. Synthesis and characterization of major impurities

N-(4-chloro-1-(2,2,2-trifluoroethyl)-1H-indazol-3-yl)methanesulfonamide (12)

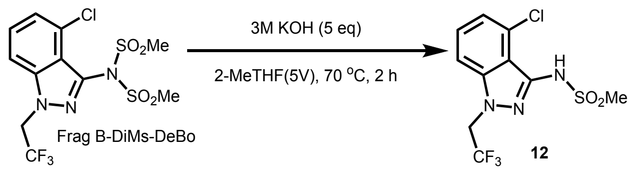

To a two-neck 100 mL round-bottom flask was added **Frag B-DiMs-DeBo** (1.5 g, 3.7 mmol, 1 eq), 2-MeTHF (7.5 mL, 5V) and KOH (3M, 6.2 mL, 18.5 mmol, 5 eq) at room temperature. The reaction mixture was heated at 70 °C for 2 h. Upon completion of the reaction (monitored by TLC), the mixture was cooled to 20 °C. The mixture was extracted twice with EtOAc (15 mL each, 10V). The organic layers were combined and washed with 5% acetic acid (7.5 mL, 5V), followed by deionized water (7.5 mL, 5V) to pH = 7.0. The organic layer was concentrated and treated with heptane (10.5 mL, 7 V). The slurry was filtered to afford **12** as a solid (0.70 g, 2.1 mmol, 57.8 % uncorrected yield).

^{1}H NMR (600 MHz, DMSO-d_6) δ 9.94 (s, 1H), 7.80 (d, J = 8.5 Hz, 1H), 7.55 – 7.41 (m, 1H), 7.29 (d, J = 7.4 Hz, 1H), 5.49 (q, J = 9.0 Hz, 2H), 3.22 (s, 3H).

^{13}C NMR (151 MHz, DMSO-d_6) δ 142.8, 138.7, 128.7, 125.3, 127.4 – 119.8 (q, 280Hz), 122.5, 116.7, 109.4 (s), 49.0 (q, J = 33.6 Hz), 41.4.

^{19}F NMR (565 MHz, DMSO-d_6) δ -69.58 (t, J = 9.1 Hz).

A% (275 nm): 98.2 %

LC-MS (m/z) $[M+H]^+$: 328

IR (ATR) νmax: 3274, 3224, 3021, 2968, 2939, 1740, 1612, 1572, 1526, 1500, 1451, 1429, 1388, 1347, 1299, 1261, 1246, 1183, 1155, 1090, 1060, 978, 941, 892, 855, 833, 782, 762, 738

HRMS (ESI) m/z: calcd for $C_{10}H_9ClF_3N_3O_2S{\cdot}Na^+$= $[M+Na]^+$ 349.9948, found 349.9997

Melting point: 166 -168 °C.

***tert*-butyl (S)-(1-(3,6-bis(3-methyl-3-(methylsulfonyl)but-1-yn-1-yl)pyridin-2-yl)-2-(3,5-difluorophenyl)ethyl)carbamate (11)**

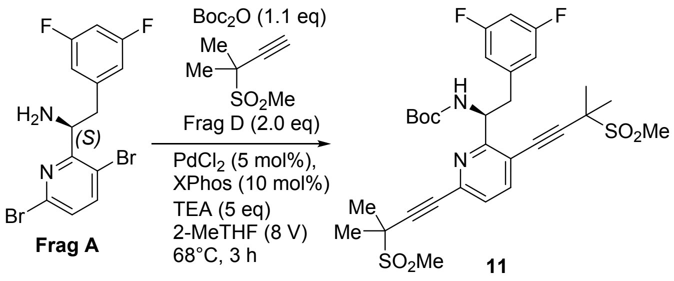

A 100 mL two-neck round-bottom flask was charged with **Frag A** (2.00 g, 5.1015 mmol, 1.0 eq), **Frag D** (1.49 g, 10.203 mmol, 2.0 eq), $PdCl_2$ (45.2 mg, 0.255 mmol, 0.05 eq), and XPhos (243.2 mg, 0.510 mmol, 0.1 eq). The above reaction mixture was degassed and backfilled with nitrogen three times. Then degassed 2-MeTHF (3.0 mL, 8V) was added. To the stirred mixture was slowly added a solution of di-tert-butyl dicarbonate (1.22 g, 5.6117 mmol, 1.1 eq) in degassed 2-MeTHF (2.0 mL, 1V), then degassed triethylamine (3.56 mL, 25.508 mmol, 5 eq) was added slowly. The reaction mixture was heated at 68 °C and stirred for 2 h under N_2 atmosphere. Upon completion of the reaction, 2-MeTHF was removed under vacuum at 50 °C. The solid was then suspended in

deionized H_2O (20 mL,10V) and stirred for 20 min. The solid was collected by filtration and washed with heptane (20 mL, 10 V). The solid was dried at 65 °C to afford **11** as a light brown solid (1.4 g, 2.1918 mmol, 42.9 % uncorrected yield).

^{1}H NMR (600 MHz, DMSO-*d$_6$*) δ 8.97 (bs, 1H), 7.90 (d, *J* = 7.8 Hz, 1H), 7.54 (d, *J* = 7.8 Hz, 1H), 7.42 (d, *J* = 7.8 Hz, 1H), 7.16 – 7.01 (m, 1H), 6.96 (d, *J* = 6.4 Hz, 1H), 5.22z (d, *J* = 6.5 Hz, 1H), 5.09* (s), 3.21 (s, 6H), 3.12* (d, *J* = 13.6 Hz), 3.05 (s, 1H), 2.98 (d, *J* = 9.1 Hz, 1H), 2.69 – 2.68 (m, 1H), 1.68 (d, *J* = 6.3 Hz, 12H), 1.57* (m), 1.28 (s, 9H), 1.16* (s).

^{13}C NMR (151 MHz, DMSO-*d$_6$*) δ 163.3, 161.6, 155.6, 143.1, 141.2, 140.8, 126.7, 117.0, 113.0, 102.3, 96.2, 89.4, 85.1, 81.6, 78.9, 78.5, 69.5, 58.0, 57.9, 57.78, 55.1, 46.2, 35.7, 35.5 (d, *J* = 3.8 Hz), 28.6, 28.3 – 28.0 (m), 22.8 (d, *J* = 4.8 Hz), 22.7, 22.5.

^{19}F NMR (565 MHz, DMSO-*d$_6$*) δ -110.7*, -110.8 (d, *J* = 7.8 Hz).

A% (275 nm): 98.6 %

LC-MS (m/z) $[M+H]^+$: 623

IR (ATR) νmax: 3371, 2982, 2935, 1707, 1627, 1597, 1498, 1448, 1366, 1299, 1163, 1113, 1012, 949, 844, 762

HRMS (ESI) m/z: calcd for $C_{30}H_{36}F_2N_2O_6S_2 \cdot H^+$= $[M+H]^+$ 623.2056, found 623.2055

Melting point: 121-123 °C.

N-(4-chloro-7-(2-((S)-1-(2-((3bS,4aR)-5,5-difluoro-3-(trifluoromethyl)-3b,4,4a,5-tetrahydro-1H-cyclopropa[3,4]cyclopenta[1,2-c]pyrazol-1-yl)acetamido)-2-(3,5-difluorophenyl)ethyl)-6-(3-methyl-3-(methylsulfonyl)but-1-yn-1-yl)pyridin-3-yl)-1-(2,2,2-trifluoroethyl)-1H-indazol-3-yl)-2-((3bS,4aR)-5,5-difluoro-3-(trifluoromethyl)-3b,4,4a,5-tetrahydro-1H-cyclopropa[3,4]cyclopenta[1,2-c]pyrazol-1-yl)-N-(methylsulfonyl)acetamide (13)

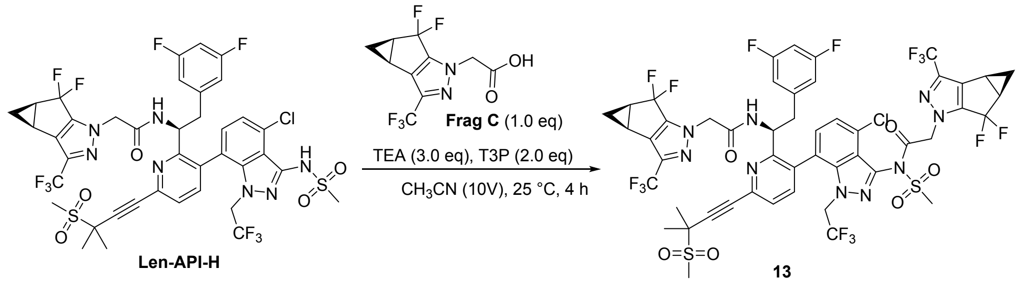

A 50 mL round-bottom flask was charged with **Len API-H** (500 mg, 0.51 mmol, 1.0 eq), **Frag C** (145.7 mg, 0.516 mmol, 1.0 eq), and 5.0 mL of CH_3CN (10V) under N_2 atmosphere. The mixture was stirred at 20 °C, and then TEA (0.22 mL, 1.55 mmol, 3.0 eq) was added slowly under N_2. The mixture was stirred for 5 minutes and then a solution of T3P in DMF (50 wt%, 0.6 mL 1.03 mmol, 2.0 eq) was added slowly. The mixture was stirred for another 4 h. After completion, 10 mL of ice-cold H_2O was added, and the mixture was stirred for 10 minutes. The mixture was extracted twice with EtOAc (30 mL each). The organic layers were combined and washed with brine (20 mL). The EtOAc layer was separated and rotovapped to dryness. The residue was precipitated with heptane (5 mL, 10 V). The solid was collected by filtration and dried under vacuum at 60 °C for 7 h to afford 0.37 g of **13** as a light-yellow solid (58.1% uncorrected yield).

^1H NMR (600 MHz, DMSO-d_6) (**Major isomer**) δ: 9.10 (d, J= 8.5Hz, 1H), 7.81 (d, J = 7.9 Hz, 1H), 7.48 (dd, J = 15.6, 5.7 Hz, 1H), 6.93 (t, J = 9.2 Hz, 1H), 6.89 (d, J = 7.7 Hz, 1H), 6.54 – 6.45 (m, 1H), 6.39 (d, J = 6.5 Hz, 2H), 4.98 – 4.83 (m, 2H), 4.81 – 4.53 (m, 3H), 4.03 (m, 1H), 3.63 (s, 3H), 3.26 (s, 3H), 3.02 (m, 2H), 2.73 – 2.55 (m, 2H), 2.43 (dd, J = 22.4, 9.8 Hz, 2H), 1.75 (s, 6H), 1.41 (ddd, J = 24.4, 13.6, 6.7 Hz, 2H), 1.30 – 1.19 (m, 1H), 0.97 (s, 2H).

^1H NMR (600 MHz, DMSO-d_6) (**Minor isomer**) δ: 9.21 (t, J = 10.1 Hz, 1H), 7.92 (d, J = 7.9 Hz, 1H), 7.76 (dd, J = 20.7, 8.0 Hz, 1H), 7.54 – 7.44 (m, 1H), 7.00 (dd, J = 17.7, 8.4 Hz, 1H), 6.99 – 6.95 (m, 1H), 6.54 – 6.44 (m, 2H), 4.86 (ddd, J = 32.8, 22.0, 12.7 Hz, 2H), 4.80 – 4.51 (m, 3H), 4.22 – 4.05 (m, 1H), 3.63 (s, 3H), 3.26 (s, 3H), 3.00 (m,2H), 2.72 – 2.56 (m, 2H), 2.43 (dd, J = 22.4, 9.8 Hz, 2H), 1.75 (s, 6H), 1.41 (ddd, J = 24.4, 13.6, 6.7 Hz, 2H), 1.21 (m, 1H), 1.01 (d, J = 14.1 Hz, 2H).

^{13}C NMR (151 MHz, , DMSO-d_6) δ 170.78, 166.56, 166.07, 165.08, 164.90, 163.42, 163.39, 162.75, 161.79, 161.70, 159.29, 158.84, 143.13, 143.02, 142.76, 142.10, 140.76, 140.72, 139.76, 139.45, 135.36, 133.10, 132.25, 130.12, 127.51, 125.858, 125.29, 124.10, 123.96, 122.24, 122.01,

121.92, 120.50, 120.38, 120.30, 120.14, 119.10, 118.54, 112.54, 112.37, 112.20, 102.62, 102.45, 89.05, 88.86, 84.93, 60.19, 57.88, 57.81, 87.79, 54.29, 53.82, 53.43, 53.18, 52.32, 51.13, 50.90, 42.04, 41.86, 36.20, 35.63, 35.55, 35.52, 31.18, 28.01, 27.82, 23.63, 23.43, 22.83, 22.79, 22.76, 22.72, 22.49, 21.1514.48, 12.12. (Atropisomers cannot be differentiated in ^{13}CNMR.)

^{19}F NMR (565 MHz, DMSO-d_6) δ: -59.35 – -61.26 (m), -68.08 – -70.14 (m), -78.66 – -81.57 (m), -101.67 – -104.58 (m), -109.71 – -111.06 (m). (Atropisomers cannot be differentiated in ^{19}FNMR.)

LC-MS (m/z) $[M+H]^+$: 1232

Atropisomeric ratio based on A% (235 nm): major: 91.5% (retention time: 17.45 min); minor atropisomer: 8.5% (retention time: 17.37 min)

HRMS (ESI) m/z: calcd for $C_{49}H_{37}ClF_{15}N_9O_6S_2 \cdot Na^+ = [M+Na]^+$ 1254.1649, found 1254.1644

Melting point: 97-106 °C.

(S)-N-(1-(3-(4-chloro-3-(methylsulfonamido)-1-(2,2,2-trifluoroethyl)-1H-indazol-7-yl)-6-(3-methyl-3-(methylsulfonyl)but-1-yn-1-yl)pyridin-2-yl)-2-(3,5-difluorophenyl)ethyl)acetamid (14)

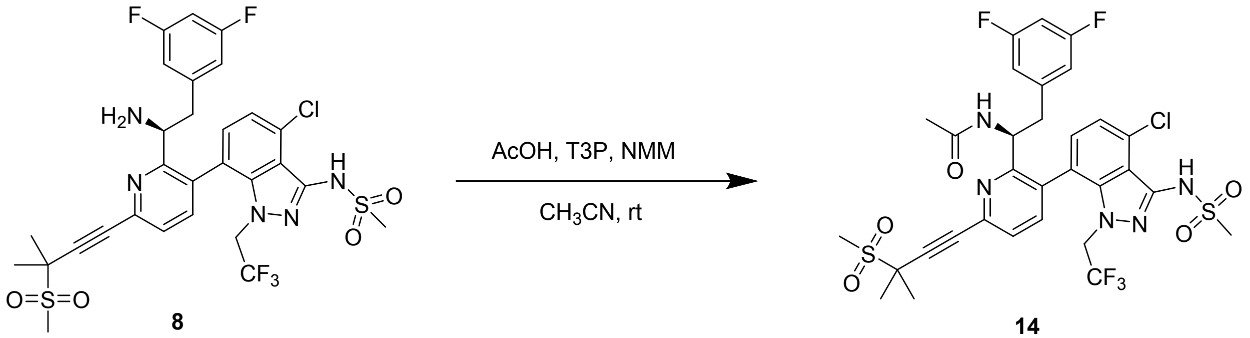

To a two-neck 50 mL round-bottom flask was added **8** (500 mg, 0.7 mmol, 1.0 eq), followed by acetic acid (0.06 mL, 1.04 mmol, 1.50 eq) to this was added acetonitrile (2.5 mL, 5V) at room temperature under N_2. N-Methylmorpholine (0.23 mL, 2.1 mmol, 3.0 eq, 2.1 mmol) was added slowly over a period of 5 minutes at -12 °C under N_2 and stirred for 30 seconds. Then, 50 % T3P (0.5 mL, 0.87 mmol, 1.25 eq) in DMF was added, and the reaction was allowed to stir at RT for 1

h under N_2. Upon completion, monitored via HPLC-UV, the reaction was distilled out completely, and diluted with MIBK (5.0 mL, 10V) and 1M NaOH (4.4 mL, 13.2 mmol, 3M, 19 eq) was added to adjust the pH to between 7-8. The organic layer was separated, distilled out completely, and subject to purification *via* SiO_2 column chromatography (0-80 % EtOAc in heptanes), to afford **14** as a semi-solid (0.25 g, 0.325 mmol, 46.8 % uncorrected yield).

^{1}H NMR (600 MHz, DMSO-d_6) δ 10.09 (d, *J* = 39.0 Hz, 1H), 8.67 (d, *J* = 7.9 Hz, 1H), 8.35* (d, *J* = 8.2 Hz), 7.82 (dd, *J* = 15.8, 8.0 Hz, 1H), 7.74 (dd, *J* = 7.9, 4.3 Hz, 1H), 7.39 (dd, *J* = 12.1, 7.7 Hz, 1H), 7.28* (d, *J* = 7.7 Hz), 7.13 (d, *J* = 7.6 Hz, 1H), 7.05 – 6.92 (m, 1H), 6.56* (d, *J* = 6.2 Hz), 6.49 (dd, *J* = 8.3, 2.1 Hz, 2H), 4.81 (dq, *J* = 16.6, 8.4 Hz, 1H), 4.76 – 4.67* (m), 4.47 (ddd, *J* = 9.5, 8.0, 4.7 Hz, 1H), 4.17 – 4.06 (m, 1H), 3.27 – 3.23 (s, 3H), 3.21 (s, 3H), 2.89 (ddd, *J* = 23.1, 13.6, 7.0 Hz, 1H), 1.73 (d, *J* = 2.5 Hz, 6H), 1.68 (s, 3h), 1.59* (s).

^{13}C NMR (151 MHz, DMSO-d_6) δ 169.0, 168.2, 162.8 (d, *J* = 13.3 Hz), 161.2 (d, *J* = 13.3 Hz), 160.1 (s), 142.7, 141.8, 139.6, 139.3, 139.2, 131.2, 130.2, 126.3 (t, *J* = 27.7 Hz), 125.1 (d, *J* = 302.5 Hz), 122.2, 119.4, 117.4, 111.9 (d, *J* = 24.4 Hz), 102.0 (t, *J* = 25.7 Hz), 88.2, 84.6, 57.3, 53.0, 52.6, 50.5 (dd, *J* = 65.6, 34.4 Hz), 41.4, 35.1 (d, *J* = 5.2 Hz), 22.3 (t, *J* = 9.3 Hz), 22.0.

^{19}F NMR (565 MHz, DMSO-d_6) δ -68.78 (t, *J* = 9.2 Hz), -110.32 (d, *J* = 9.9 Hz).

A% (275 nm): 95.5 %

Atropisomeric ratio based on A% (275 nm): major: 83.2% (retention time: 6.16 min); minor atropisomer: 16.8% (retention time: 4.85 min)

LC-MS (m/z) $[M+H]^+$: 746

IR (ATR) νmax: 3358, 3028, 3000, 2939, 1736, 1654, 1627, 1595, 1576, 1500, 1446, 1373, 1300, 1261, 1239, 1153, 1112, 1041, 974, 851, 829, 758

HRMS (ESI) m/z: calcd for $C_{31}H_{29}ClF_5N_5O_5S_2{\cdot}H^+$= $[M+H]^+$ 746.1292, found 746.1292

Melting point: 117-121 °C.

10. Process safety assessment

Process safety assessment by EasyMax- detailed procedure for calorimetry data collection

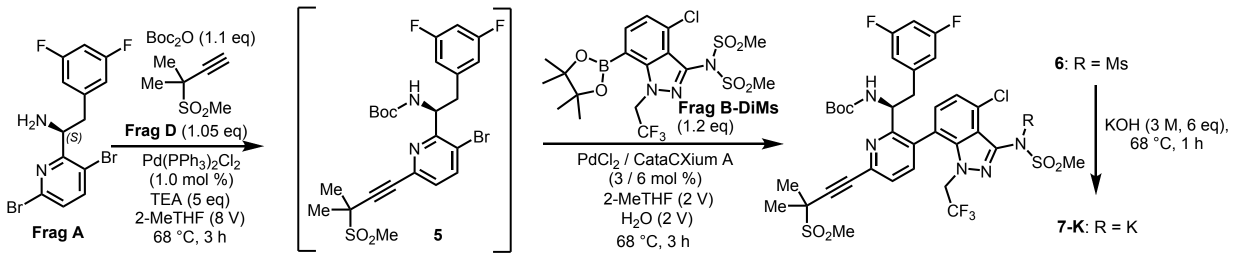

Synthesis of potassium (S)-(7-(2-(1-((tert-butoxycarbonyl)amino)-2-(3,5-difluorophenyl)ethyl)-6-(3-methyl-3-(methylsulfonyl)but-1-yn-1-yl)pyridin-3-yl)-4-chloro-1-(2,2,2-trifluoroethyl)-1H-indazol-3-yl)(methylsulfonyl)amide (7-K)

To a 100 mL glass reactor was added **Frag A** (5.0 g, 12.7 mmol, 1.0 eq), 2.06 g of **Frag D** (13.2 mmol, 94 wt%, 1.04 eq), 53.7 mg of bis(triphenylphosphine)palladium (II) dichloride (76.5 µmol, 0.6 mol%), and 7V of 2-MeTHF (35.0 mL). The vessel was evacuated via a vacuum pump and filled with N_2 four times. A baseline HFCal determination was performed (Quick Cal 1) for 1 hour and 10 minutes. Then, Boc anhydride (3.06 g, 14.0 mmol, 1.1 eq) in 1V of degassed 2-MeTHF (5.0 mL), was added dropwise via syringe at RT. Next, 8.9 mL of degassed triethylamine (63.7 mmol, 5 eq), was added dropwise via syringe at RT. The reaction mixture was heated to 68-70 °C (T_j = 72 °C) and stirred for 15 minutes. After which, an HFCal determination was performed (Quick Cal 2) for 1 hour and 10 minutes. The reaction was analyzed via TLC for completion and then transferred to the Suzuki reaction (reactor 2) as detailed below.

While the Heck coupling was running (reactor 1), a second 100 mL reaction vessel (reactor 2) was filled with 8.14 g of **Frag B-DiMs** (15.3 mmol, 1.2 eq), 67.8 mg of palladium (II) chloride (382.6 µmol, 3 mol%), and 274.4 mg of di((3S,5S,7S)-adamantan-1-yl)(butyl)phosphane (765.2 µmol, 6 mol%). The vessel was evacuated via a vacuum pump and filled with N_2 four times, then the Heck reaction mixture (reactor 1) was charged via syringe and 22G needle. The reaction mixture was heated to 68-70 °C (T_j = 72 °C) and stirred for 10 minutes. A baseline HFCal determination was performed (Quick Cal 1) for 1 hour and 10 minutes. After which, 10 mL each of deionized H_2O and 2-MeTHF were added, and the mixture was stirred for 10 minutes. An HFCal determination was performed (Quick Cal 2) for 1 hour and 10 minutes. The reaction was then analyzed via TLC for completion. Upon Suzuki reaction completion, an HFCal determination was performed (Quick Cal 1) for 1 hour and 10 minutes, then 21.256 mL of aqueous 3M potassium hydroxide (63.7 mmol, 5.0 eq) was added to the hot mixture and stirred for 20 minutes. Another HFCal determination was

performed (Quick Cal 2) for 1 hour and 10 minutes, and the reaction was analyzed via TLC for completion, then cooled to RT (T_j = 25 °C) and ceased.

Table S2 Heat evolution during the dosing steps of the Heck coupling.

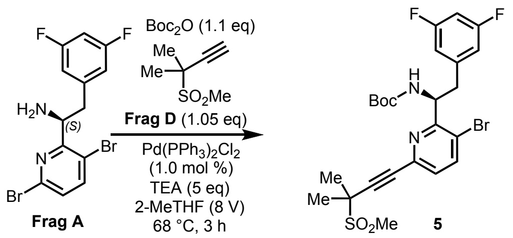

Process Step	ΔH_r (kJ)	ΔH_r (kJ/g)	ΔH_r (kJ/mol)	ΔT_{ad} (°C)	MTSR (°C)	MRT (°C)	MHG (W)	C_p (J/g K)		U (W/m^2K)	
								Before	After	Before	After
1[a]	-0.204	-0.041	-16.0	-3.27	68.7	69.0	8.36	3.36	1.73	178.8	139.6

[a]Addition of Boc anhydride and triethylamine to the reaction mass at 23 °C, C_p = Specific heat; U = Overall heat transfer coefficient.

Table S3 Heat evolution during the dosing steps of the Suzuki coupling and demesylation.

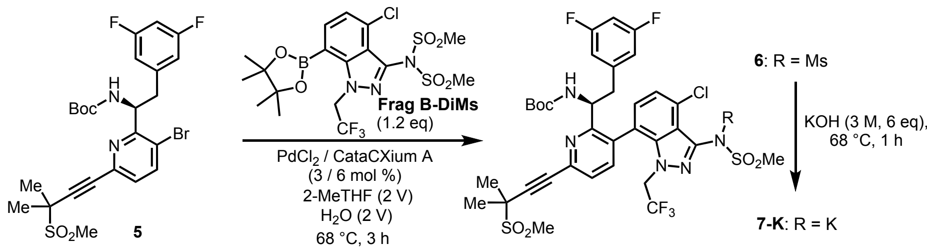

Process Step	ΔH_r (kJ)	ΔH_r (kJ/g)	ΔH_r (kJ/mol)	ΔT_{ad} (°C)	MTSR (°C)	MRT (°C)	MHG (W)	C_p (J/g K)		U (W/m^2K)	
								Before	After	Before	After
1[a]	-2.09	-0.298	-163.9	-129.7	-57.7	69.0	-2.92	-0.0475	0.250	207.0	179.2
2[b]	-15.5	-1.51	-1217	-7.54	64.5	69.0	-25.3	4.99	27.1	132.4	195.4

[a]Addition of deionized H_2O/2-MeTHF to the reaction mass at 72 °C; [b]Addition of KOH to the reaction mass at 72 °C.

Synthesis of (S)-N-(7-(2-(1-amino-2-(3,5-difluorophenyl)ethyl)-6-(3-methyl-3-(methylsulfonyl)but-1-yn-1-yl)pyridin-3-yl)-4-chloro-1-(2,2,2-trifluoroethyl)-1H-indazol-3-yl)methanesulfonamide (8)

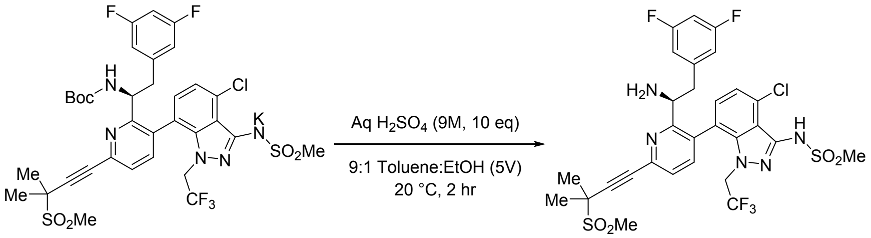

7-K **8**

To a 100 mL glass reactor was added of **7-K** (10.7 g, 12.7 mmol, 1 eq) and 5V of methyl ethyl ketone (53.7 mL) or **7-K** (10.0 g, 11.87 mmol, 1 eq) and 7V of 5:2 toluene:EtOH (70.0 mL) under N_2 at 20 °C (T_j = 20 °C). The mixture was stirred for 10 minutes, and then a HFCal determination was performed (Quick Cal 1) for 1 hour and 10 minutes. After which, 14.1 mL of aqueous 9M sulfuric acid (127.5 mmol, 10 eq) was added dropwise over various rates (i.e., 2, 12, and 45 minutes) and stirred for 10 minutes under N_2 at 20 °C (T_j = 20 °C). A HFCal determination was performed (Quick Cal 2) for 1 hour and 10 minutes, and the reaction was analyzed for completion via TLC, and then ceased.

Table S4 Heat evolution during the dosing step of the Boc deprotection.

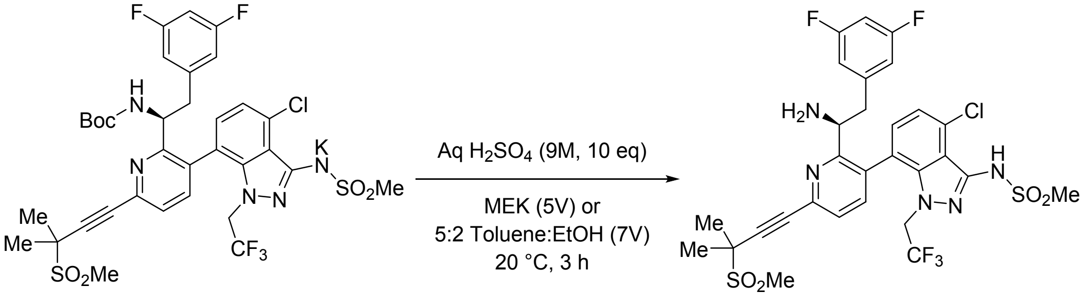

7-K **8**

Process Step	ΔH_r (kJ)	ΔH_r (kJ/g)	ΔH_r (kJ/mol)	ΔT_{ad} (°C)	MTSR (°C)	MRT (°C)	MHG (W)	C_p (J/g K)		U (W/m^2K)	
								Before	After	Before	After
1[a]	2.27	0.211	178.1	13.7	33.7	31.1	47.3	2.10	1.94	139.9	117.0
2[b]	3.34	0.311	261.7	20.1	40.1	25.4	15.8	2.10	2.00	145.9	103.1
3[c]	1.3	0.13	113.6	14.	34.7	23.0	2.39	2.43	1.26	126.3	56.0

	5	5		7							

[a]MEK (5V) was used as a solvent, aq 9M H_2SO_4 was added to **7-K** (10.7g) in 2 minutes to the reaction mass at 23 °C; [b]MEK (5V) was used as a solvent, aq 9M H_2SO_4 was added to **7-K** (10.7g) in 12 minutes to the reaction mass at 23 °C; [c]Toluene/EtOH (5/2, 7V) was used as solvents, aq 9M H_2SO_4 was added to **7-K** (10g) in 45 minutes to the reaction mass at 23 °C.

Synthesis of Sodium (4-chloro-7-(2-((S)-1-(2-((3bS,4aR)-5,5-difluoro-3-(trifluoromethyl)-3b,4,4a,5-tetrahydro-1H-cyclopropa[3,4]cyclopenta[1,2-c]pyrazol-1-yl)acetamido)-2-(3,5-difluorophenyl)ethyl)-6-(3-methyl-3-(methylsulfonyl)but-1-yn-1-yl)pyridin-3-yl)-1-(2,2,2-trifluoroethyl)-1H-indazol-3-yl)(methylsulfonyl)amide (Len-API-Na)

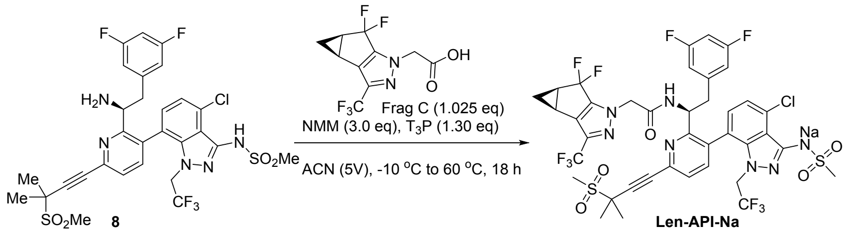

To a 100 mL glass reactor was added **8** (10.0 g, 14.2 mmol, 1 eq), 4.1 g of **Frag C** (14.5 mmol, 1.025 eq), and 5V of acetonitrile (50.0 mL) under N_2 at 20 °C (T_j = 20 °C) and stirred for 10 minutes. The reaction mixture was cooled to -10 °C (T_j = -13 °C) and stirred for 10 minutes. After which, a HFCal determination was performed (Quick Cal 1) for 1 hour and 10 minutes. Then, 4.6844 mL of NMM (42.6 mmol, 3.0 eq) was dosed over 2 minutes and then stirred for 10 minutes at -10 °C (T_j = -13 °C). Next, 8.2611 mL of T3P (14.2 mmol, 1.0 eq) was dosed over 2 minutes at -10 °C (T_j = -13 °C). The reaction was then heated to 60 °C (T_j = 65 °C) and stirred for 10 minutes. A HFCal determination was performed (Quick Cal 2) for 1 hour and 10 minutes, and then the reaction was held at 60 °C (T_j = 65 °C) for 2 hours. After the allotted time a HFCal determination was performed (Quick Cal 1) for 1 hour and 10 minutes, and then 2.5 mL of T3P (4.26 mmol, 0.3 eq) was dosed over 0.5 minutes at 60 °C (T_j = 65 °C). A final HFCal determination was performed (Quick Cal 2) for 1 hour and 10 minutes, and the reaction was held at 60 °C (T_j = 65 °C) for 15 hours. After which, the reaction was analyzed for completion via HPLC, cooled to RT (T_j = 20 °C), and then ceased.

Table S5 Heat evolution during the dosing steps of the **8** amidation.

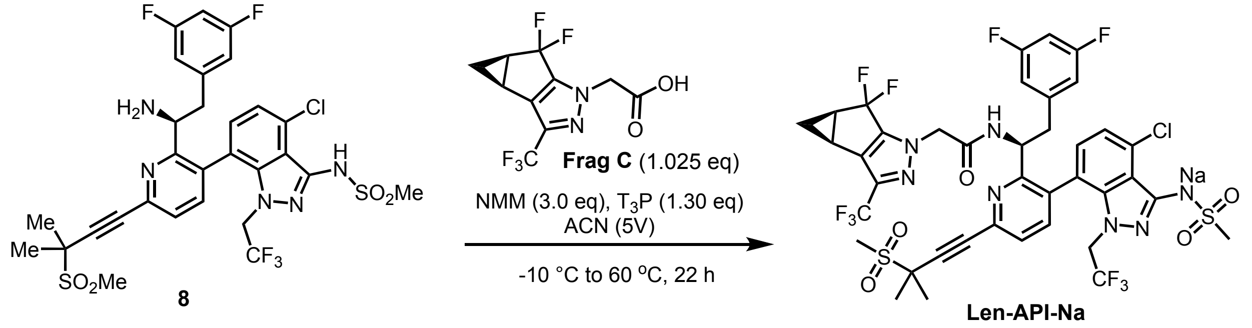

Process Step	ΔH_r (kJ)	ΔH_r (kJ/g)	ΔH_r (kJ/mol)	ΔT_{ad} (°C)	MTSR (°C)	MRT (°C)	MHG (W)	C_p (J/g K)		U (W/m^2K)	
								Before	After	Before	After
1[a]	0.075	0.0075	0.528	3.01	-9.99	-10.0	4.01	1.66	2.00	13.6	205.9
2[b]	2.73	0.0273	192.3	17.6	4.6	1.15	36.0	2.00	2.70	205.9	192.1
3[c]	0.343	0.0343	24.2	0.99	61.0	61.0	8.04	2.70	2.13	152.6	145.5

[a]Addition of NMM to the reaction mass at -10 °C; [b]Addition of T3P to the reaction mass at -10 °C; [c]Addition of T3P to the reaction mass at 60 °C.

11. Analytical Method Development

11.1 Analytical method development of 7-K synthesis

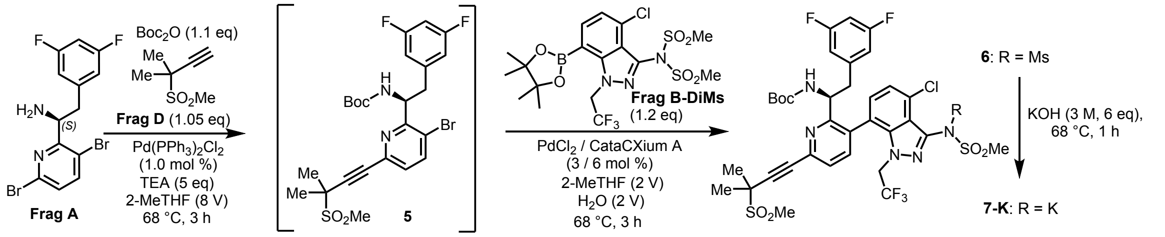

11.1.1 Compound 7-K In-Process and Assay Analysis

Chromatographic analysis was performed on an Agilent 1100/1200 liquid chromatograph equipped with a diode array detector (DAD). Separation was achieved using an Agilent ZORBAX Eclipse XDB-C18 column (2.1×150 mm, 3.5 µm) maintained at a temperature of 30°C. The mobile phase consisted of a binary gradient elution using mobile phases of 0.1% phosphoric acid in water (A) and acetonitrile (B) at a constant flow rate of 0.7 mL/min. Initial conditions were set to 45% B, ramping to 65% B over 12.00 min with a 2 min hold followed by a final ramp to 95%

B over 2 min with a 4 min hold. A post-run equilibration of 4 minutes was applied. Samples were prepared at 1 mg/mL in acetonitrile. An injection volume of 1.0 µL was used. The detection was monitored at 275 nm. The full method can be found in Appendix 3.1 (LCUV_Len API_Gradient). Figure S3 is a representative chromatogram for the various intermediates and impurities analyzed in the current method. It should be noted that **7** exists as atropisomers in solution and is observed as a pair of peaks on the chromatogram.

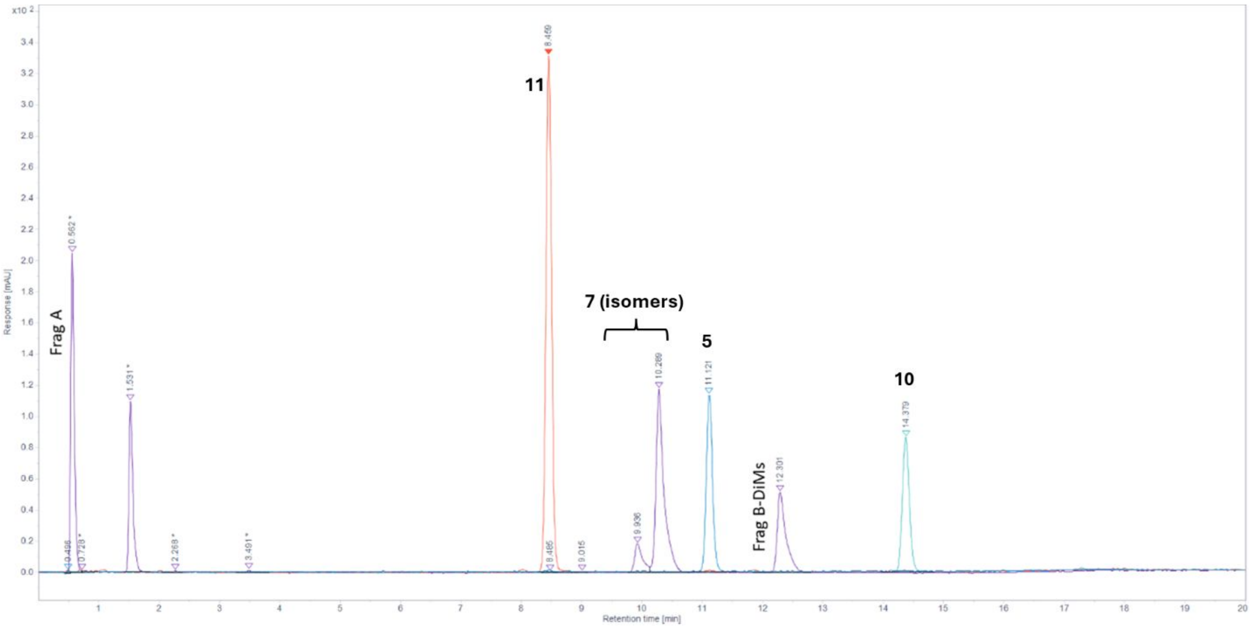

Figure S3 Representative chromatogram for **7-K** synthesis.

11.1.2 Relative Response Factors for 7-K Synthesis

Samples of **7**, impurities and synthetic intermediates were prepared at known concentrations and injected using the above method with a range of detection wavelengths to select an optimal wavelength for purity evaluation and reaction monitoring Figure S4. No true isosbestic point exists for this suite of analytes. As such, 275 nm was chosen for quantitation and monitoring purposes.

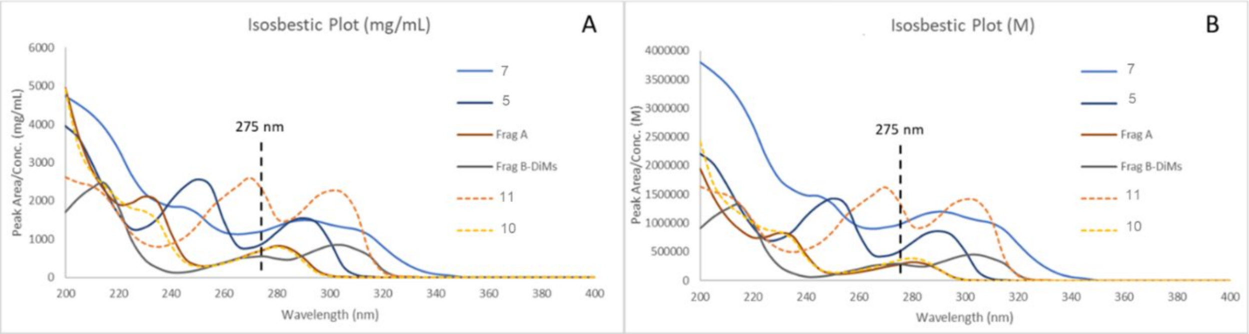

Figure S4 A) Isosbestic plot of **7** and associated analytes based on mg/mL. B) Isosbestic plot of **7** and associated analytes based on molarity (M).

Relative response factors (RRFs) were subsequently determined for starting materials and each available known impurity. Impurities were synthesized and purified in-house (vide supra). Samples were prepared at 1 mg/mL in acetonitrile and relative response factors were calculated using Equation S1. Table S6 lists the approximate retention times and RRFs (based on mg/mL and M basis) for each analyte.

$$\mathrm{RRF} = \frac{\left(\mathrm{Area}/\mathrm{Concentration}\right)_{\mathrm{Analyte}}}{\left(\mathrm{Area}/\mathrm{Concentration}\right)_{\mathrm{L1.3-Boc}}} \qquad \text{Eqn. S1}$$

Table S6. Relative response factors for Milestone 1 are calculated based on concentration (mg/mL)

Compound	Retention Time (min)	RRF at 275 nm (mg/mL)	RRF at 275 nm (M)
Frag A	0.56	0.54	0.26
**11**	8.46	1.83	1.42
**7**	9.94 / 10.28[a]	1.00	1.00
**5**	11.12	0.75	0.52
**Frag B-DiMs**	12.3	0.41	0.27
**6**	12.09 / 12.67[a]	-[b]	-[b]
**10**	14.38	0.60	0.37

[a]Isomers, all RRFs were calculated using the sum of the isomer peak areas. [b]Sufficient quantity not available for determination

11.1.3 Compound 7-K Potassium Analysis

Potassium salt analysis was performed using a Hydrophilic Interaction Liquid Chromatography (HILIC) method with an Agilent InfinityLab Poroshell 120 HILIC-Z column (3.0×150 mm, 2.7 μm). The mobile phases were: 10 mM ammonium acetate, pH 4 (Mobile Phase A) and 90:10 acetonitrile: 10 mM ammonium acetate, pH 4 (Mobile Phase B). A gradient elution was employed, starting at 90% B, transitioning to 80% B over 5 minutes, and then to 20% B over 5 minutes, which was held for an additional 4 minutes, followed by a 3-minute post-run equilibration period. The flow rate was maintained at 0.8 mL/min, and the column temperature was set to 30° C. A sample injection volume of 1.5 μL was used. Detection was achieved with an evaporative light scattering detector (ELSD). Samples were prepared at a concentration of 8 mg/mL in acetonitrile.

For ELSD detection, the following parameters were applied: evaporator temperature of 30° C, nebulizer temperature of 30° C, and gas flow of 1.6 L/min. The detector operated with a data rate of 80 Hz, a LED intensity of 100%, smoothing set to 30, and a PMT gain of 1.00. Figure S5 is a representative chromatogram for **7-K**.

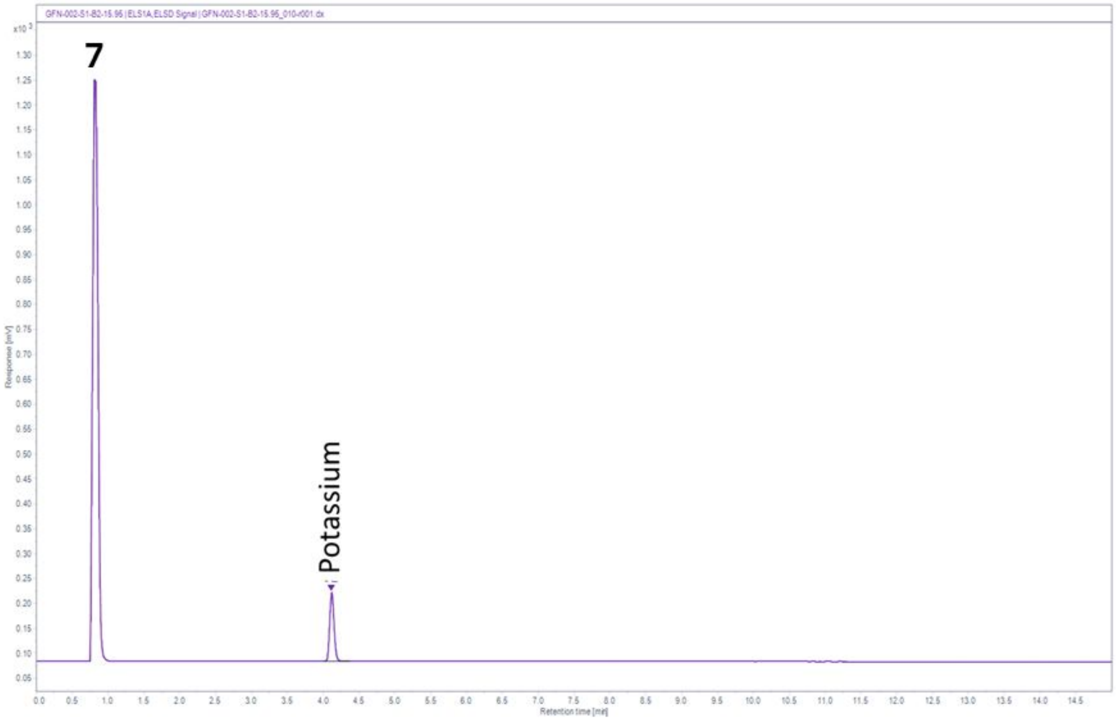

Figure S5. Representative chromatogram for **7-K** using the HILIC method for salt analysis.

11.1.4 Compound 7-K Solvent Analysis

Solvents were analyzed by gas chromatography (GC) - flame ionization detector (FID) with a HP-1 column (30 m × 320 µm, 5 µm film thickness). Inlet pressure was 4.8 psi, and the injection temperature was set to 260° C. A split injection was used with a split ratio of 50:1. The column flow was 0.787 mL/min. The temperature program started at an initial temperature of 50° C, held for 5 minutes, and then ramped at a rate of 20° C/min to a final temperature of 235° C, which was held for 5.75 minutes. The detector was set to 200° C. The detector gas flows were as follows: air flow at 450 mL/min, H_2 fuel flow at 35 mL/min, and N_2 makeup flow at 30 mL/min. Samples were prepared at a concentration of 5-10 mg/mL in acetonitrile or methanol.

11.1.5 Compound 7-K Palladium Elemental Analysis

Inductively Coupled Plasma-Optical Emission Spectrometry (ICP-OES) was utilized for the analysis of palladium (Pd) using an Agilent 5800 instrument. The operating conditions were as follows: a Radio Frequency (RF) Power of 1.4 kW was applied to generate a robust plasma with a plasma gas flow rate of 12.0 L/min. The auxiliary gas flow was set to 1.0 L/min, and the nebulizer gas flow was 0.70 L/min, with a pressure of 220 kPa. The sample introduction system featured a glass cyclonic spray chamber and a SeaSpray nebulizer, with an uptake rate of 0.8 mL/min. The analysis was conducted in SVDV (Synchronous Vertical Dual View) mode, which provided both axial and radial views for simultaneous detection of low and high concentrations. Palladium was monitored at the 340.458 nm emission line with a replicate read time of 5 seconds, a stabilization time of 15 seconds, and a sample uptake delay time of 10 seconds.

Samples were prepared with a minimum of 50 mg of sample which was dissolved in 5 mL of 10% HCl in methanol. The mixture was vortexed and/or sonicated to ensure complete dissolution. A 1 mL aliquot of this solution was then diluted to a final volume of 10 mL with a 10% HCl in deionized water matrix. For samples with residual solids, the final solution was filtered using a 0.45µm PTFE syringe filter.

11.1.6 Compound 7-K LC-MS Analysis

Separation and detection were performed using an Agilent 1260 liquid chromatograph with a diode array detector (DAD) coupled to a mass selective detector (MSD). The chromatographic separation

was achieved using a reversed-phase Agilent ZORBAX Eclipse XDB-C18 column (2.1×150 mm, 3.5 µm particle size). The mobile phases were 0.1% formic acid in water (Mobile Phase A) and 0.1% formic acid in acetonitrile (Mobile Phase B). Initial conditions started at 45% B, ramping to 65% B over 12 minutes, followed by a final ramp to 95% B over 2 minutes, where it was held for 11 minutes before re-equilibration. Flow rate was maintained at 0.7 mL/min throughout, and the column temperature was set to 30° C. A 1.0 µL sample injection volume was used.

For MS detection, the following parameters were applied: gas temperature of 350° C, drying gas flow of 11 L/min, and a nebulizer pressure of 35 psig. The quadrupole temperature was set to 100° C. The mass spectrometer operated with a mass range of 40-2000 in the positive mode, a fragmentor voltage of 15 V, and a gain EMV of 1.00. The Vcap was set to +4000 V and -4000 V, with a step size of 0.10 and a % Cycle Time of 50.

11.2 H_2SO_4-based deBoc reaction and process development

Using **7-K** as the starting material, Boc deprotection was carried out using 9M of H_2SO_4,[5] yielding the corresponding amine L1.3-Ms. Subsequent coupling with Frag C afforded Len-API-H (Scheme S3).

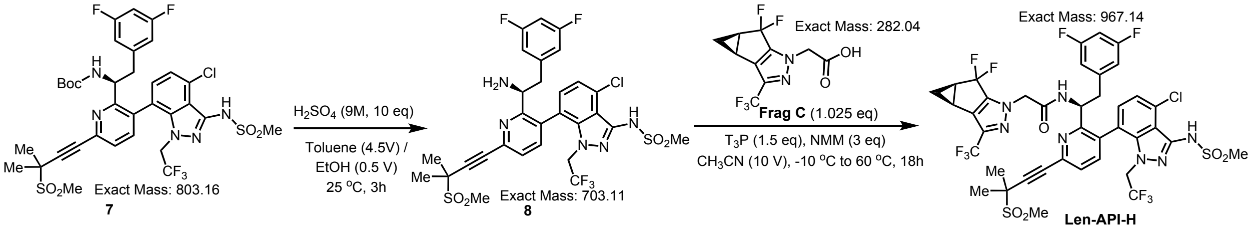

Scheme S3 TFA-promoted Boc-deprotection and the subsequent amidation with **Frag C** for **Len-API-H** synthesis.

11.2.1 Compound 8 In-Process and Assay Analysis

In process chromatographic analysis was performed using an Agilent 1100/1200 liquid chromatograph equipped with a diode array detector (DAD). Separation was achieved using an Agilent ZORBAX Eclipse XDB-C18 column (2.1×150 mm, 3.5 µm) maintained at a temperature of 30°C. The mobile phase consisted of a binary gradient elution program with 0.1% phosphoric acid in water (A) and acetonitrile (B) at a constant flow rate of 0.7 mL/min. Initial conditions were set to 45% B, ramping to 65% B over 12.00 min with a 2 min hold, followed by a final ramp to 95% B over 2 min with a 4 min hold. A post-run equilibration of 4 minutes was applied. Samples

were prepared at 1 mg/mL in acetonitrile. An injection volume of 1.0 µL was used. The detection was monitored at 275 nm. Figure S6 is a representative chromatogram for the various intermediates and impurities analyzed in the current method. **7**, **8** and **14** exist as atropisomers in solution and are observed as a pair of peaks on the chromatogram.

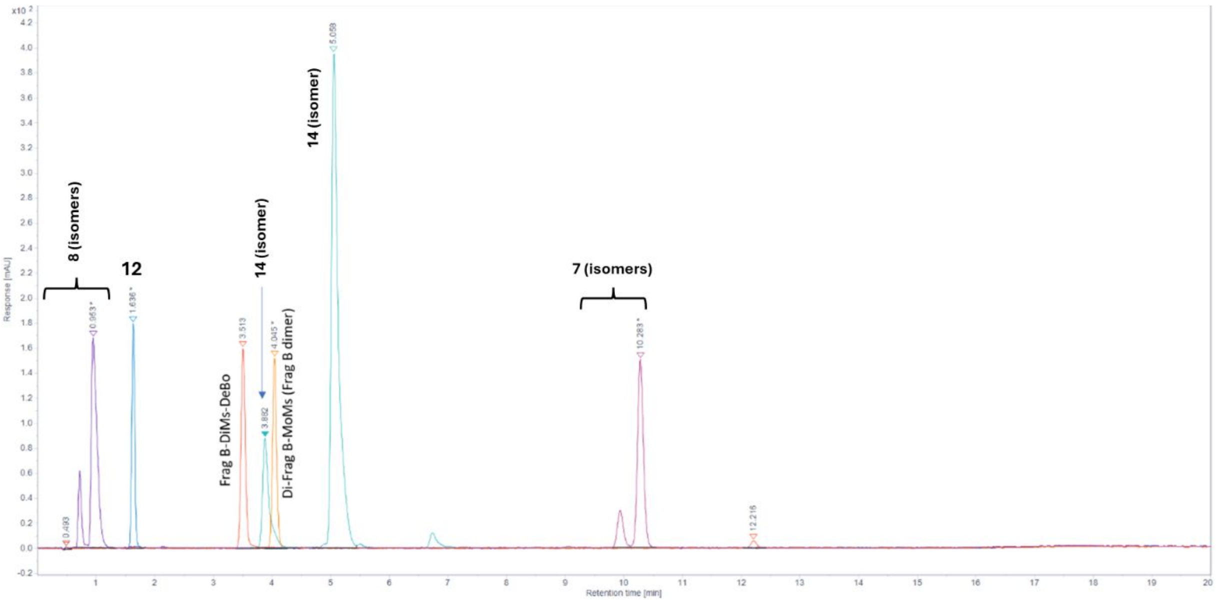

Figure S6 Representative chromatogram for **8**, starting materials and impurities.

Final **8** quantitation was performed via chromatographic analysis on an Agilent 1100/1200 liquid chromatograph equipped with a diode array detector (DAD). The separation was achieved using an Agilent ZORBAX Eclipse XDB-C18 column (2.1×150 mm, 3.5 µm) maintained at a temperature of 30°C. The mobile phases were 0.1% phosphoric acid in water (Mobile Phase A) and acetonitrile (Mobile Phase B). Initial conditions were set to 25% B, ramping to 50% B over 10 min and then to 95% B at 1 min. This composition was held until 9 min before a post-run equilibration of 4 minutes was initiated. Samples were prepared at a concentration of 1 mg/mL in acetonitrile, and an injection volume of 1.0 µL was used for all injections. The detection was monitored at 275 nm. Figure S7 is a representative chromatogram for the various intermediates and impurities analyzed in the current method. In this quantitative method for **8**, only the **8** atropisomers are separated while those of **7** co-elute.

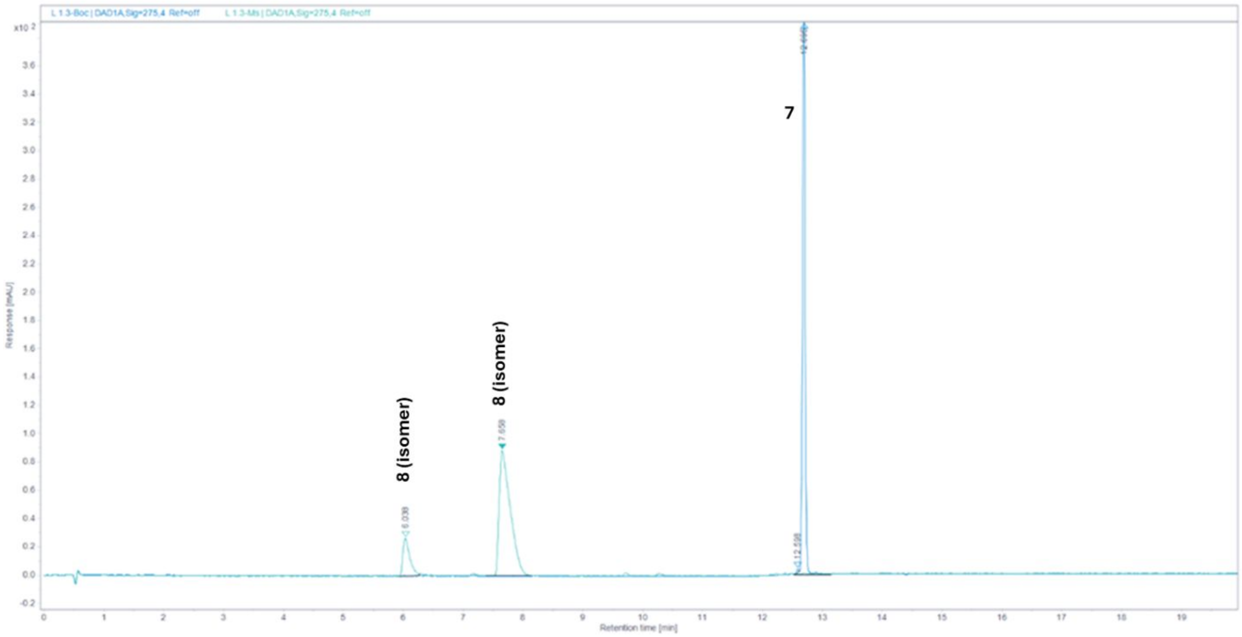

Figure S7 Representative chromatogram for **8** quantitation.

11.2.2 Compound 8 Relative Response Factors

Samples of **8**, impurities and synthetic intermediates were prepared at known concentrations and injected using the above method with a range of detection wavelengths to select an optimal wavelength for purity evaluation and reaction monitoring Figure S8. No true isosbestic point exists for this suite of analytes. As such, 275 nm was chosen for quantitation and monitoring purposes.

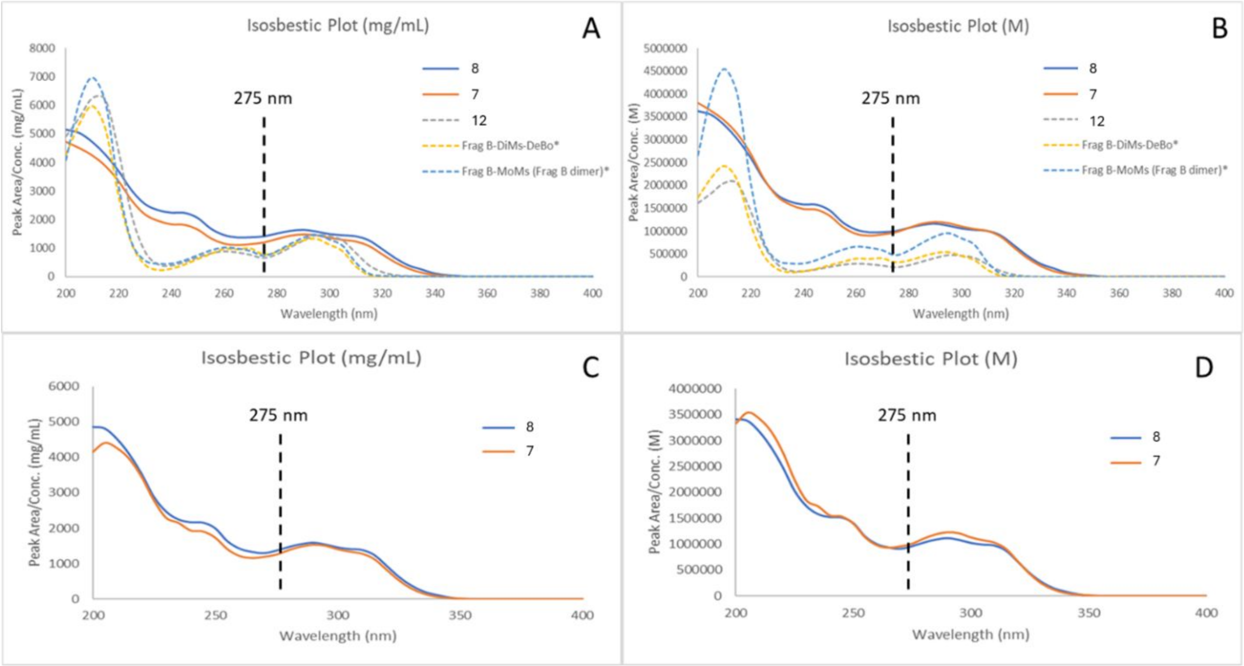

Figure S8 A) Isosbestic plot of L1.3-Ms and associated analytes based on mg/mL utilizing the in-process method (LCUV_Len API_Gradient). B) Isosbestic plot of L1.3-Ms and associated analytes based on molarity (M) utilizing the in-process method (LCUV_Len API_Gradient). C) Isosbestic plot of **8** and **7** (starting material) based on mg/mL utilizing the in-process method (LCUV_L1-3Ms). D) Isosbestic plot of **8** and **7** (starting material) based on molarity (M) utilizing the in-process method (LCUV_L1-3Ms).

Relative response factors (RRFs) were subsequently determined for starting materials and each available known impurity. Impurities were synthesized and purified in-house. Samples were prepared at 1 mg/mL in acetonitrile. Relative response factors were calculated using Equation S2. Table S7 lists the approximate retention times and RRFs (based on mg/mL and M basis) for each analyte.

$$\mathrm{RRF} = \frac{\left({}^{\mathrm{Area}}/{}_{\mathrm{Concentration}}\right)_{\mathrm{Analyte}}}{\left({}^{\mathrm{Area}}/{}_{\mathrm{Concentration}}\right)_{\mathrm{L1.3-Boc}}} \qquad \text{Eqn. S2}$$

Table S7 Relative response factors for Milestone 2 are calculated based on concentration (mg/mL)

Compound	Retention Time (min)	In process method (LCUV_Len API_Gradient)		Quantitation method (LCUV_L1-3Ms)	
		RRF at 275 nm (mg/mL)	RRF at 275 nm (M)	RRF at 275 nm (mg/mL)	RRF at 275 nm (M)
**8** (Product)	0.80 / 0.98 / 1.19 [a]	1.00	1.00	1.00	1.00
**12**	1.64	0.46	0.21	- [b]	- [b]
Frag B-DiMs-Debo	3.50	0.56	0.32	- [b]	- [b]
**14**	3.88 / 5.06	- [b]	- [b]	- [b]	- [b]
Di-Frag B-MoMs (Frag B dimer)	4.05	0.51	0.47	- [b]	- [b]
**7**	9.94 / 10.28 [a]	0.85	0.97	0.92	1.05

[a]Isomers, all RRFs were calculated using the sum of the isomer peak areas. [b]Not calculated

11.3 T3P-promoted amidation and process development

The synthesis of sodium **Len-API-Na** was accomplished via a T3P-promoted amide coupling between **8** and Frag C, using NMM as the base (Scheme S4). Following recrystallization from EtOH/heptane, **Len-API-Na** was obtained.

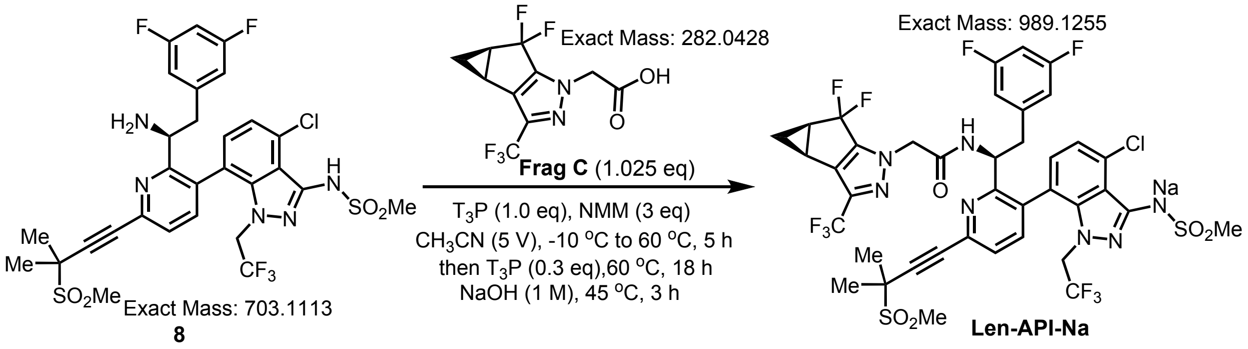

Scheme S4 T3P-promoted amide coupling for synthesis of **Len-API-Na**.

11.3.1 Len-API-Na In-Process and Assay Analysis

Chromatographic analysis was performed on an Agilent 1100/1200 liquid chromatograph equipped with a diode array detector (DAD). Separation was achieved using an Agilent ZORBAX Eclipse XDB-C18 column (2.1×150 mm, 3.5 μm) maintained at a temperature of 30°C. The mobile phase consisted of a binary gradient elution program with 0.1% phosphoric acid in water

(A) and acetonitrile (B) at a constant flow rate of 0.7 mL/min. Initial conditions were set to 45% B, ramping to 65% B over 12.00 min with a 2 min hold. A final ramp to 95% B over 2 min with a 4 min hold. A post-run equilibration of 4 minutes was applied. Samples were prepared at 1 mg/mL in acetonitrile. An injection volume of 1.0 µL was used. The detection was monitored at 235 nm. Figure S9 is a representative chromatogram for the various intermediates and impurities analyzed in the current method. It should be noted that **Len-API-Na**, **8**, **13** and **14** are observed as a pair of peaks due to atropisomerism in solution.

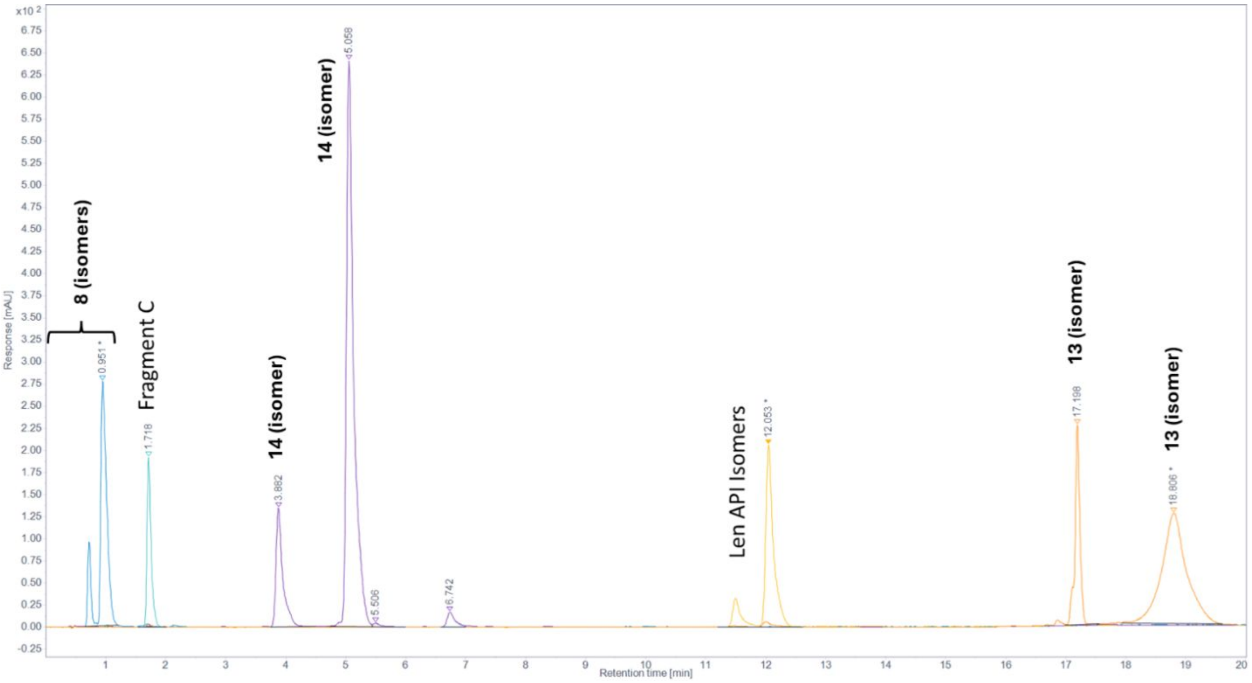

Figure S9 Representative chromatogram for **Len-API-Na** synthesis.

11.3.2 Len-API-Na Relative Response Factors

Samples of **Len-API-Na**, impurities and synthetic intermediates were prepared at known concentrations and injected using the above method with a range of detection wavelengths to select an optimal wavelength for purity evaluation and reaction monitoring Figure S10. No true isosbestic point exists for this suite of analytes. As such, 235 nm was chosen for quantitation and monitoring purposes.

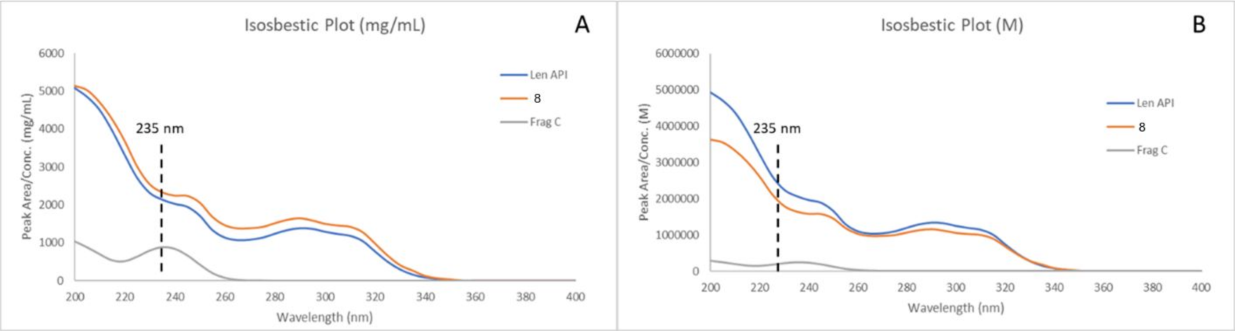

Figure S10 A) Isosbestic plot of **Len-API-Na** and associated analytes based on mg/mL. B) Isosbestic plot of **Len-API-Na** and associated analytes based on molarity (M).

Relative response factors (RRFs) were subsequently determined for starting materials and each available known impurity. Impurities were synthesized and purified in-house. Samples were prepared at 1 mg/mL in acetonitrile. Relative response factors were calculated using Equation S3. Table S8 lists the approximate retention times and RRFs (based on mg/mL and M basis) for each analyte.

$$\mathrm{RRF} = \frac{\left({}^{\mathrm{Area}}/_{\mathrm{Concentration}}\right)_{\mathrm{Analyte}}}{\left({}^{\mathrm{Area}}/_{\mathrm{Concentration}}\right)_{\mathrm{Len-API}}} \qquad \text{Eqn. S3}$$

Table S8 Relative response factors for Milestone 1 are calculated based on concentration (mg/mL)

Compound	Retention Time (min)	RRF at 235 nm (mg/mL)	RRF at 235 nm (M)
**8**	0.80 / 0.98 / 1.19[a]	1.09	0.80
**Frag C**	1.72	0.41	0.12
**14**	3.88 / 5.05	-[b]	-[b]
**Len-API-Na** (Product)	11.50 / 12.05 [a]	1.00	1.00
**13**	17.2 / 18.8	-[b]	-[b]

[a]Isomers, all RRFs were calculated using the sum of the isomer peak areas. [b]Not calculated.

11.4 LC-UV_Len API_Gradient Method

Structures & IDs:

Step 1

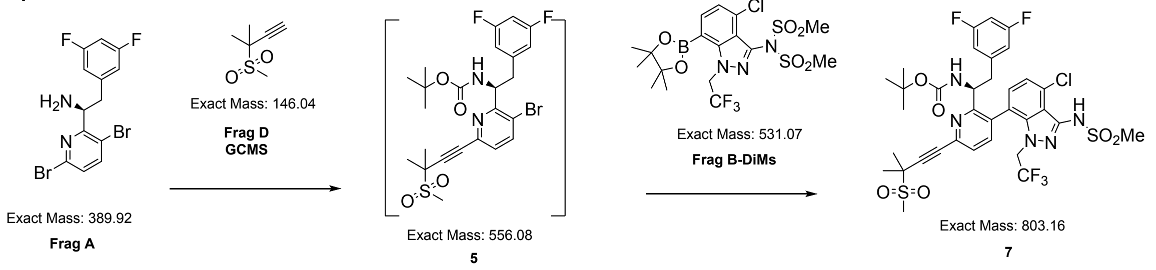

Step 1 Major Impurities:

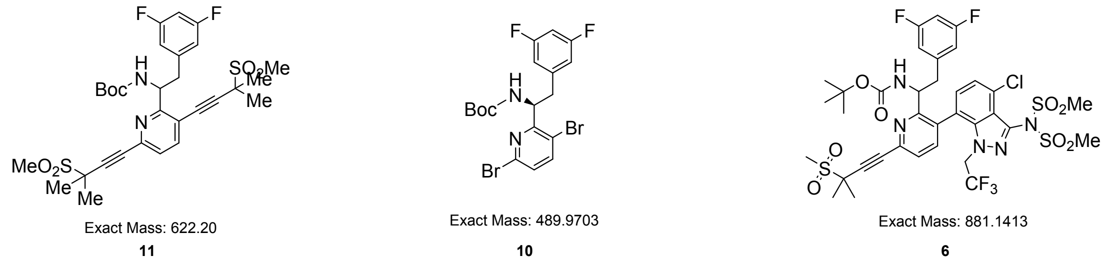

Step 2

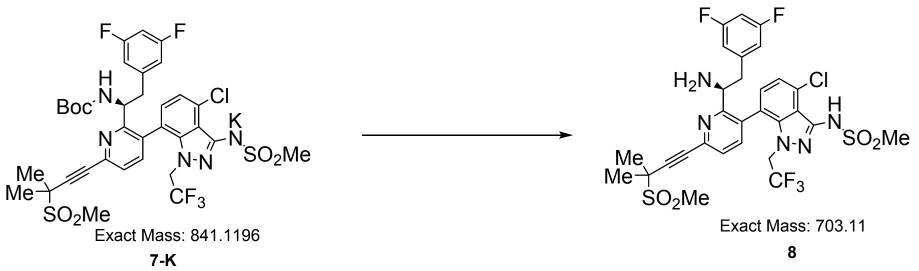

Step 2 Major Impurities:

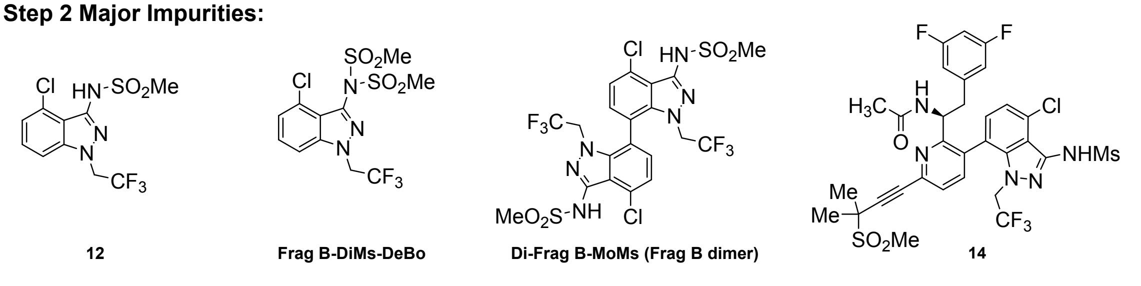

Step 3

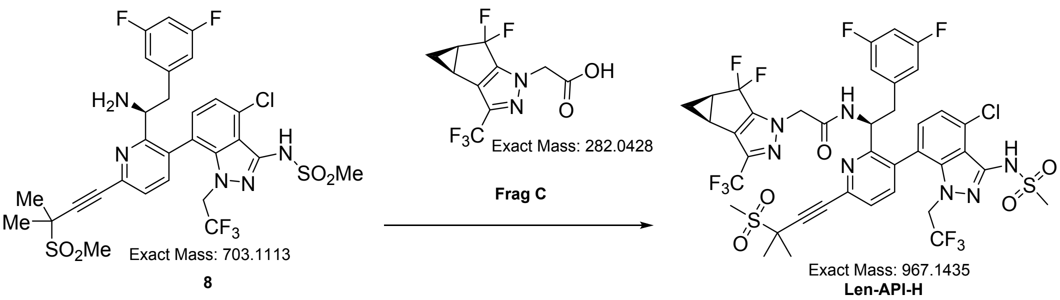

Step 3 Major Impurities:

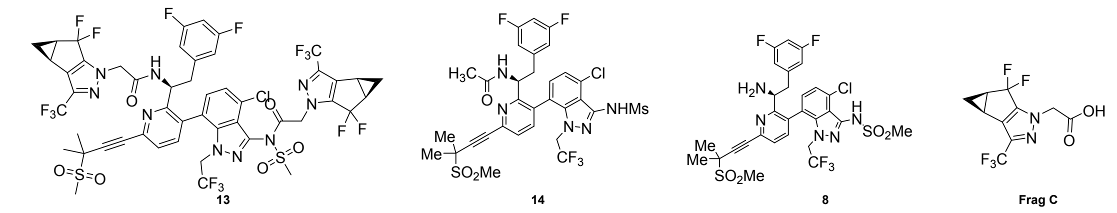

Conditions:

Column: Agilent ZORBAX Eclipse XDB-C18, 2.1 x 150 mm, 3.5 µm
Mobile Phase A: 0.1% phosphoric acid in water
Mobile Phase B: Acetonitrile
Injection volume: 1.0 µL   Column temp: 30°C   Flow rate: 0.7 mL/min
Detector wavelength(s): Main: 275 nm (Step 1 and Step 2), 235nm (Step 3)

Sample preparation: Prepare samples at 1 mg/mL in acetonitrile

LC Gradient Table:

Time (min)	%A	%B
0.00	55	45
12.00	35	65
14.00	35	65
16.00	5	95
20.00	5	95

Post-run equilibration: 4 minutes

Retention Times			
**Compound**	**Time (min)**	**Relative RF (mg/mL) ***	**Relative RF (M)***
**Step 1 - LenAPI_Step 1_Processing, 275 nm**			
**Frag A**	0.56	0.54	0.26
**11**	8.46	1.83	1.42
**7**	9.94 / 10.28	1.00	1.00

**5**	11.12	0.75	0.52
**Frag B-DiMs**	12.30	0.41	0.27
**6**	12.09 / 12.67	-	-
**10**	14.38	0.60	0.37
**Step 2 - LenAPI_Step 2_Processing, 275 nm**			
**8**	0.80 / 0.98 / 1.19	1.00	1.00
**12**	1.64	0.46	0.21
**Frag B-DiMs-Debo**	3.50	0.56	0.32
**14**	3.88 / 5.06	-	-
**Di-Frag B-MoMs (Frag B dimer)**	4.05	0.51	0.47
**7**	9.94 / 10.28	0.85	0.97
**Step 3 - LenAPI_Final Step_Processing, 235 nm**			
**8**	0.80 / 0.98 / 1.19	1.09	0.80
**14**	3.88 / 5.06	-	-
**Frag C**	1.72	0.41	0.12
**Len-API-Na**	11.50 / 12.05	1.00	1.00
**13**	17.2 / 18.8	-	-

Notes: Frag D has very low absorbance. For processing samples use the processing method associated with the step.

Where an analyte exists as multiple isomers, RRF was calculated using the sum of all isomer peaks.

Representative UV Spectra

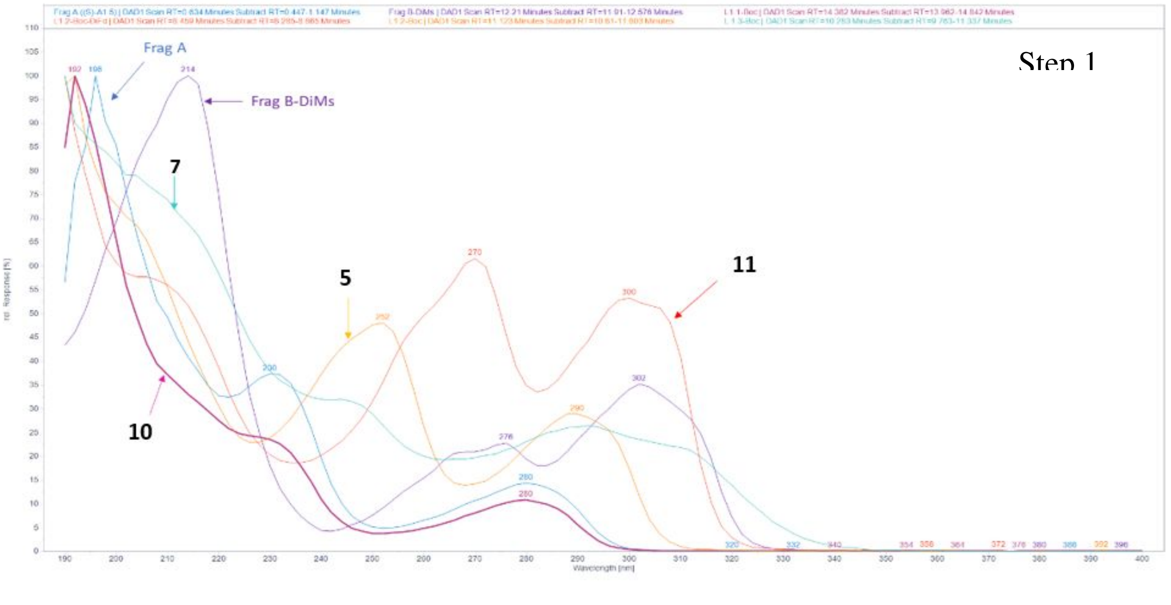

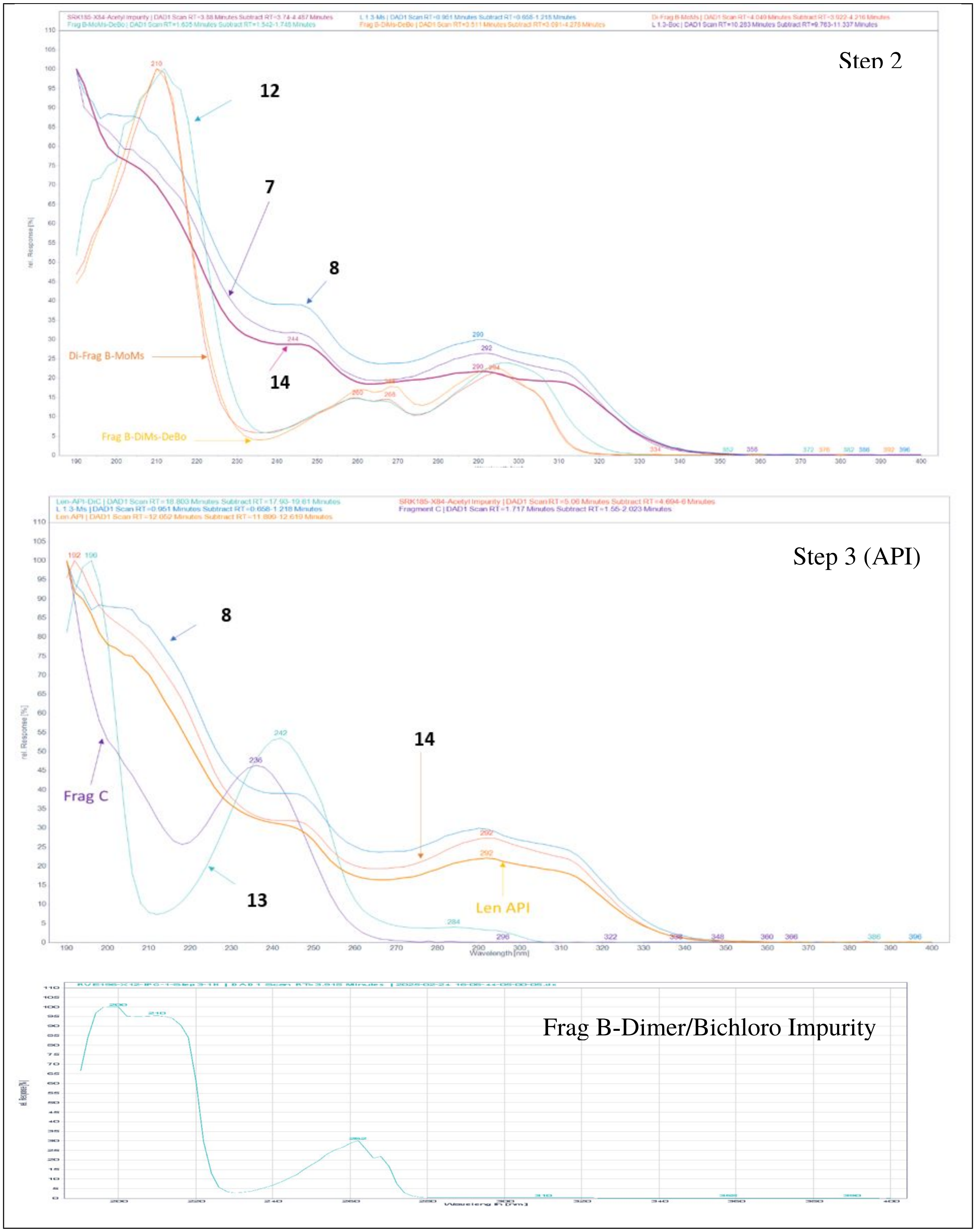

11.5 LCUVA_L 1.3-Ms Method

**Structures & IDs:**

Step 2

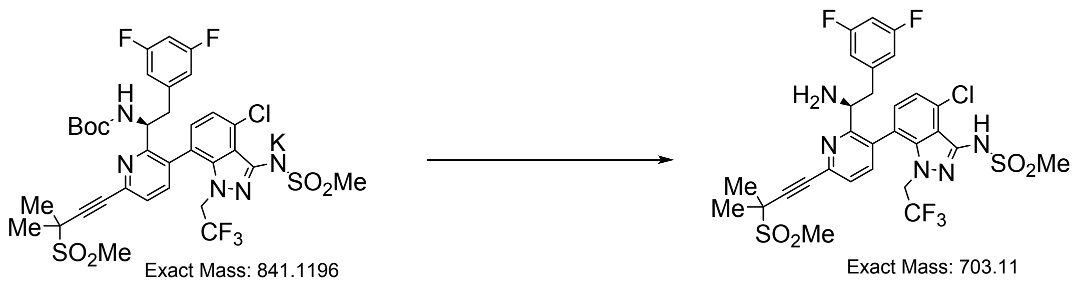

7-K | 8

Conditions:

Column: Agilent ZORBAX Eclipse XDB-C18, 2.1 x 150 mm, 3.5 μm

Mobile Phase A: 0.1% phosphoric acid in water

Mobile Phase B: Acetonitrile

Injection volume: 1.0 μL   Column temp: 30°C   Flow rate: 0.7 mL/min

Detector wavelength(s): Main: 275 nm

LC Gradient Table:

Time (min)	%A	%B
0.00	75	25
10.00	50	50
11.00	5	95
20.00	5	95

Post-run equilibration: 4 minutes

Sample preparation: Prepare samples at 1 mg/mL in acetonitrile

Retention Times			
**Compound**	**Time (min)**	**Relative RF (mg/mL) ***	**Relative RF (M)***
**Step 2 - LenAPI_Step 2_Processing, 275 nm**			
**8**	6.04 / 7.66	1.00	1.00
**7-K (7)**	12.70	0.92	1.05

Notes: Where an analyte exists as multiple isomers, RRF was calculated using the sum of all isomer peaks.

Representative UV Spectra

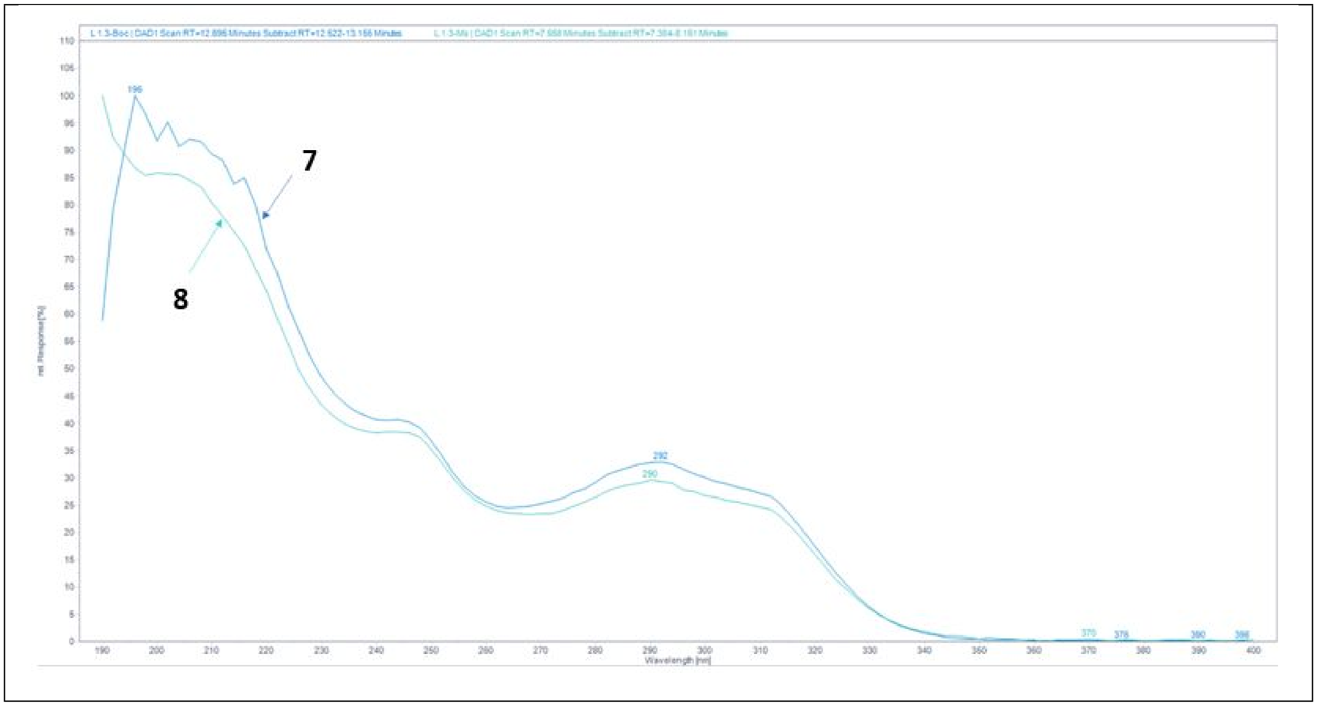

11.6 Solvent Method (GC-FID)

Conditions:

Column: HP-1; 30M X 320 µm; 5 µm film

Inlet Pressure: 4.8 psi

Split Ratio: 50:1

Split Flow: 39.439 mL/min

Column flow: 0.787 mL/min

Injection Temp: 260 °C

Injection volume: 1 µL

Solvent Delay: N/A

Runtime: 20 min

Temperature Program:

Time (min)	Temp (°C)	Ramp (°C/min)	Hold (min)
0	50	0	5
20	235	20	5.75

FID Parameters:

Heater (°C)	200
Air Flow (mL/min)	450
H_2 Fuel Flow (mL/min)	35
N_2 Makeup Flow (mL/min)	30

Sample preparation: Prepare samples at 5-10 mg/mL in acetonitrile or other suitable solvent.

Retention Times

Compound	Time (min)	Compound	Time (min)
Methanol (MeOH)	5.46	Heptane (n-heptane)	12.12
Acetonitrile (ACN)	7.65	Methyl isobutyl ketone (MIBK)	12.46
Acetone	7.87	Toluene	13.05
2-Propanol (IPA)	8.2	Dimethyl Sulfoxide (DMSO)	13.37
Methyl *t*-Butyl Ether (MTBE)	9.85	Hexanes	10.26, 10.52, 10.63
2-Butanone (MEK)	10.10		
Ethyl Acetate (EtOAc)	10.41		
n-Hexane (hexane)	10.52		
Chloroform	10.63		
2-Methyl Tetrahydrofuran (2-MeTHF)	11.62		

Representative Chromatograms

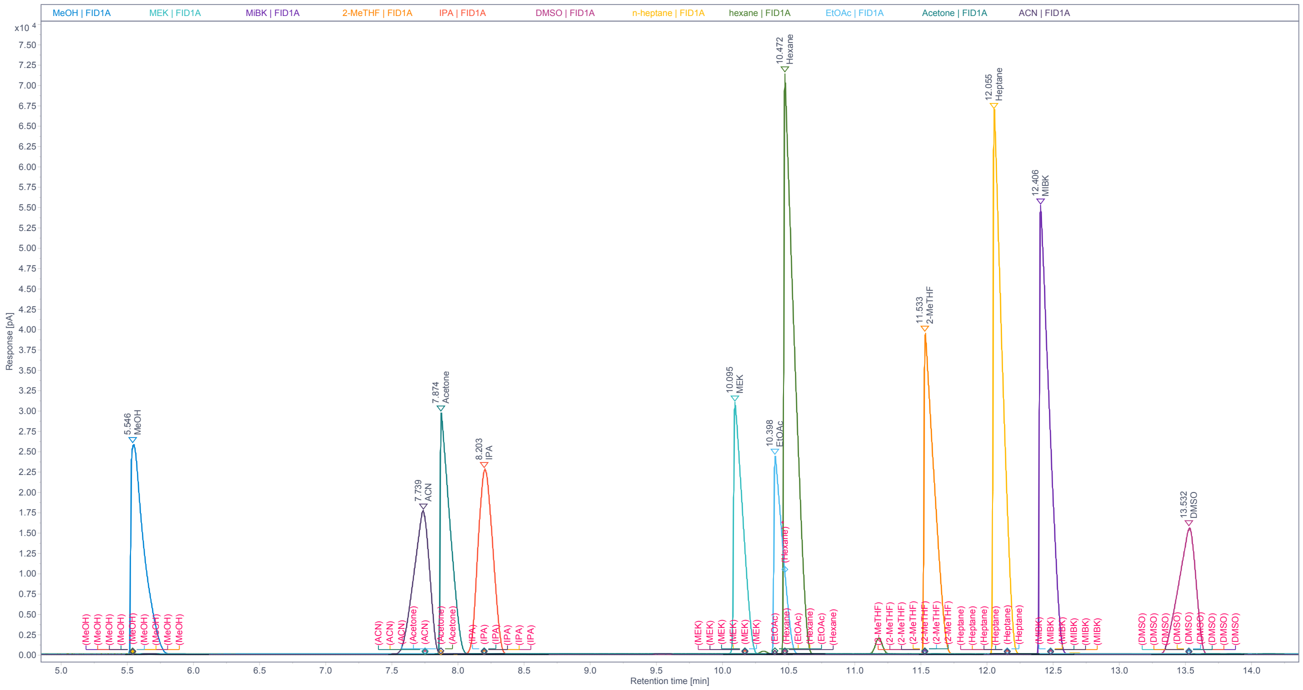

11.7 LCMS Len API_Gradient Method (LC-MS)

Instrument Type: Agilent 1260 liquid chromatograph (LC) with diode array detector (DAD) and 6125 mass spectrometer detector (MSD)

Conditions:

Column: Agilent ZORBAX Eclipse XDB-C18, 2.1 x 150 mm, 3.5 µm

Mobile Phase A: 0.1% formic acid in water

Mobile Phase B: 0.1% formic acid in Acetonitrile

Injection volume: 1.0 µL   Column temp: 30°C   Flow rate: 0.7 mL/min

Detector wavelength(s): Main: 235nm, 275nm

LC Gradient Table:

Time (min)	%A	%B
0.00	55	45
12.00	35	65
14.00	35	65
16.00	5	95
25.00	5	95

Post-run equilibration: 5 minutes

MSD Parameters:

Source Parameters			Signal 1 (+)
Gas Temp.	350 °C	Time (min)	0.00
Drying Gas	11 L/min	Mass Range	40-2000
Neb. Pressure	35 psig	Fragmentor	15
Quad Temp.	100 °C	Gain EMV	1.00
VCap (+)	4000 V	Threshold	0
VCap (-)	4000 V	Step Size	0.10
		% Cycle Time	50

Sample preparation: Prepare samples at 1 mg/mL in acetonitrile

Retention Times

**Compound**	**Time (min)**	***m/z***
**8**	0.9 / 1.0 / 1.2	704 $[M+H]^+$; 1407 $[2M+H]^+$
Frag C	2.0	283 $[M+H]^+$

Frag B-DiMs-Debo	3.7	406 [M+H]$^+$
**14**	4.8 / 6.1	746 [M+H]$^+$
Di-Frag B-MoMs	5.1	653 [M+H]$^+$; 1327 [2M+Na]$^+$
**Frag A**	5.7	393 [M+H]$^+$; 434 [M+ACN+H]$^+$
**5**	10.6	503 [M-Boc+H]$^+$; 537 [M+H]$^+$
Frag B-DiMs	10.7	532 [M+H]$^+$; 549 [M+NH$_4$]$^+$
**7-K (7)**	10.9/11.3	748 [M-Boc+H]$^+$; 804 [M+H]$^+$
Len-API-Na (Len-API-H)	12.9 / 13.5	968 [M+H]$^+$
**10**	15.5	437 [M+H]$^+$
**13***	17.5	1233 [M+H]$^+$

*Speculated based on mass, impurity not isolated for full characterization or other method development.

Notes: Frag D exhibits low UV absorbance making it difficult to detect via this method. L 1.3-Ms has three peaks

11.8 LC-ELSD Salts-HILIC_ELSD Method

Instrument Type: Agilent 1100 liquid chromatograph (LC) with 1260 electron light scattering detector (ELSD)

Conditions:

Column: Agilent InfinityLab Poroshell 120 HILIC-Z, 3.0 x 150 mm, 2.7 µm

Mobile Phase A: 10 mM ammonium acetate, pH 4

Mobile Phase B: 90:10, acetonitrile: 10mM ammonium acetate, pH 4:

Injection volume: 1.5 µL   Column temp: 30 °C   Flow rate: 0.8 mL/min

Detector: ELSD

LC Gradient Table:

Time (min)	%A	%B
0	10%	90%
1	10%	90%
6	20%	80%
11	80%	20%
15	80%	20%

Post-run equilibration: 3 min

ELSD Parameters:

Evaporator Temp.	30 °C
Nebulizer Temp.	30 °C
Gas Flow Rate (SLM)	1.60
Data Rate (Hz)	80
LED Intensity	100%
Smoothing	30
PMT Gain	1.0

Sample preparation: 2mg/mL in appropriate solvent.

Retention Times

**Compound**	**Time (min)**
Chloride	2.38
Sodium	3.95
Potassium	4.23
Sulfate	8.92

Representative Chromatograms

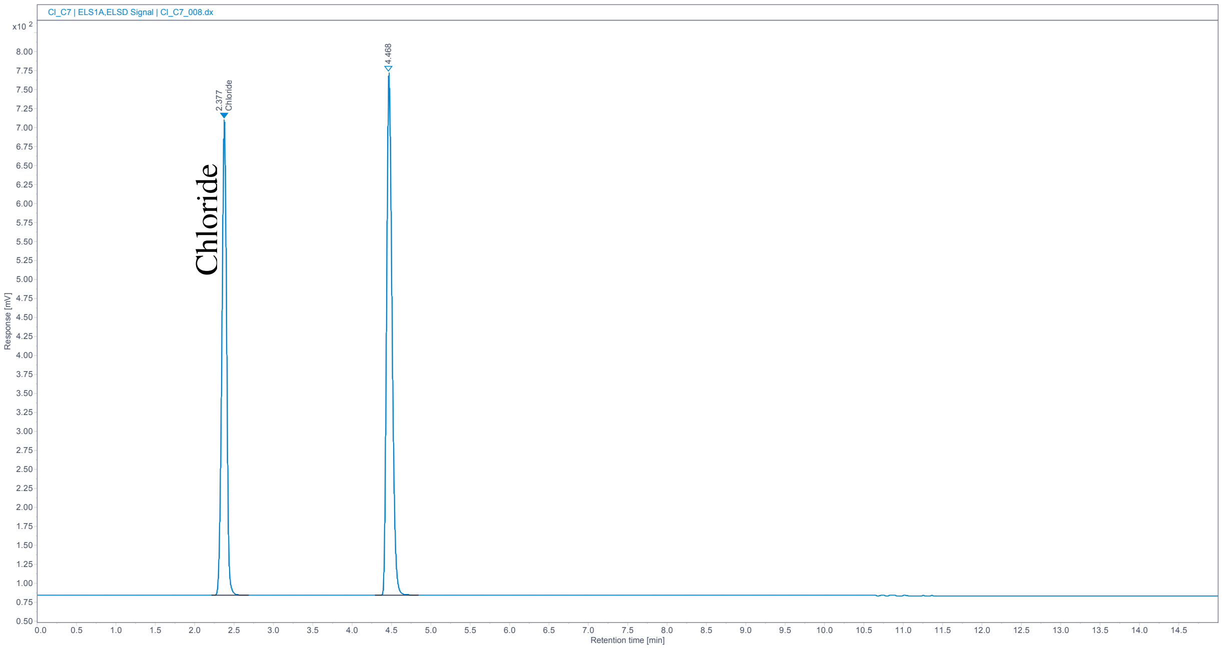

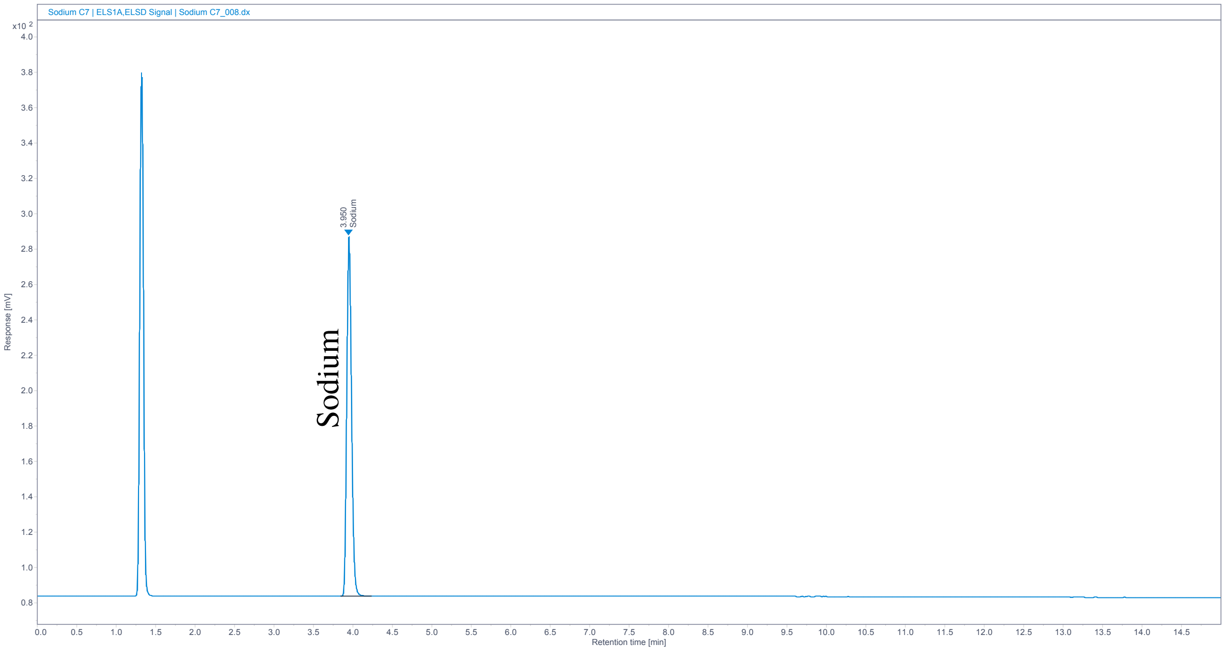

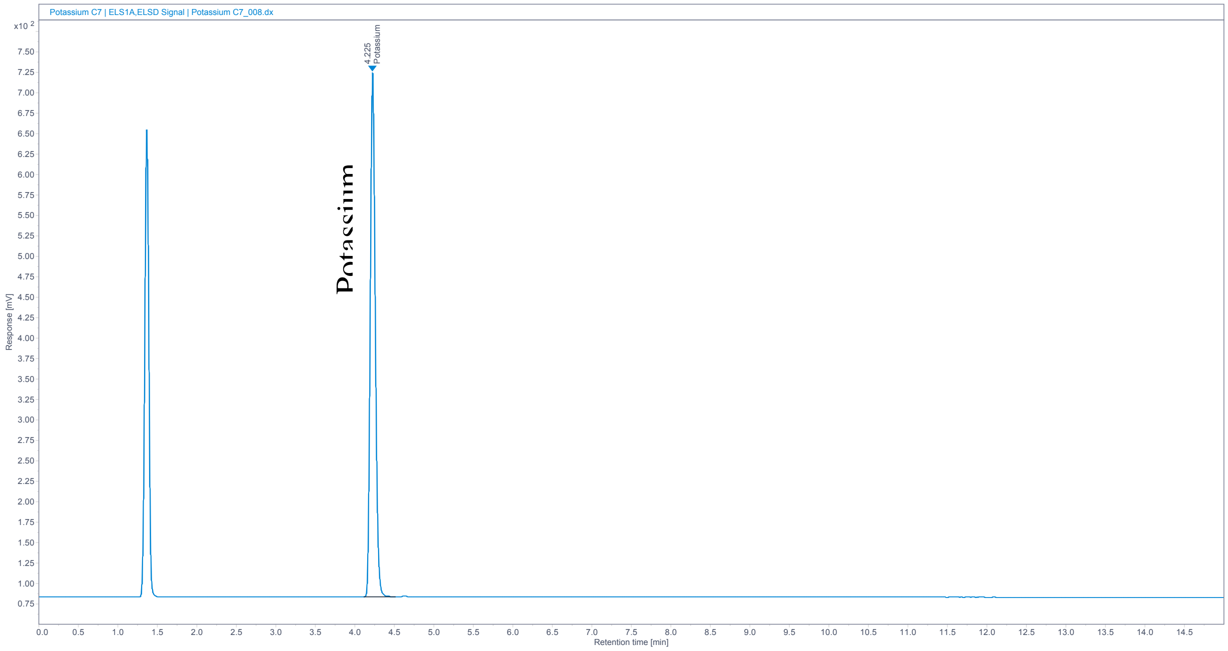

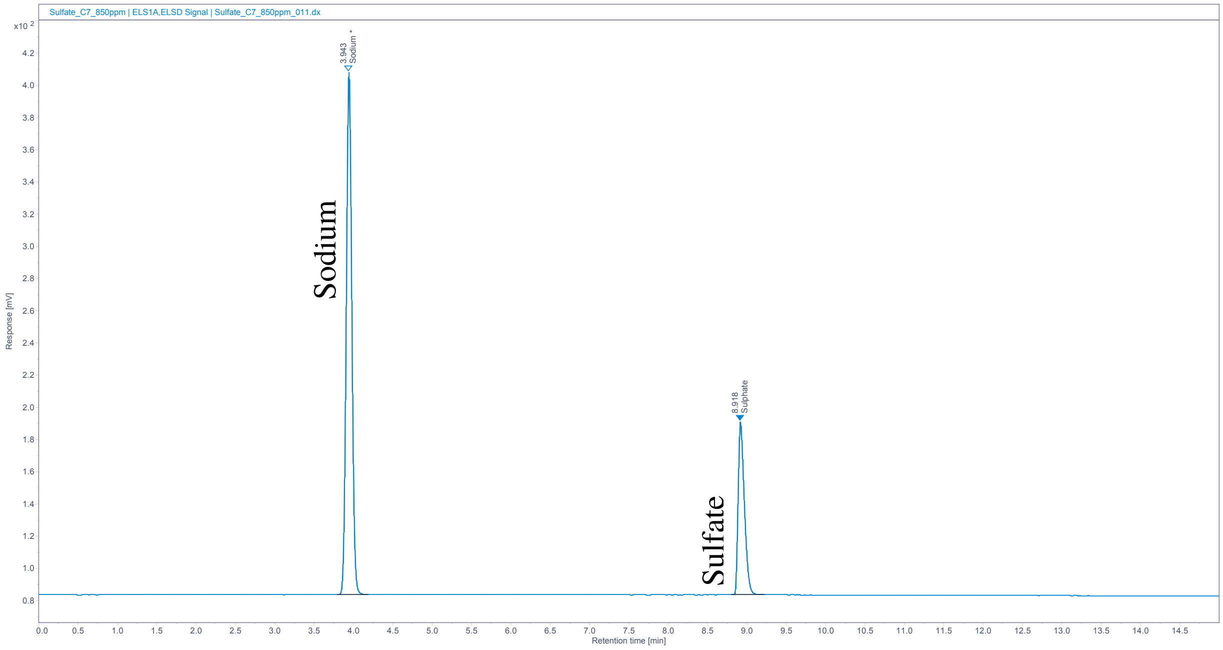

11.9 ICP-OES Method

ICP-OES Conditions:

RF Power (kW)	1.4	Spray Chamber	Glass Cyclonic

Plasm Gas Flow (L/min)	12.0	Nebulizer	SeaSpray
Auxiliary Gas Flow (L/min)	1.0	Uptake Rate (mL/min)	0.8
Nebulizer Gas Flow	0.7	View Mode	SVDV
Pressure (kPa)	220	replicate read time (sec)	5
		stabilization time (sec)	15
		sample uptake delay time (sec)	10

Notes: The most intense emission line is generally selected for optimal sensitivity and a strong signal-to-noise ratio. Check for potential spectral interferences from other elements in the sample matrix, choose a secondary, less intense line that is free from any overlap.

Sample preparation: Weigh at least 50 mg of the sample and add 5 mL of 10% HCl in methanol. To dissolve the contents, vortex and/or sonicate the mixture. Transfer a 1 mL aliquot of this solution to a new tube, then bring the final volume to 10 mL with 10% HCl in deionized water. If any solids remain, filter the final solution using a 32mm PTFE syringe filter (0.45µm).

Representative Spectra

Palladium (340.458 nm)

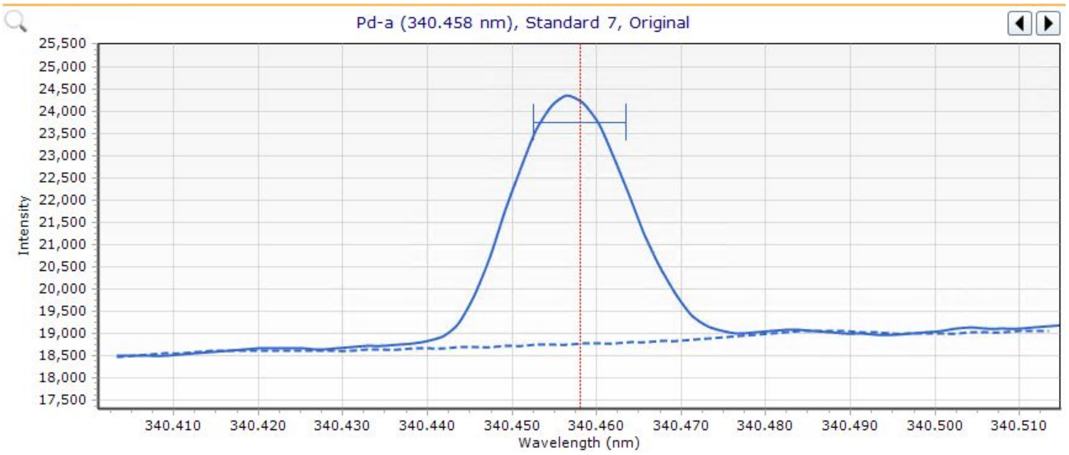

11.10 General_ThinFilm_100to1split_MS (GC-MS)

Instrument Type: Agilent 8890 gas chromatograph (GC) with a 5977 mass spectrometer detector (MSD)

Structures & IDs:

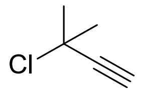

Exact Mass: 102.02
3-CMB

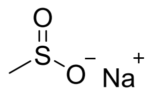

Exact Mass: 101.98
Sodium methanesulfinate

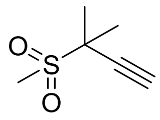

Exact Mass: 146.04
Fragment D

Conditions:

Column: J&W HP-5ms GC Column, 30 m, 0.25 mm, 0.25 μm, 7 inch cage

Inlet Pressure: 6.71 psi   Split Ratio: 100:1   Split Flow: 91.8 mL/min

Column flow: 0.92 mL/min   Injection Temp: 250°C   Injection volume: 1.0 μL

Total Flow: 95.7 mL/min   Solvent Delay: 3.0 min   Runtime: 19.0 min

Temperature Program:

Temp (°C)	Ramp (°C/min)	Hold (min)
50	-	3.0
250	25	3.0
300	25	3.0

MS Parameters:

Transfer Line Temp (°C)	250
Source Temp (°C)	230
Quad Temp (°C)	15
Electron Energy (eV)	70
Mass Range	40 - 700

Sample preparation: ~1.0 mg/mL in ACN

Retention Times

**Compound**	**Time (min)**	***m/z***
Fragment D	7.2	100, 67

Representative Chromatogram

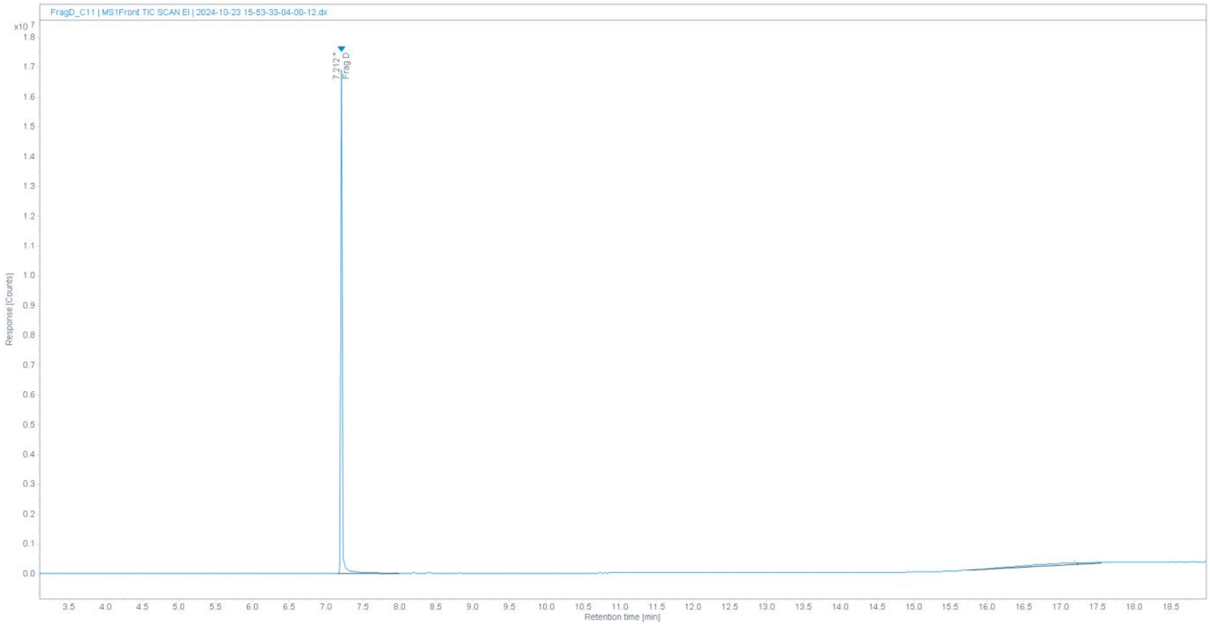

12 NMR and HPLC Spectra

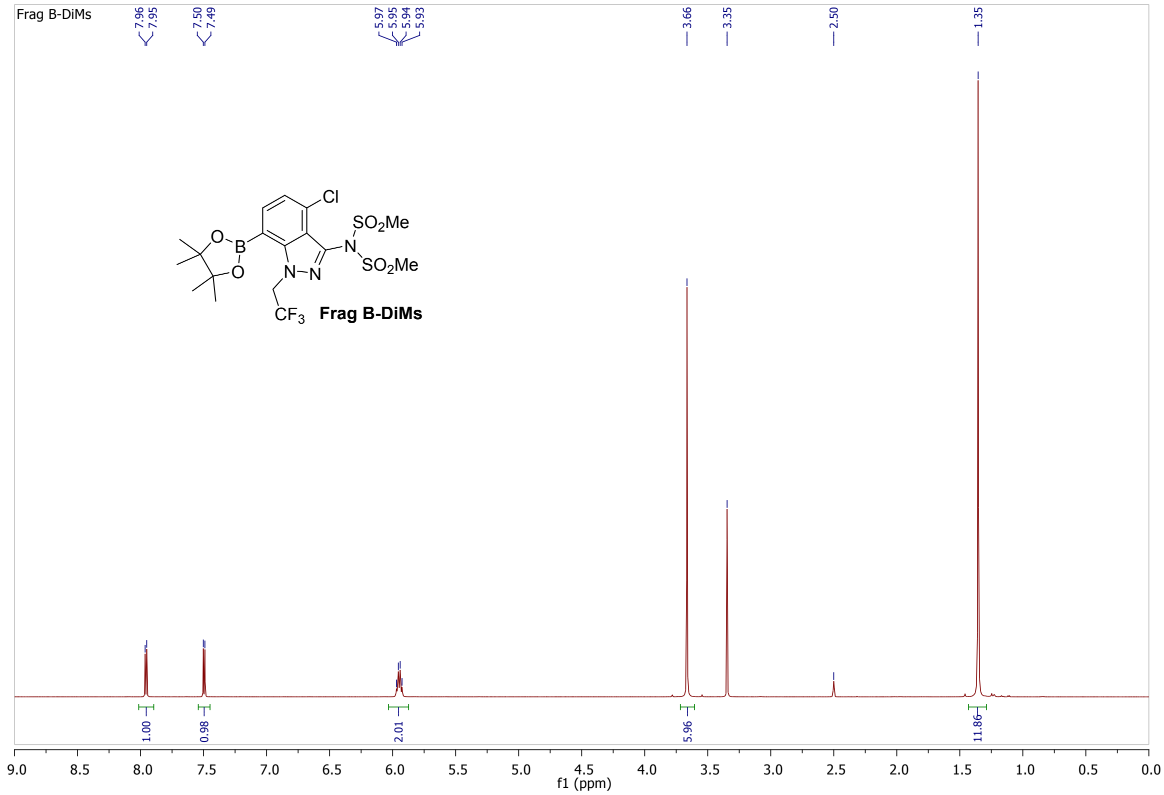

Figure S11. ^{1}HNMR (600MHz, DMSO-d_6) of **Frag-B-DiMs**.

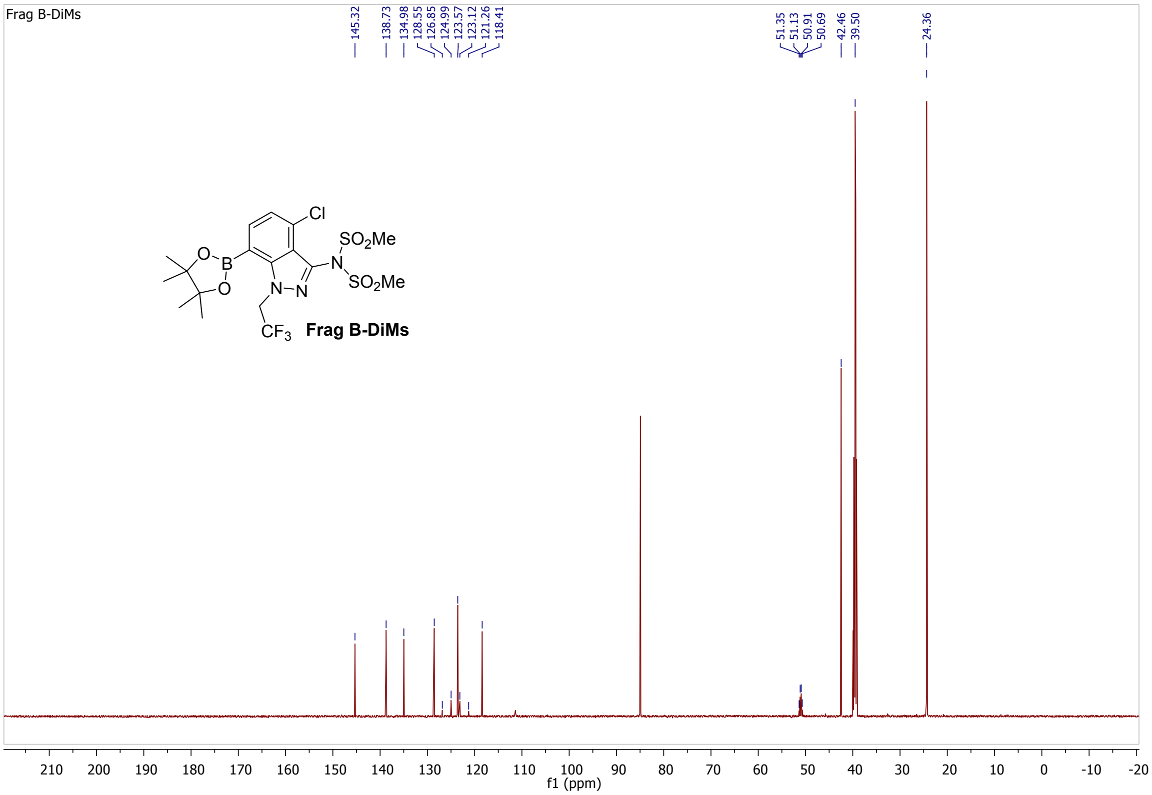

Figure S12. ^{13}CNMR (151MHz, DMSO-d_6) of **Frag-B-DiMs**.

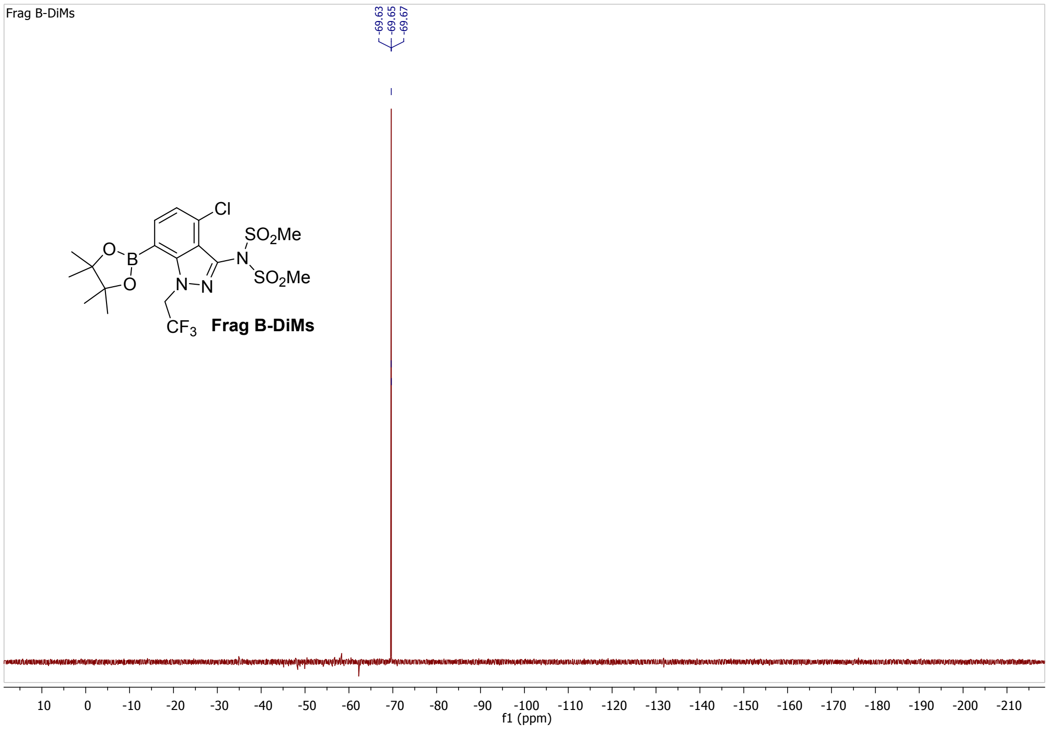

Figure S13. ^{19}FNMR (565MHz, DMSO-d$_6$) of **Frag-B-DiMs**.

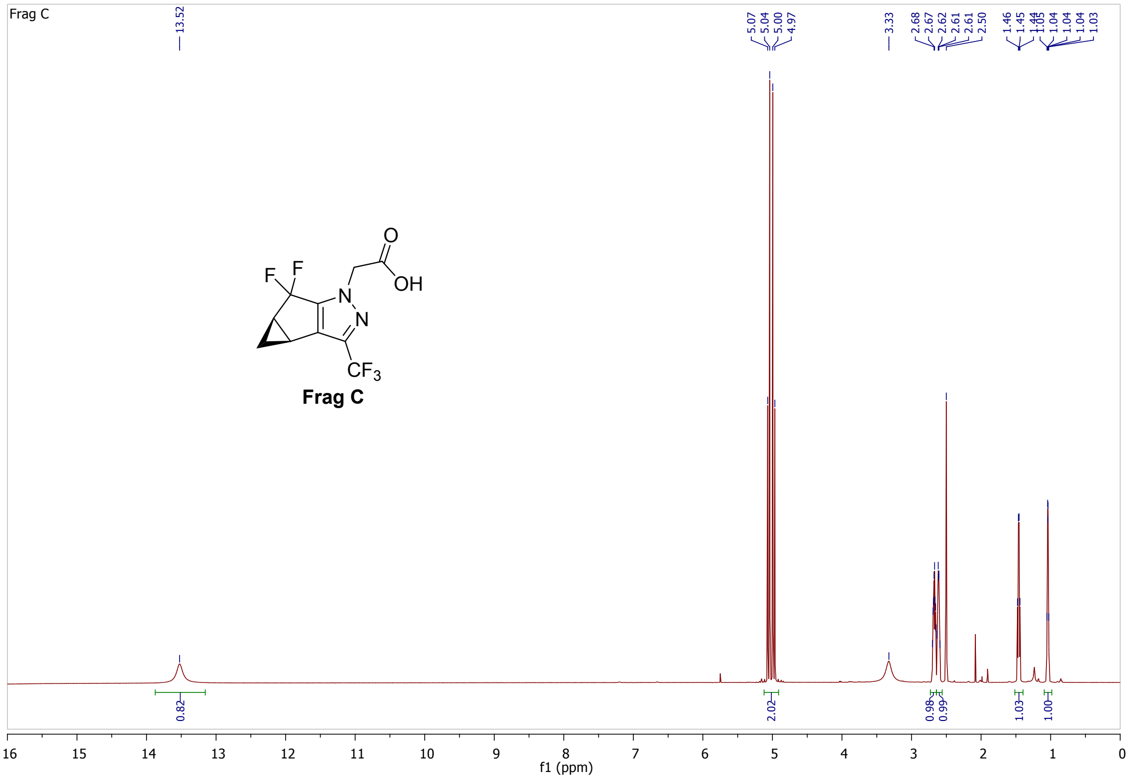

Figure S14. ^{1}HNMR (600MHz, DMSO-d$_6$) of **Frag C**.

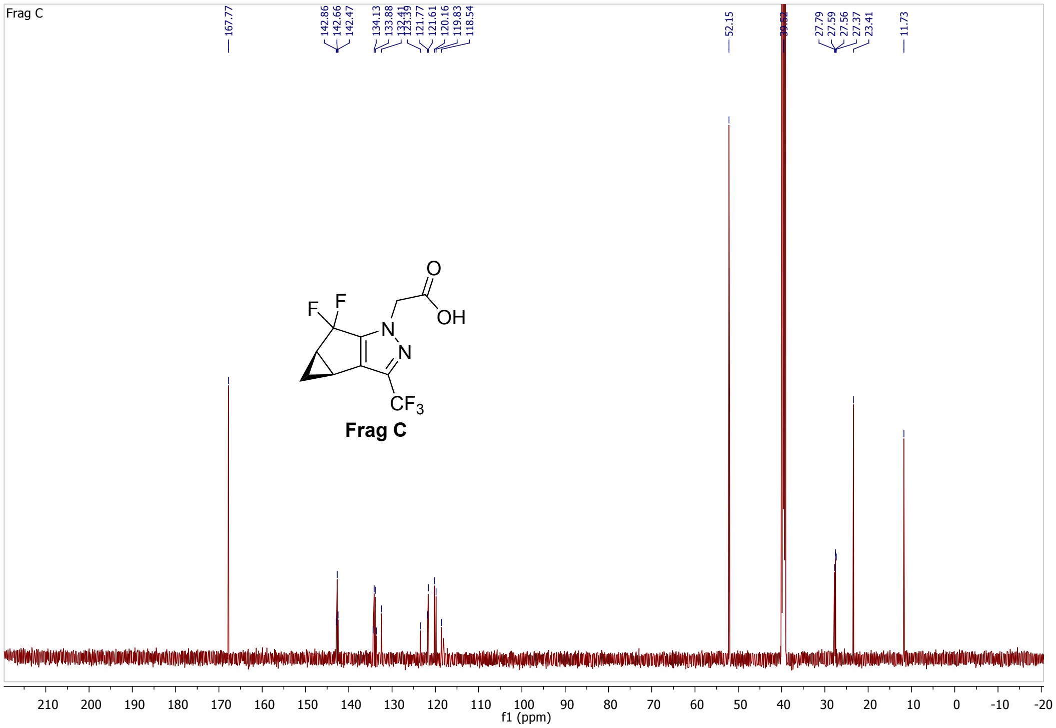

Figure S15. ^{13}CNMR (151MHz, DMSO-d_6) of **Frag C**.

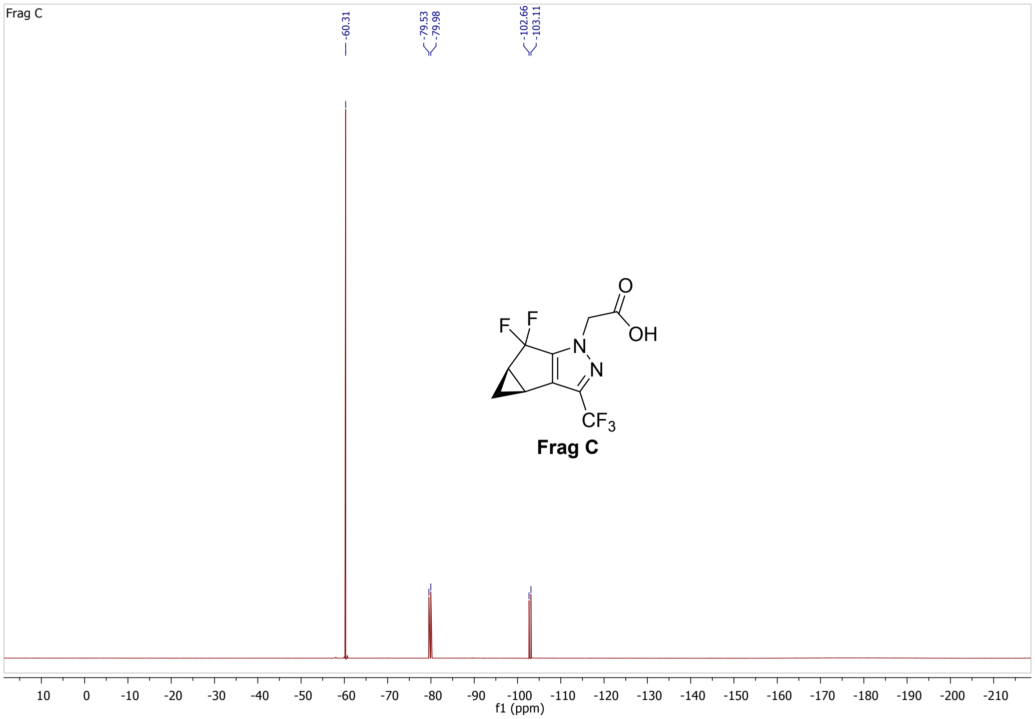

Figure S16. ^{19}FNMR (565MHz, DMSO-d_6) of **Frag C**.

Retention Times	
**Compound**	**Time (min)**
**Frag C** Enantiomer 1	3.53
**Frag C** Enantiomer 2	8.52
**Notes:** Stereochemical assignments unknown	

Representative Chromatogram

Frag C (racemic)

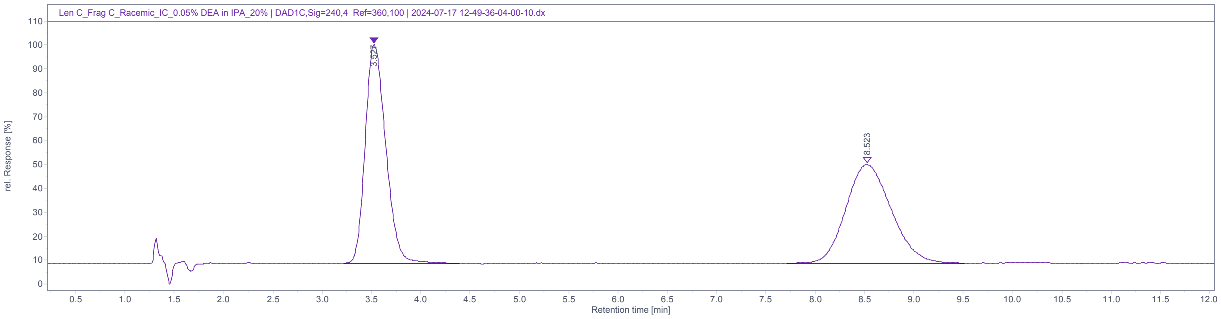

Frag C (Chiral)

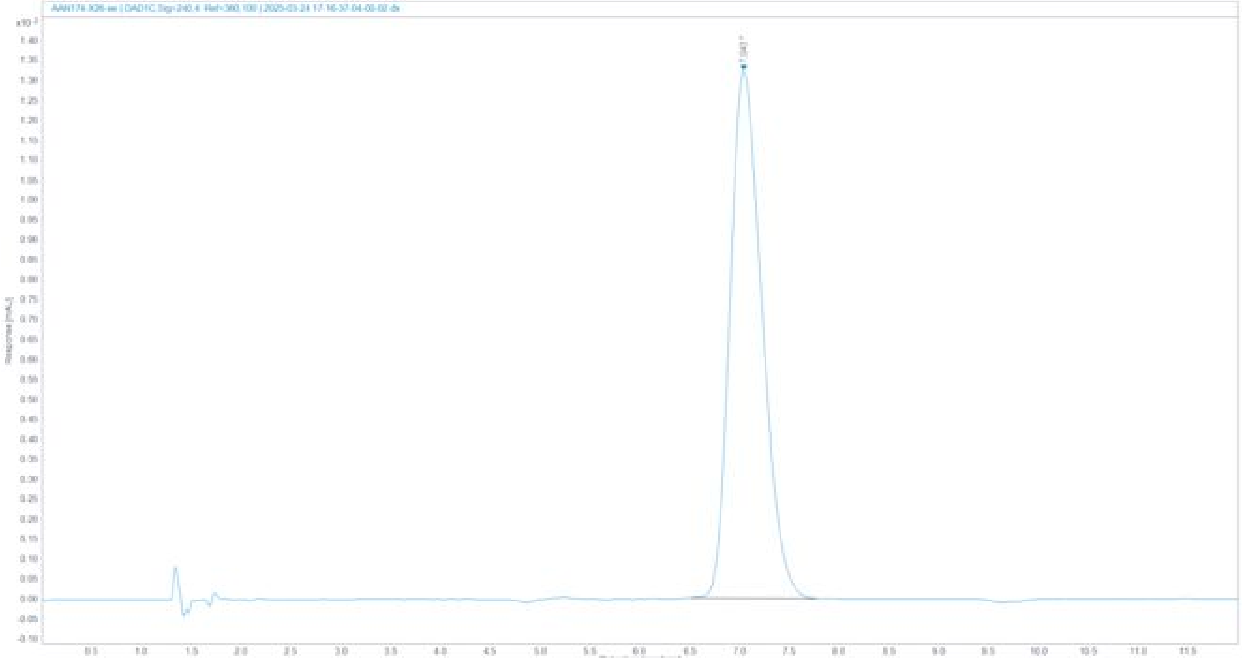

UV Spectra

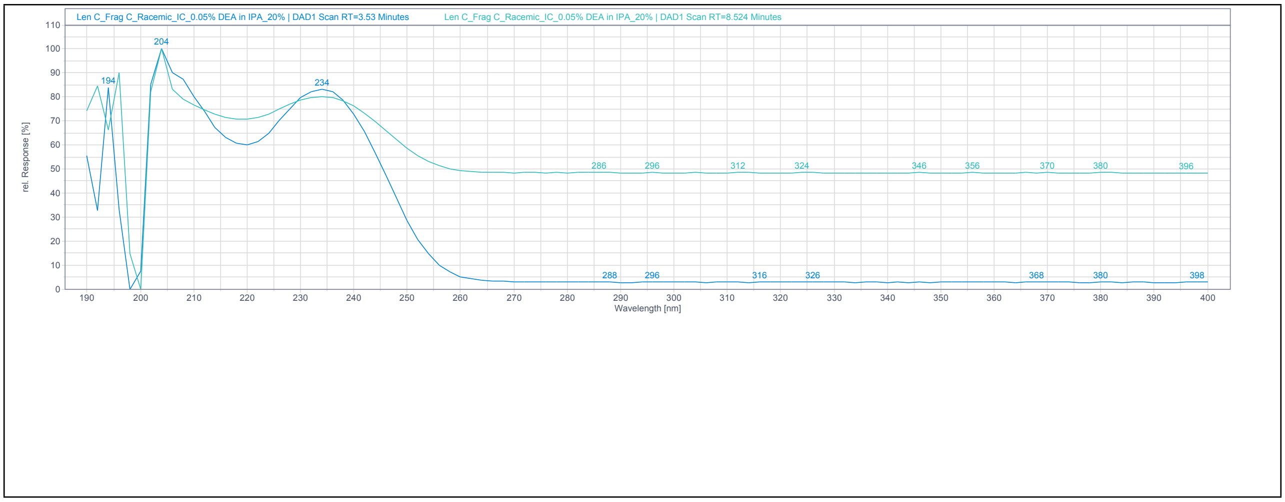

Figure S17. Chiral GC (GC-FID) spectrum of **Frag C**.

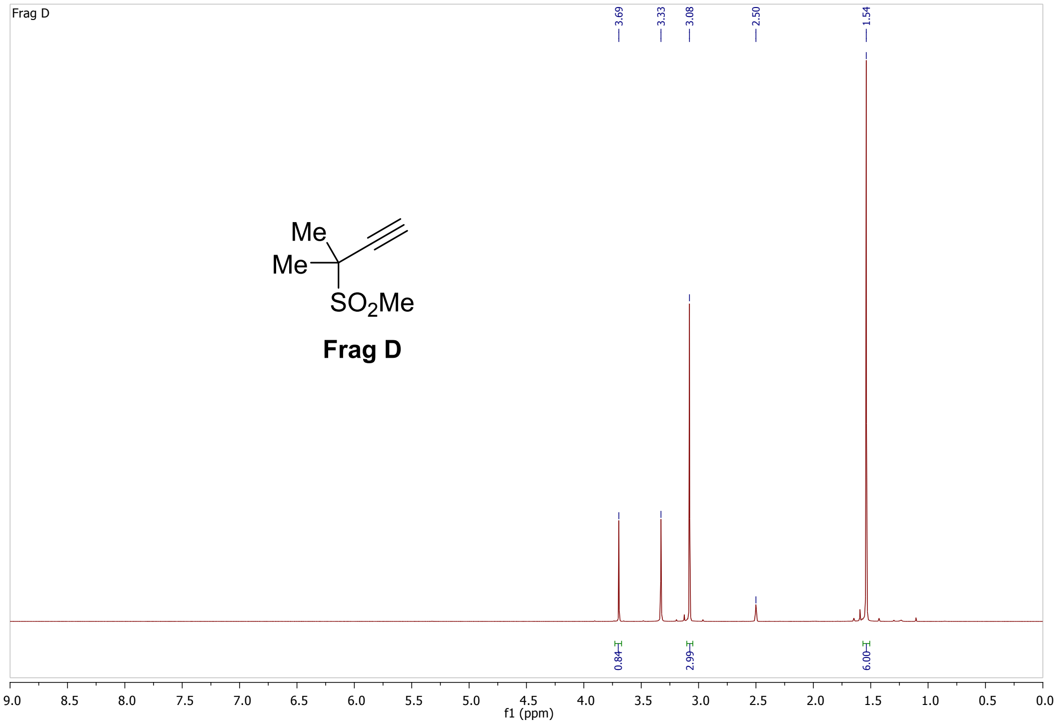

Figure S18. ^{1}H NMR (600 MHz, DMSO-*d6*) of **Frag D**.

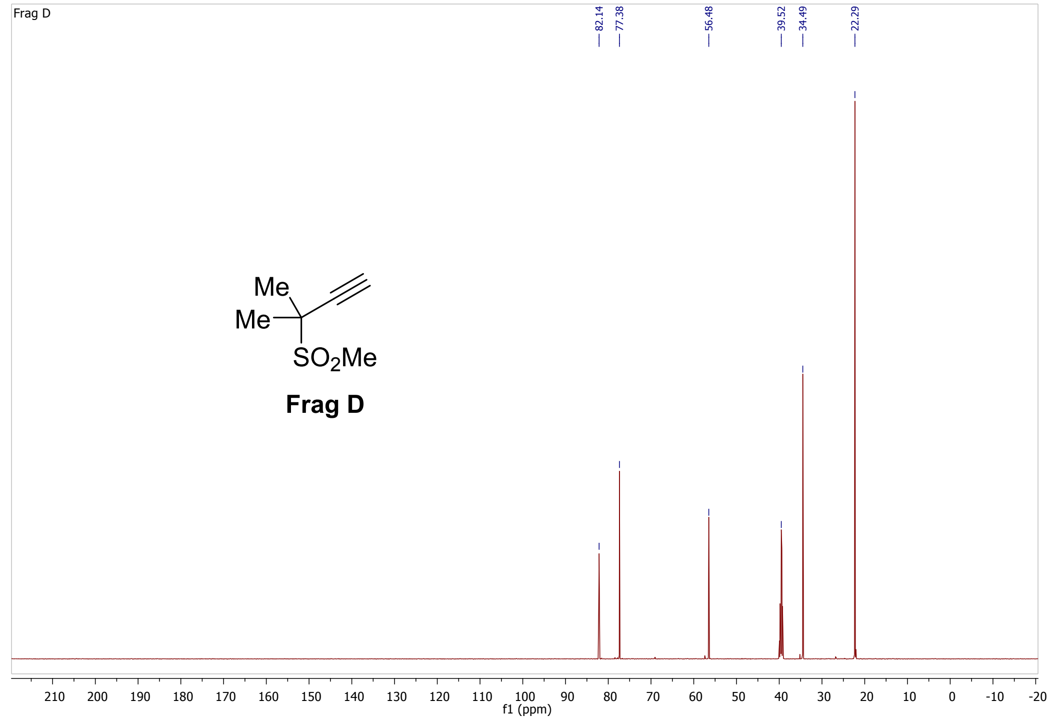

Figure S19. ^{13}C NMR (150 MHz, DMSO-d_6) of **Frag D**.

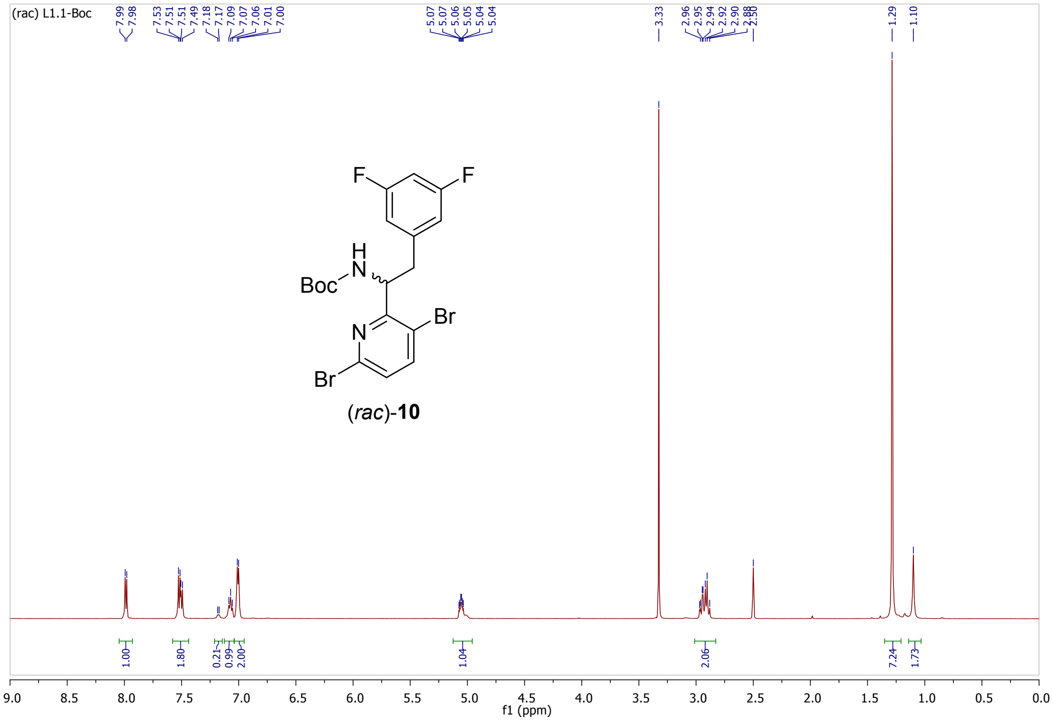

Figure S20. ^{1}H NMR (600 MHz, DMSO-d_6) of (*rac*)-**10.**

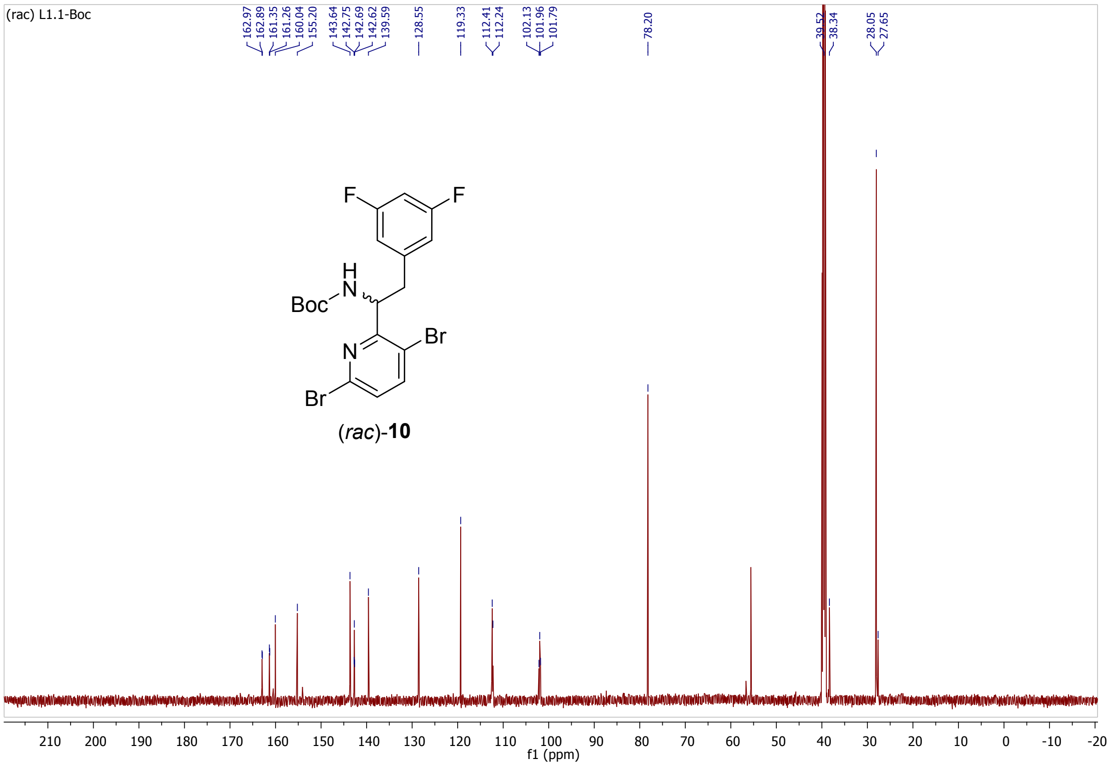

Figure S21. ^{13}C NMR (151 MHz, DMSO-d_6) of (*rac*)-**10.**

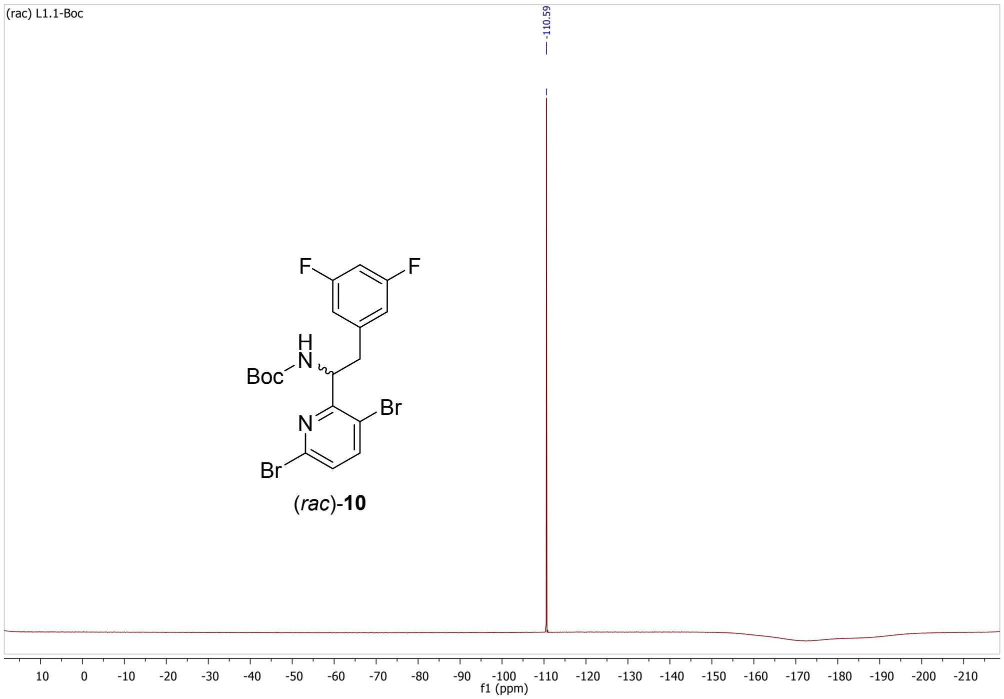

Figure S22. ^{19}F NMR (565 MHz, DMSO-d_6) of (*rac*)-**10.**

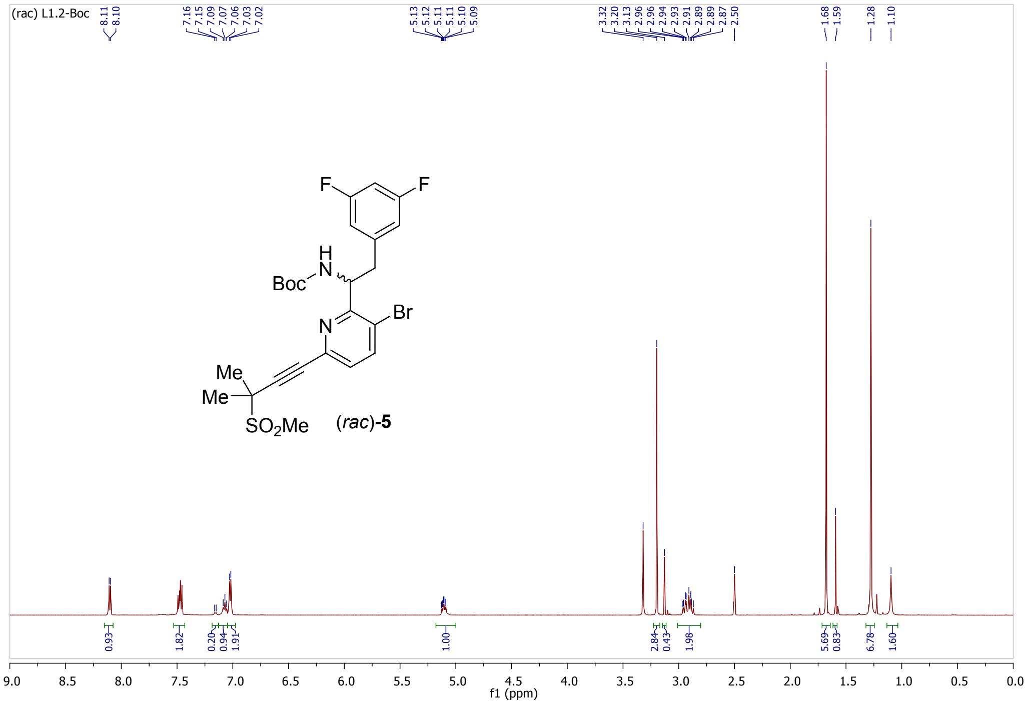

Figure S23. ^{1}H NMR (600 MHz, DMSO-d_6) of (*rac*)-**5.**

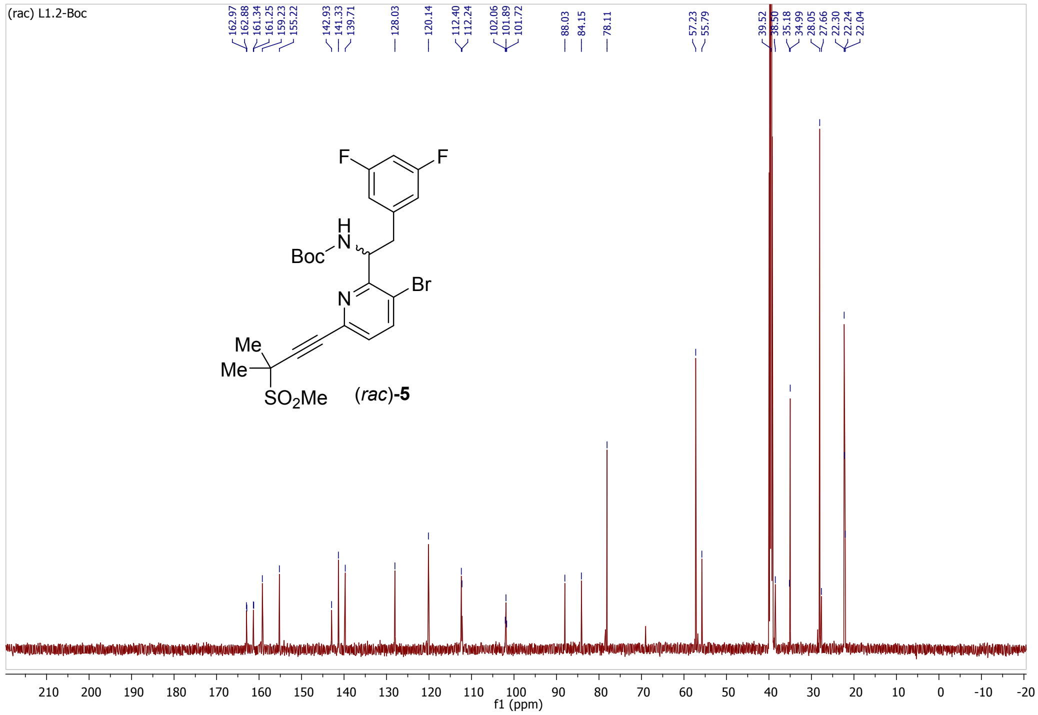

Figure S24. ^{13}C NMR (151 MHz, DMSO-d_6) of (*rac*)-**5.**

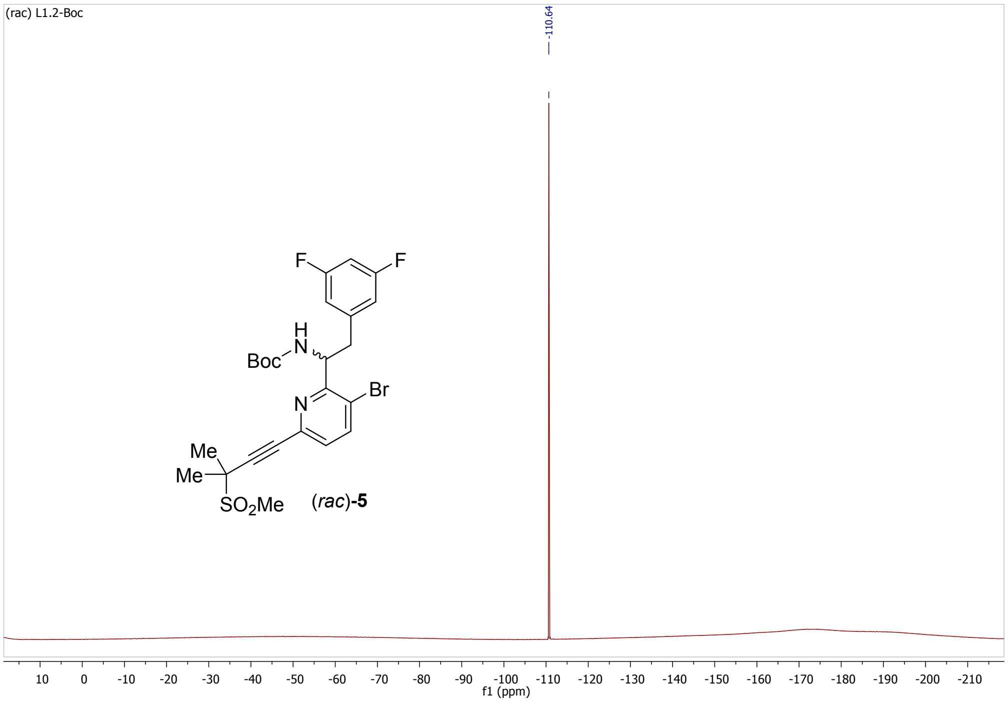

Figure S25. ^{19}F NMR (565 MHz, DMSO-d_6) of (*rac*)-**5.**

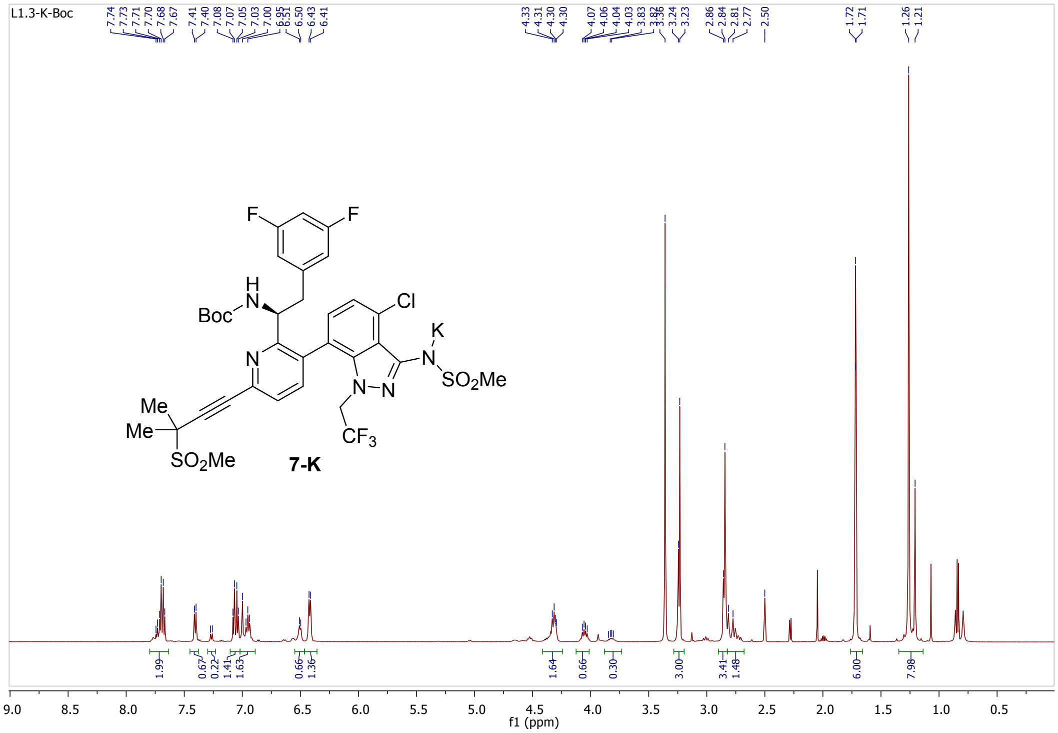

Figure S26. ^{1}H NMR (600 MHz, DMSO-d_6) of **7-K.**

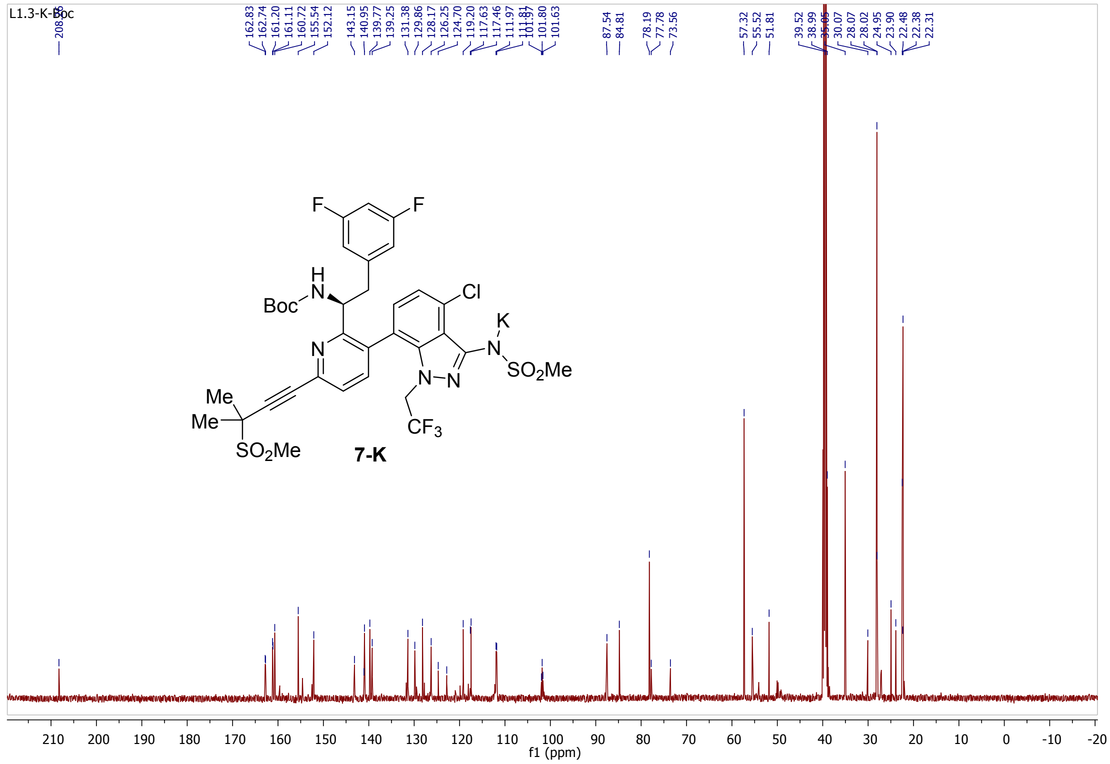

Figure S27. ^{13}C NMR (600 MHz, DMSO-d_6) of **7-K.**

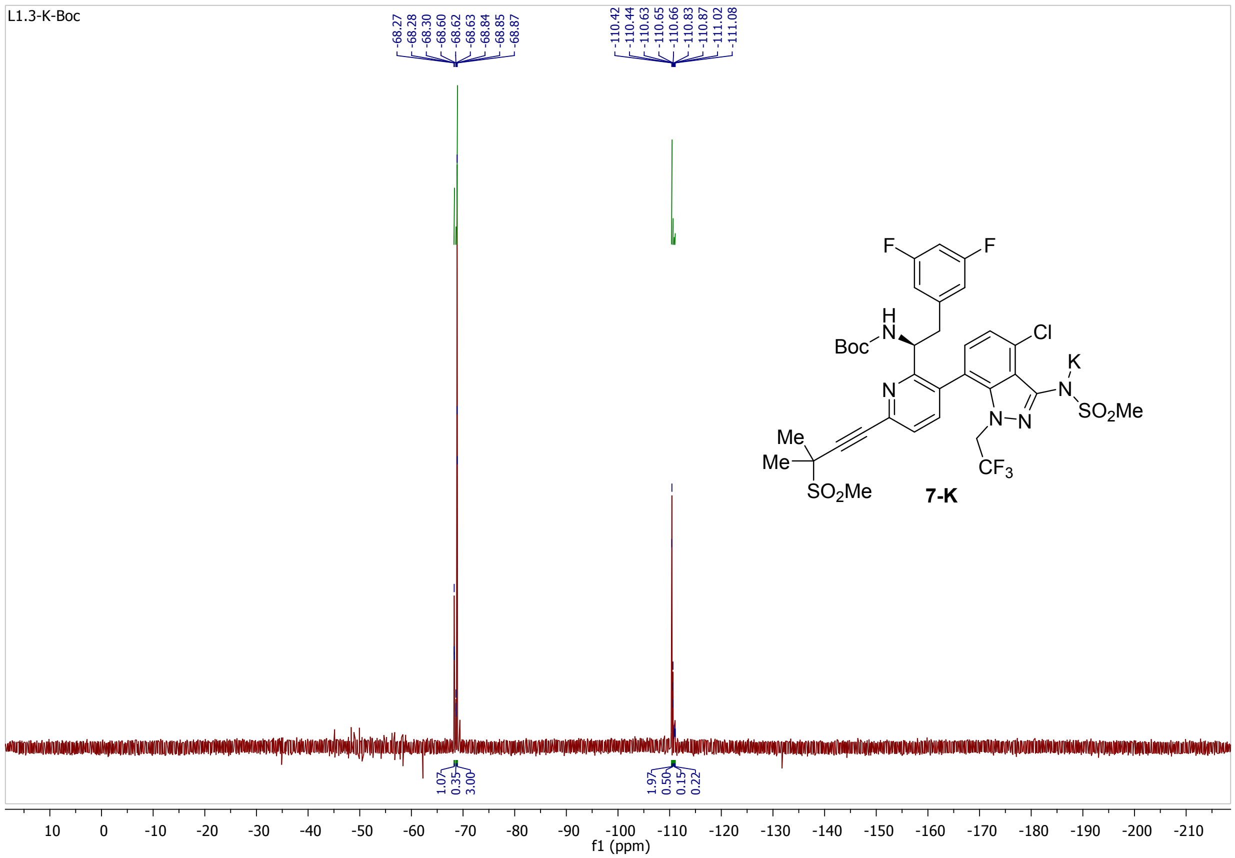

Figure S28. ^{19}F NMR (565 MHz, DMSO-d_6) of **7-K.**

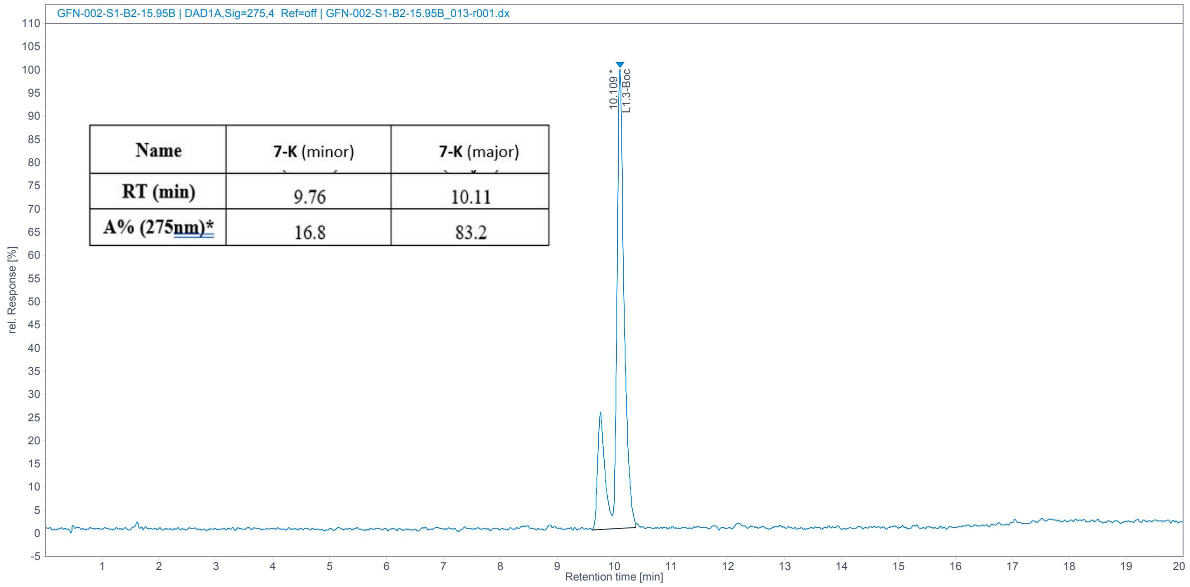

Name	**7-K** (minor)	**7-K** (major)
RT (min)	9.76	10.11
A% (275nm)*	16.8	83.2

Figure S29. HPLC spectrum of **7-K** and atropoisomeric ratio based on A% (275 nm).

GFN-002-S1-B1-15.95 (LC-DAD Wt% and Area % Analysis)

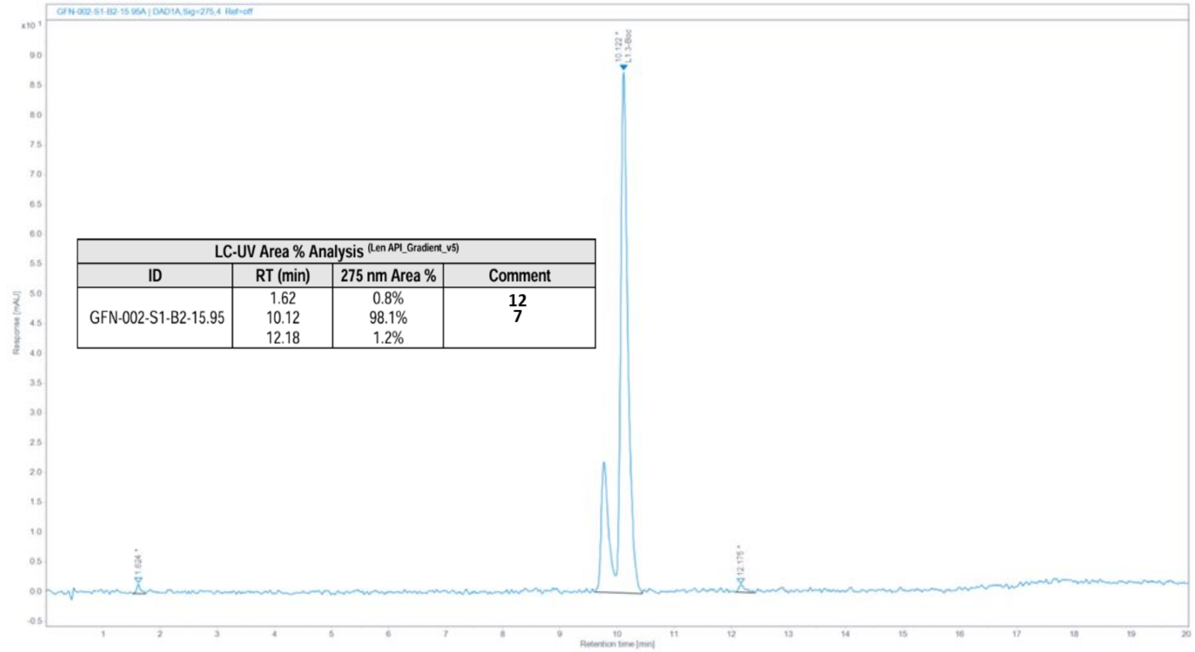

LC-UV Area % Analysis (Len API_Gradient_v5)

ID	RT (min)	275 nm Area %	Comment
GFN-002-S1-B2-15.95	1.62	0.8%	**12**
	10.12	98.1%	**7**
	12.18	1.2%	

Figure S30. LC-UV area% analysis of **7-K** (275 nm).

GFN-002-S1-B1-15.95 (LC-MS Impurity Analysis)

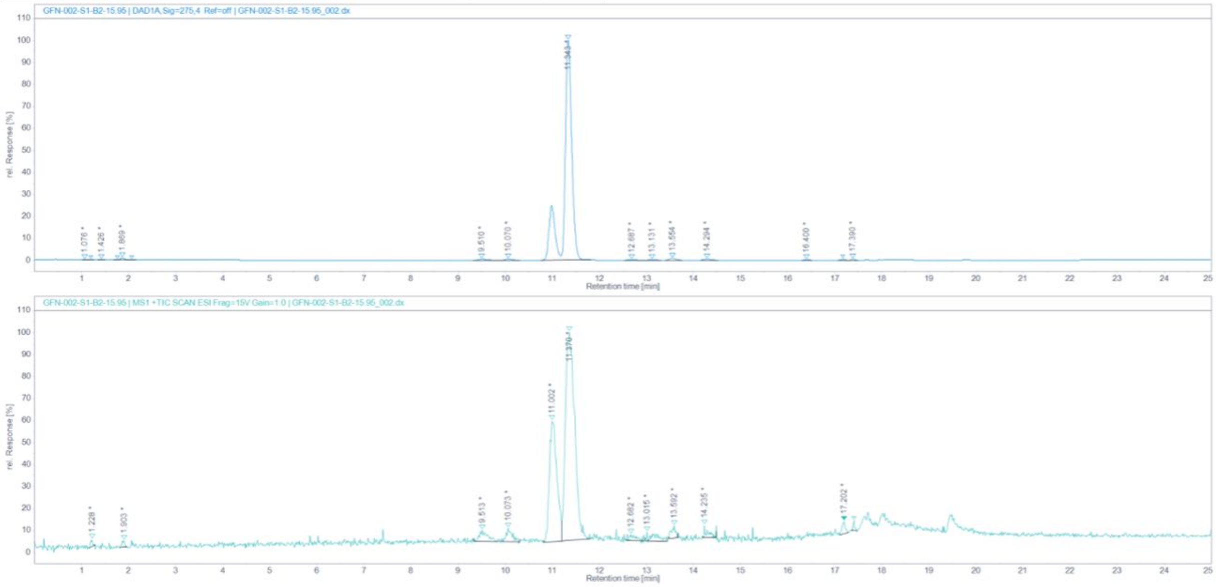

Impurity Analysis by LC-MS (LCMS A_LenAPI_MS)				
**ID**	**RT (min)**	**275 nm Area %**	***m/z***	**Comment**
GFN-002-S1-B2 15.95	1.08	0.01%	No Ionization	
	1.20	0.01%	308 (ESI+)	
	1.43	0.02%	No Ionization	
	1.77	0.03%	No Ionization	
	1.87	0.44%	328 (M+H)	**12**
	2.07	0.01%	279 (ESI+)	
	9.51	0.42%	623 (M+H)	**11**
	10.07	0.40%	770 (ESI+); 714 (M+H-Boc)	
	11.34	96.85%	804 (M+H)	**7**

Impurity Analysis by LC-MS (LCMS A_LenAPI_MS)				
**ID**	**RT (min)**	**275 nm Area %**	***m/z***	**Comment**
	12.69	0.23%	1075 (ESI+)	Contains 2-3 Cl atoms
	13.13	0.18%	1095 (ESI+)	Contains 1 Cl atom
	13.55	0.66%	1095 (ESI+)	Contains 1 Cl atom
	14.29	0.37%	1095 (ESI+)	Contains 1 Cl atom
	16.40	0.10%	No Ionization	
	17.18	0.13%	725 (ESI+); 668 (M+H-Boc)	Contains 1 Cl atom
	17.39	0.12%	995 (ESI+)	Contains 2 Cl atoms

Figure S31. Impurity analysis of **7-K** by LC-MS.

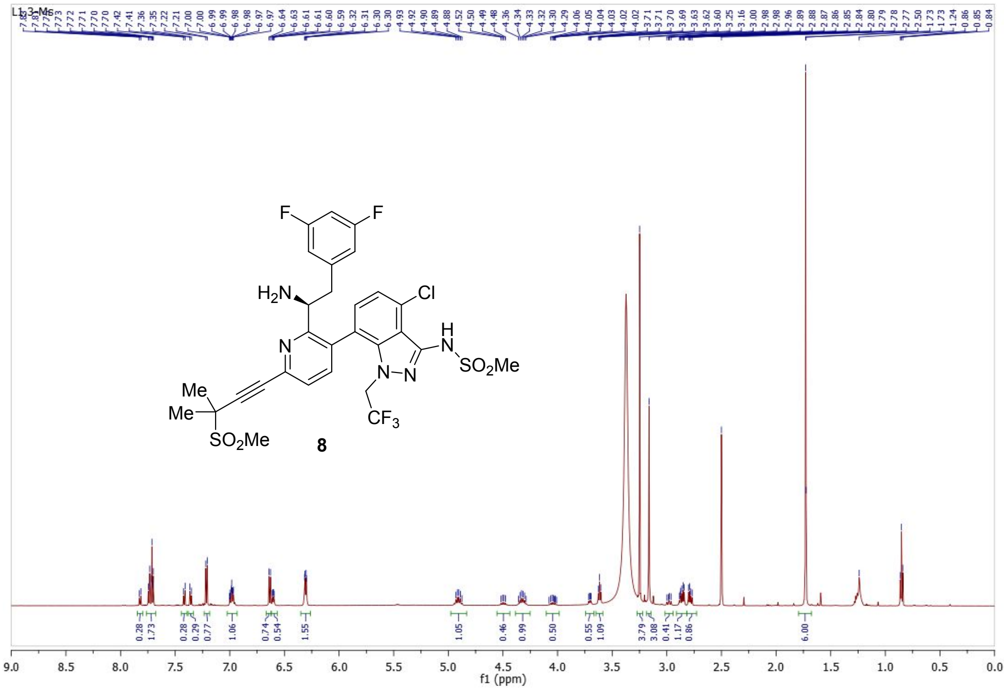

Figure S32. ^{1}H NMR (600 MHz, DMSO-d_6) of **8.**

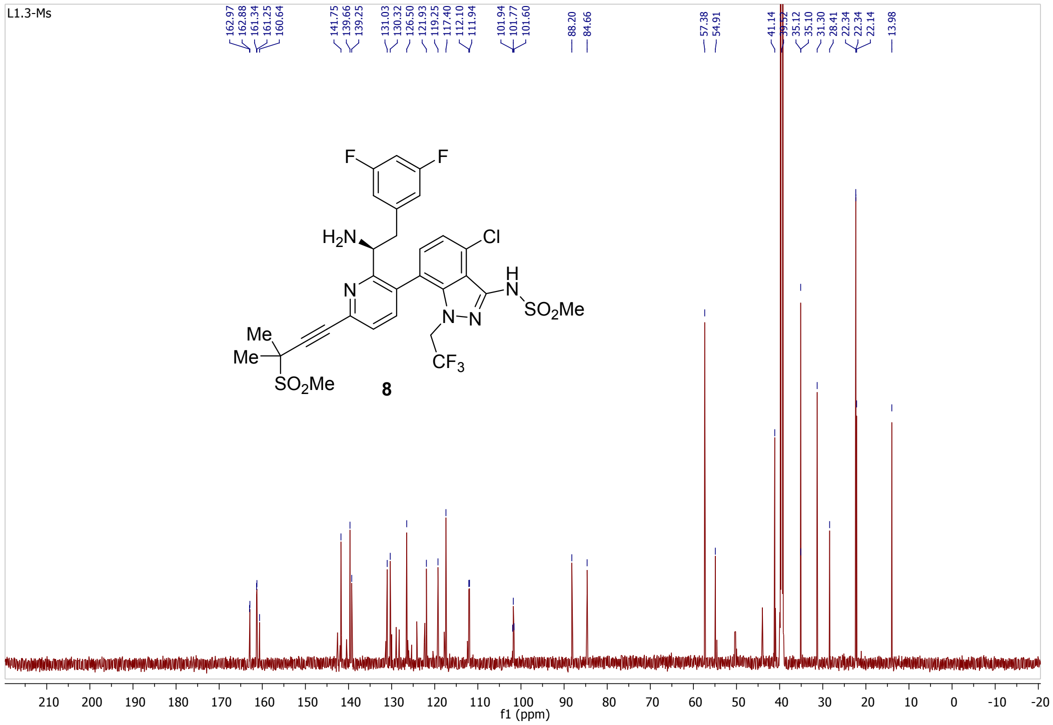

Figure S33. ^{13}C NMR (600 MHz, DMSO-d_6) of **8.**

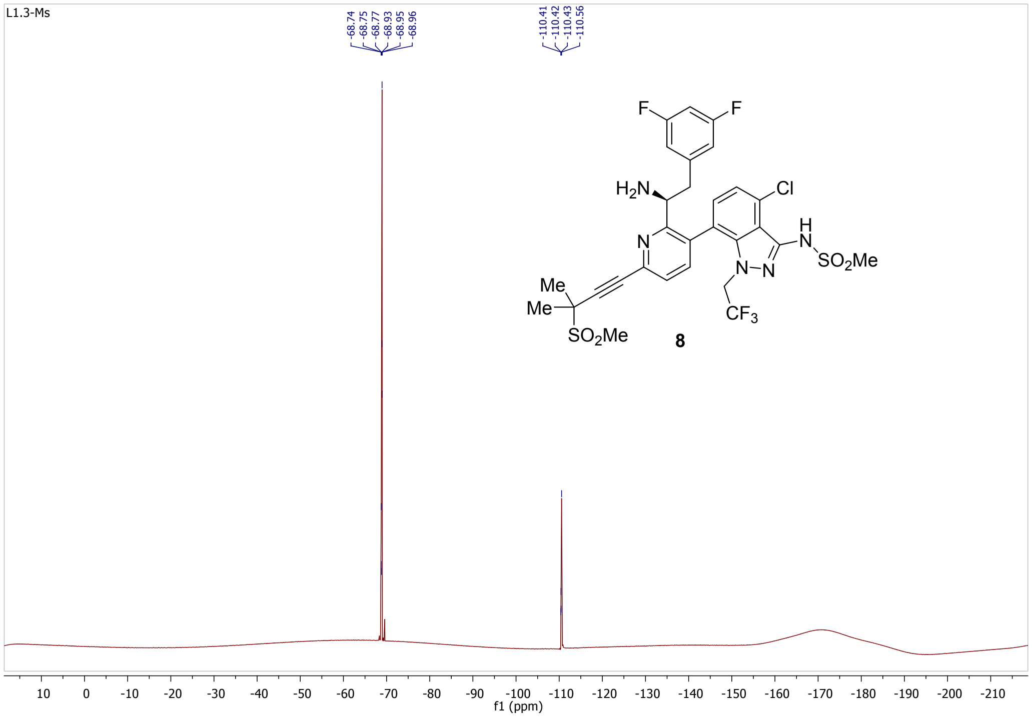

Figure S34. ^{19}F NMR (565 MHz, DMSO-d_6) of **8.**

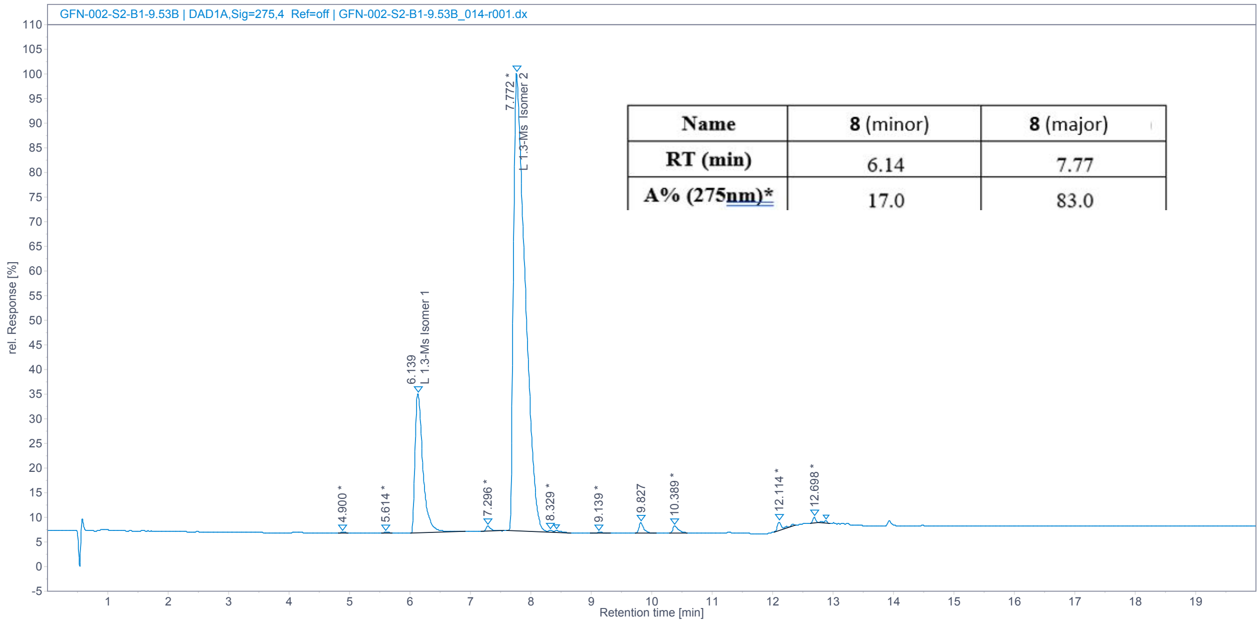

Name	**8** (minor)	**8** (major)
**RT (min)**	6.14	7.77
**A% (275nm)***	17.0	83.0

Figure S35. HPLC spectrum of **8** and atropoisomeric ratio based on A% (275 nm).

GFN-002-S2-B1-9.53 (LC-UV Wt% and Area % Analysis)

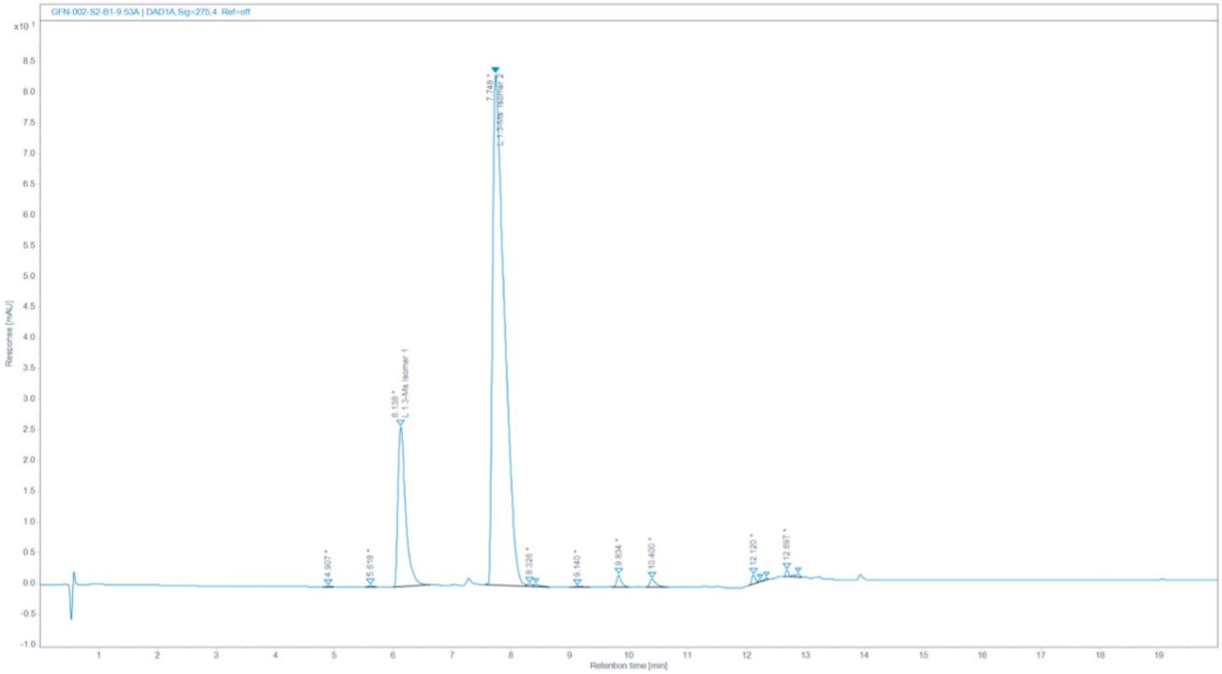

LC-UV Area % Analysis (LCUV_L13Ms)			
ID	RT (min)	275 nm Area %	Comment
GFN-002-S2-B1-9.53	4.91	0.04%	
	5.62	0.1%	
	6.14	16.5%	**8** (minor isomer)
	7.75	80.5%	**8** (major isomer)
	8.33	0.1%	
	8.43	0.2%	
	9.14	0.1%	
	9.83	0.7%	
	10.4	0.7%	
	12.12	0.5%	
	12.24	0.1%	
	12.34	0.1%	
	12.7	0.2%	
	12.89	0.2%	

Figure S36. LC-UV (275 nm) A% analysis of **8**.

GFN-002-S2-B1-9.53 (LC-MS Impurity Analysis)

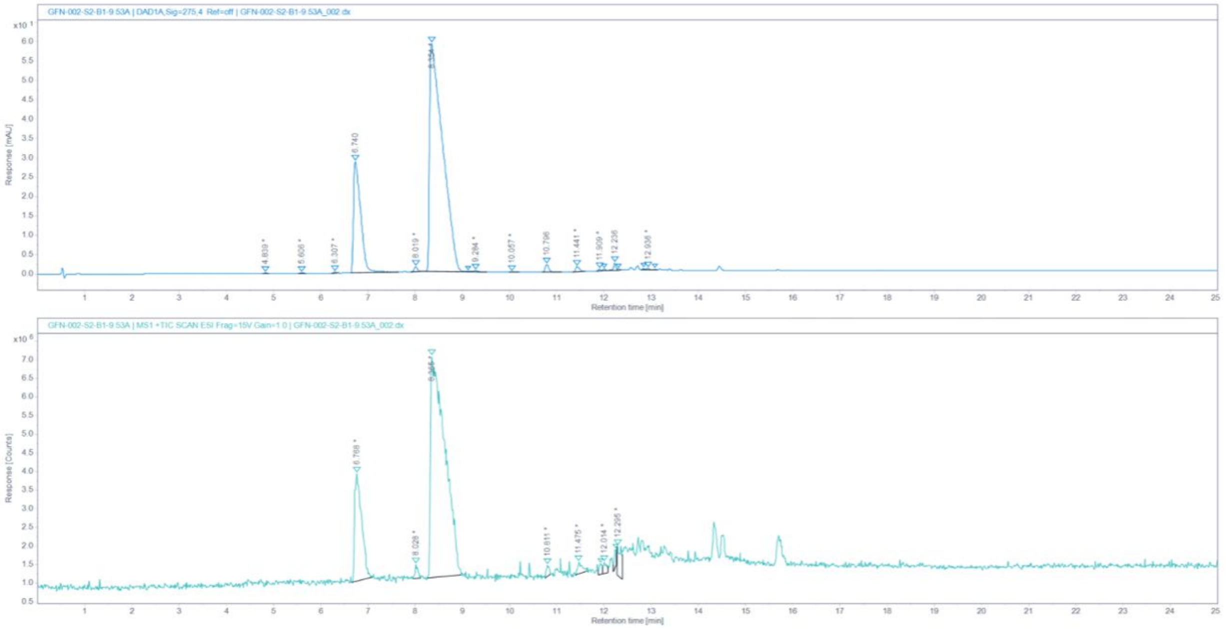

LC-MS Impurity Analysis (LCMS A_LenAPI_MS)				
**ID**	**RT (min)**	**275 nm Area %**	***m/z***	**Comment**
GFN-002-S2-B1-9.53	4.84	0.03%	No Ionization	
	5.61	0.04%	No Ionization	
	6.31	0.1%	No Ionization	
	6.74	20.3%	704 (M+H)	**8** (minor isomer)
	8.02	0.4%	670 (ESI+)	
	8.35	76.6%	704 (M+H)	**8** (major isomer)
	9.13	0.2%	No Ionization	
	9.28	0.2%	No Ionization	
	10.06	0.1%	No Ionization	
	10.80	0.8%	995 (ESI+)	Contains 2 Cl atoms
	11.44	0.6%	995 (ESI+)	Contains 2 Cl atoms
		Co-elutes	624 (ESI+)	Contains 2 Cl atoms
	11.91	0.1%	732 (ESI+)	
	12.01	0.1%	No Ionization	
	12.24	0.4%	732 (ESI+)	
	12.31	0.1%	No Ionization	
	12.84	0.1%	No Ionization	
	12.94	0.1%	No Ionization	
	13.09	0.04%	No Ionization	

Figure S37. Impurity analysis of **8** by LC-MS.

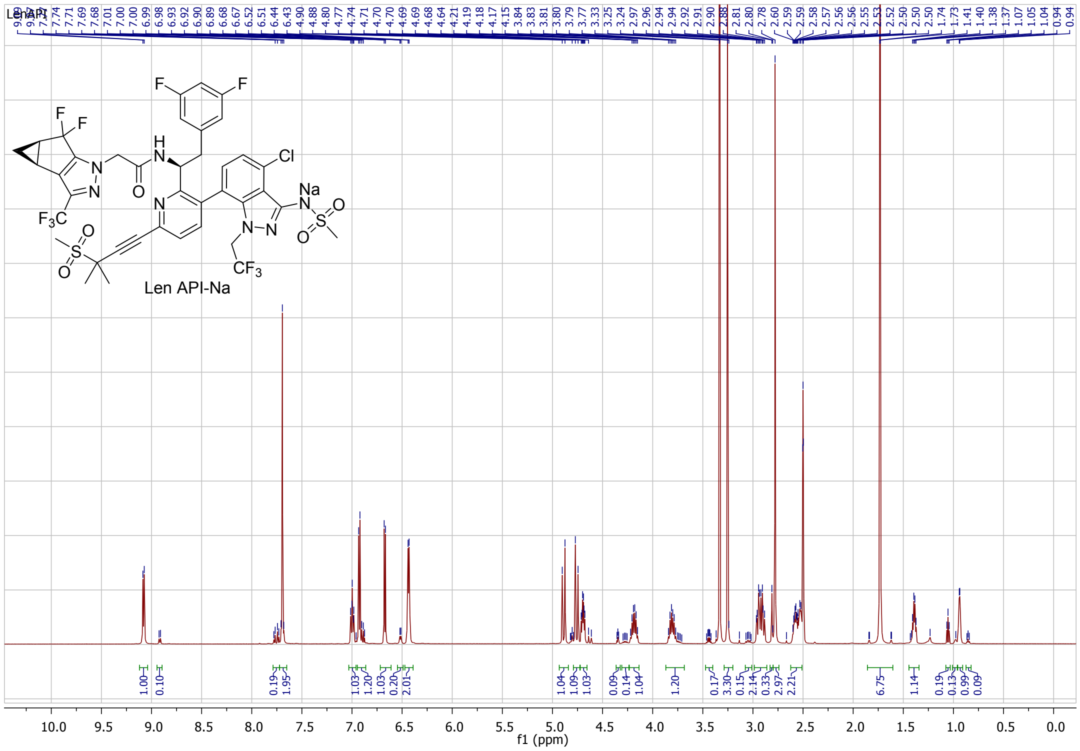

Figure S38. ^{1}H NMR (600 MHz, DMSO-d_6) of **Len-API-Na.**

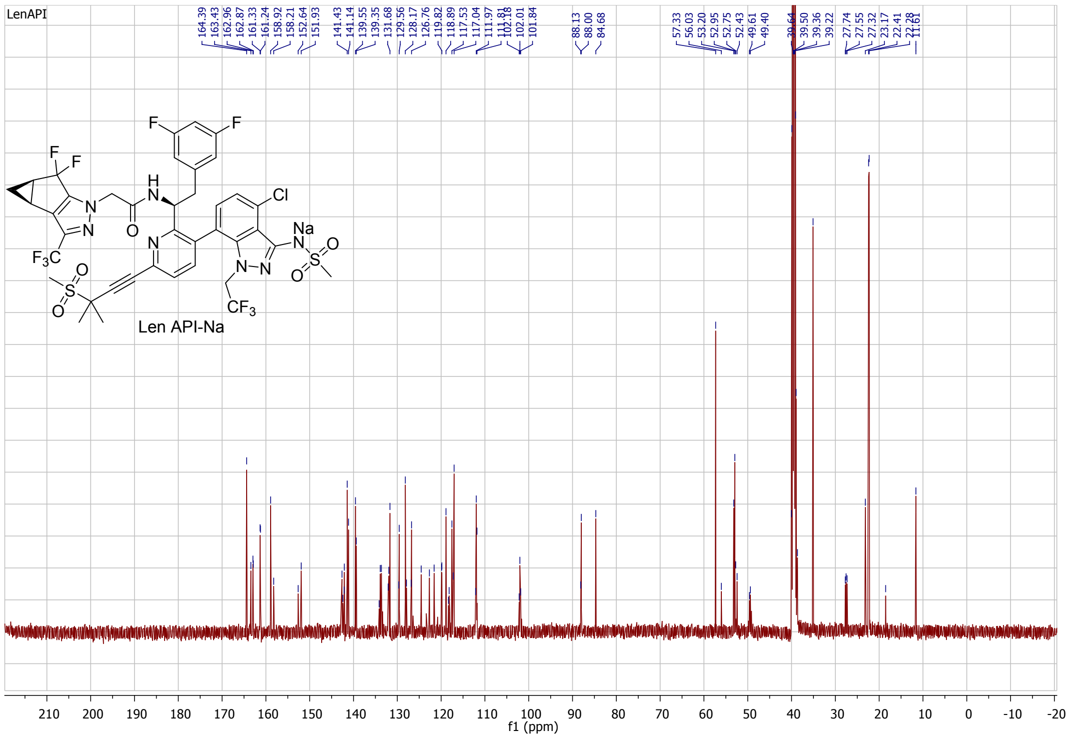

Figure S39. ^{13}C NMR (151 MHz, DMSO-d_6) of **Len-API-Na.**

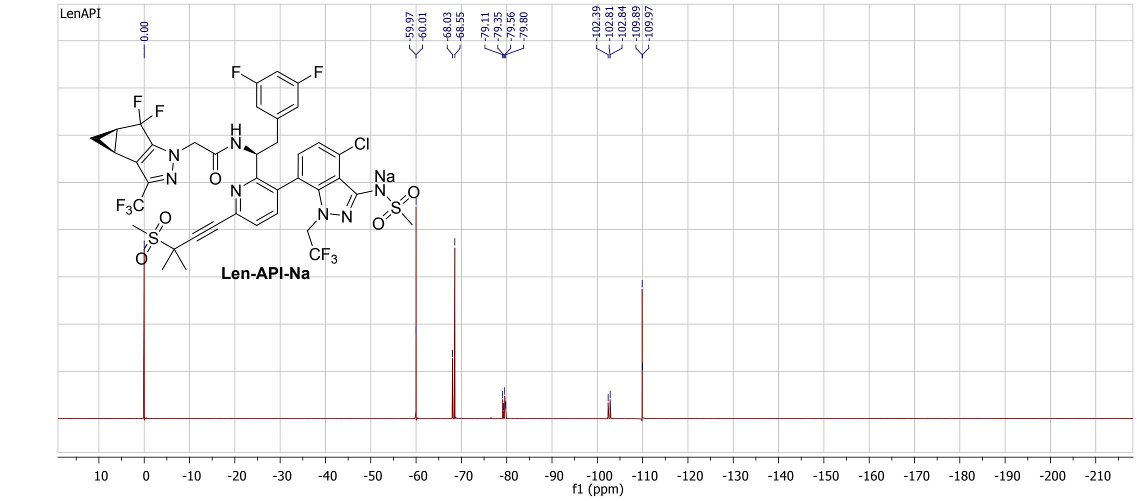

Figure S40. ^{19}F NMR (565 MHz, DMSO-d_6) of **Len-API-Na.**

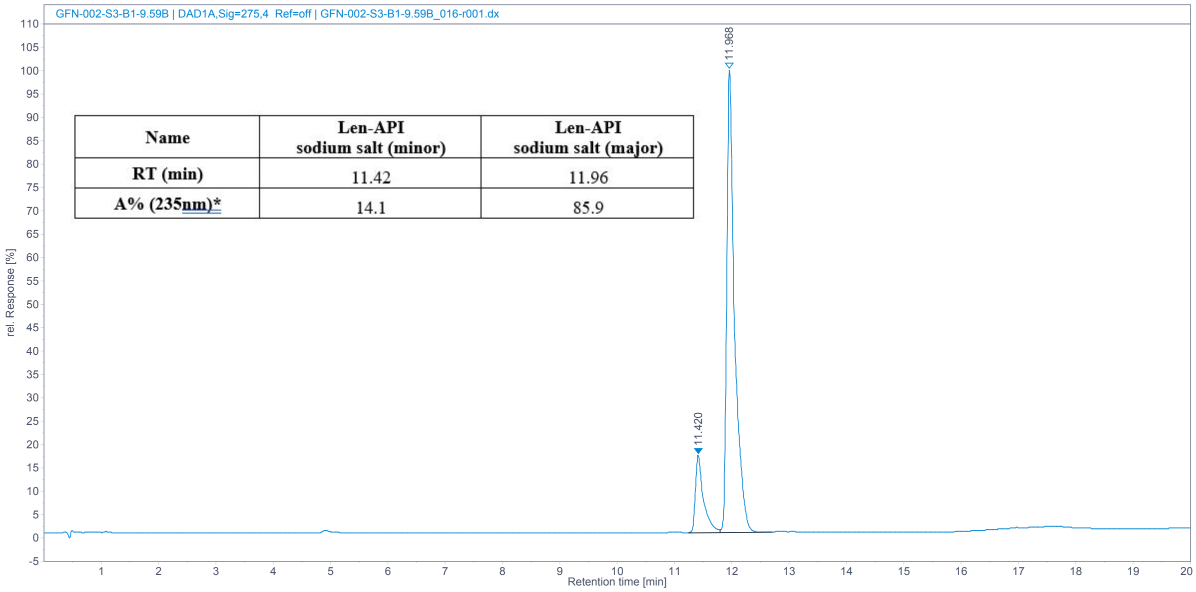

Name	Len-API sodium salt (minor)	Len-API sodium salt (major)
RT (min)	11.42	11.96
A% (235nm)*	14.1	85.9

Figure S41. HPLC spectrum of **Len-API-Na (sodium salt)** and atropoisomeric ratio based on A% (235 nm).

GFN-002-S3-B0-9.59 (LC-UV Wt% and Area % Analysis)

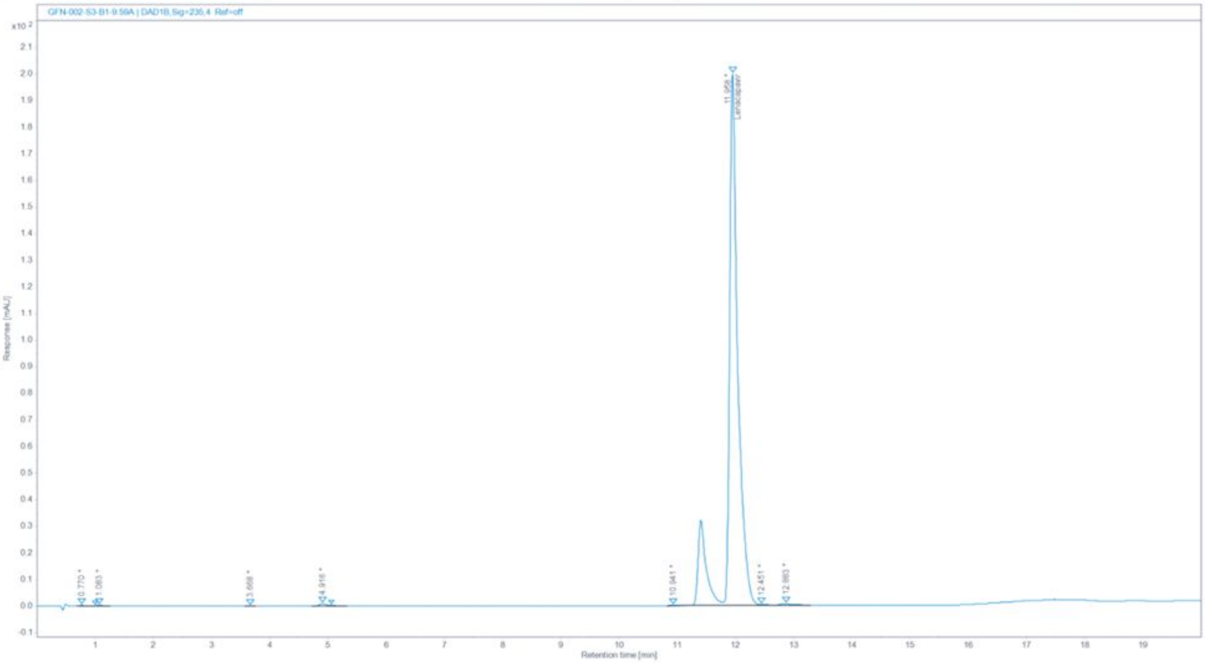

LC-UV Area % Analysis (LCUV_Len API_Gradient)			
ID	RT (min)	275nm Area %	Comment
GFN-002-S3-B1-9.59	0.77	0.1%	L 1.3-Ms Isomer
	1.01	0.01%	
	1.08	0.1%	L 1.3-Ms Isomer
	3.67	0.02%	
	4.92	0.4%	
	5.06	0.2%	L1.3-Ms-Ac Isomer
	10.94	0.1%	
	11.96	98.8%	Len API
	12.45	0.1%	
	12.88	0.4%	

Figure S42. LC-UV (235 nm) A% analysis of **Len-API-Na**.

GFN-002-S3-B0-9.59 (LC-MS Impurity Analysis)

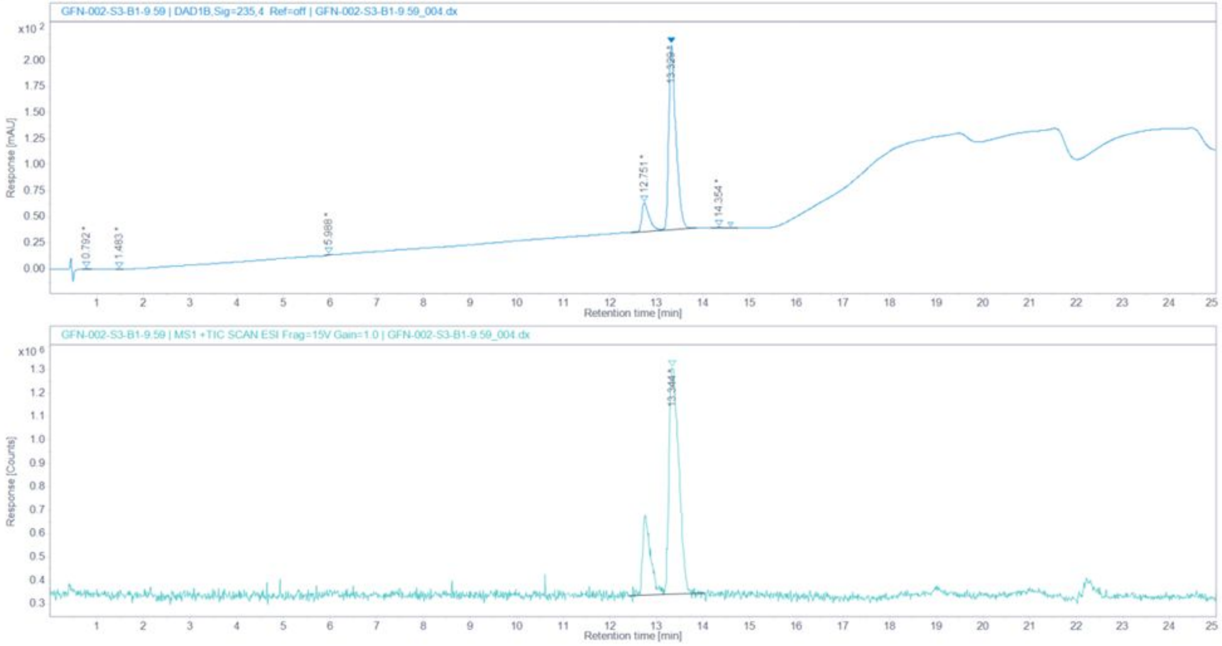

Impurity Analysis by LC-MS (LCMS A_LenAPI_MS)				
**ID**	**RT (min)**	**235 nm Area %**	***m/z***	**Comment**
GFN-002-S3-B1-9.59	0.79	0.2%	No Ionization	
	1.48	0.1%	No Ionization	
	5.99	0.2%	No Ionization	
	12.75	13.7%	968 (M+H)	Len API Isomer
	13.33	85.6%	968 (M+H)	Len API Isomer
	14.35	0.3%	No Ionization	
	14.6	0.1%	No Ionization	

Figure S43. Impurity analysis of **Len-API-Na** by LC-MS.

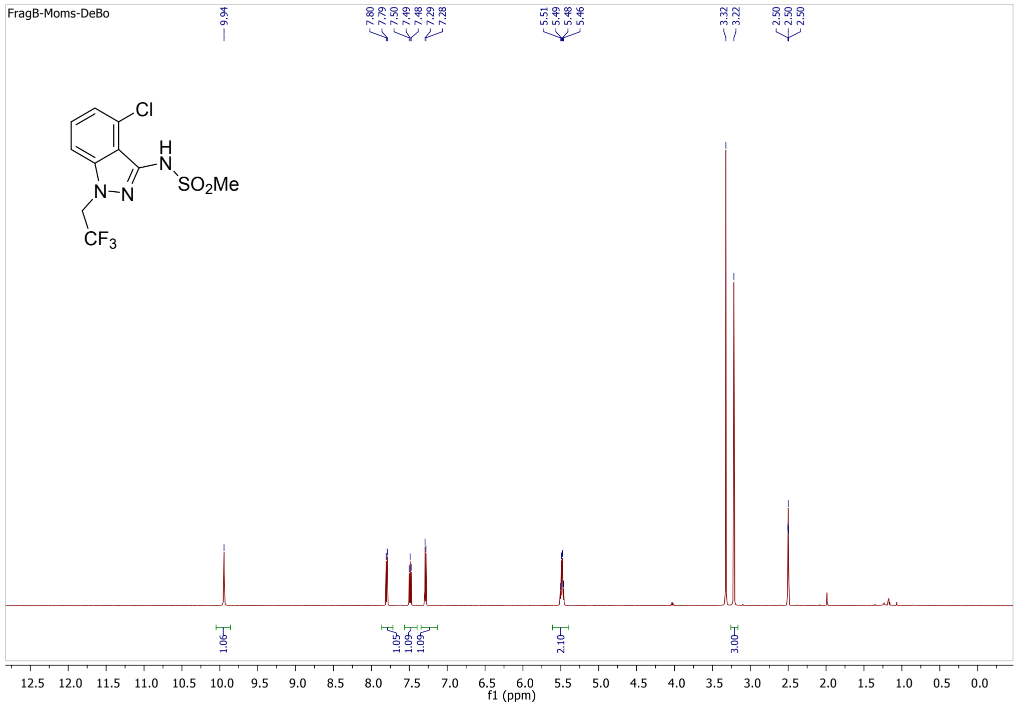

Figure S44. ^{1}HNMR (600MHz, DMSO-d$_6$) of **12.**

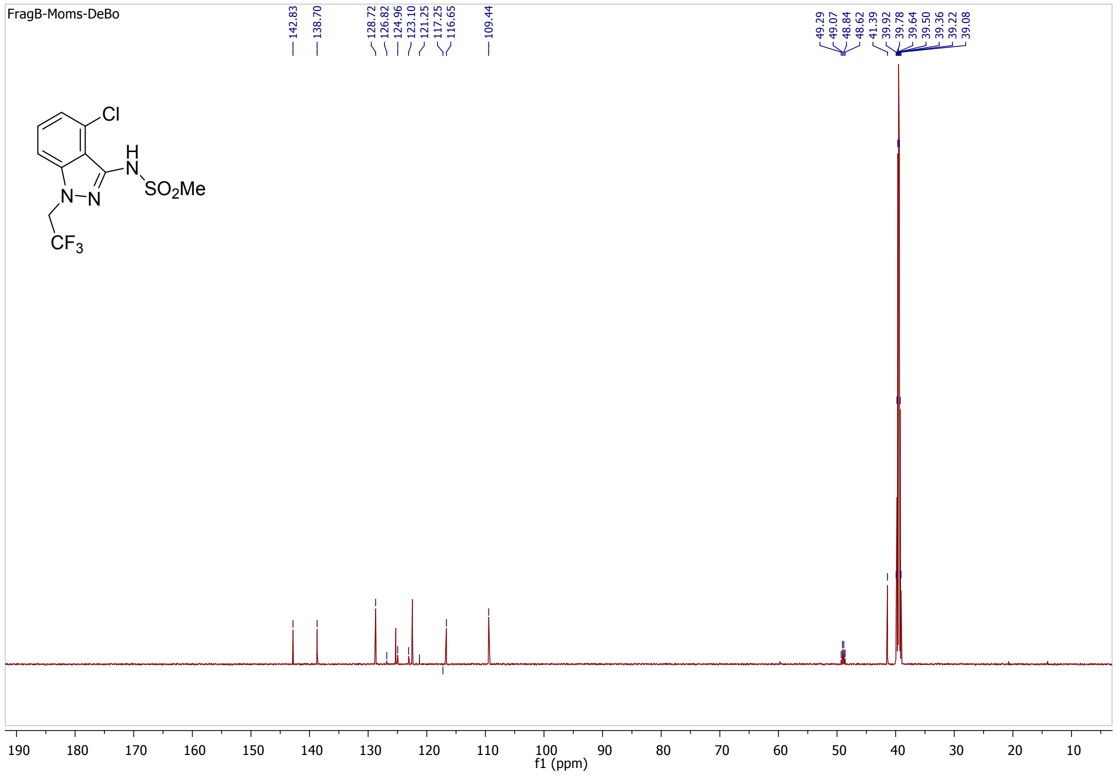

Figure S45. ^{13}CNMR (151MHz, DMSO-d$_6$) of **12**.

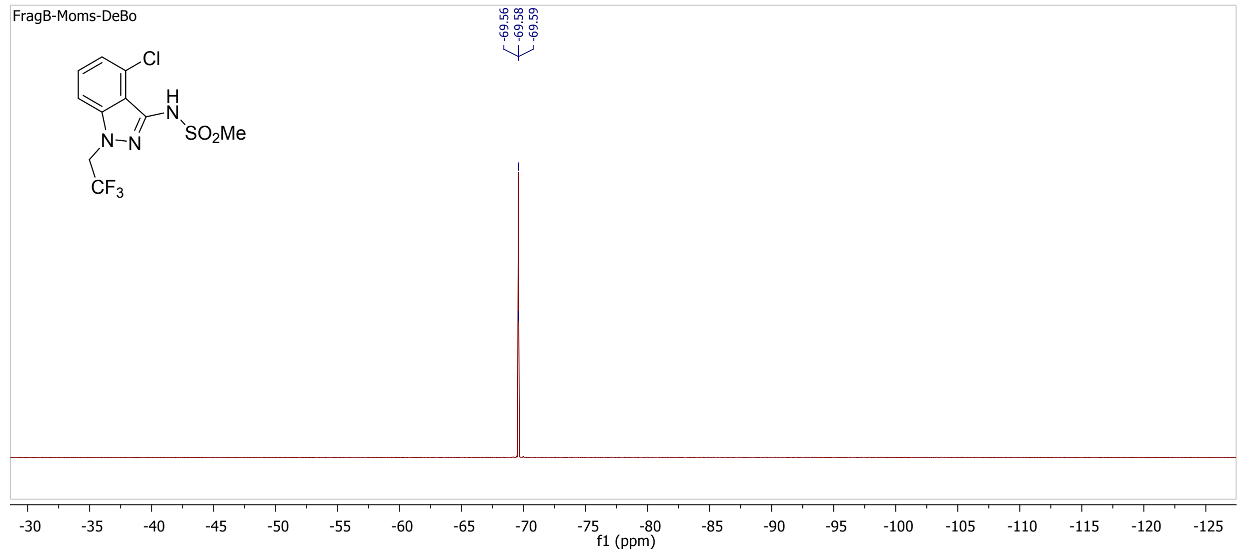

Figure S46. ^{19}F (565MHz, DMSO-d$_6$) of **12**.

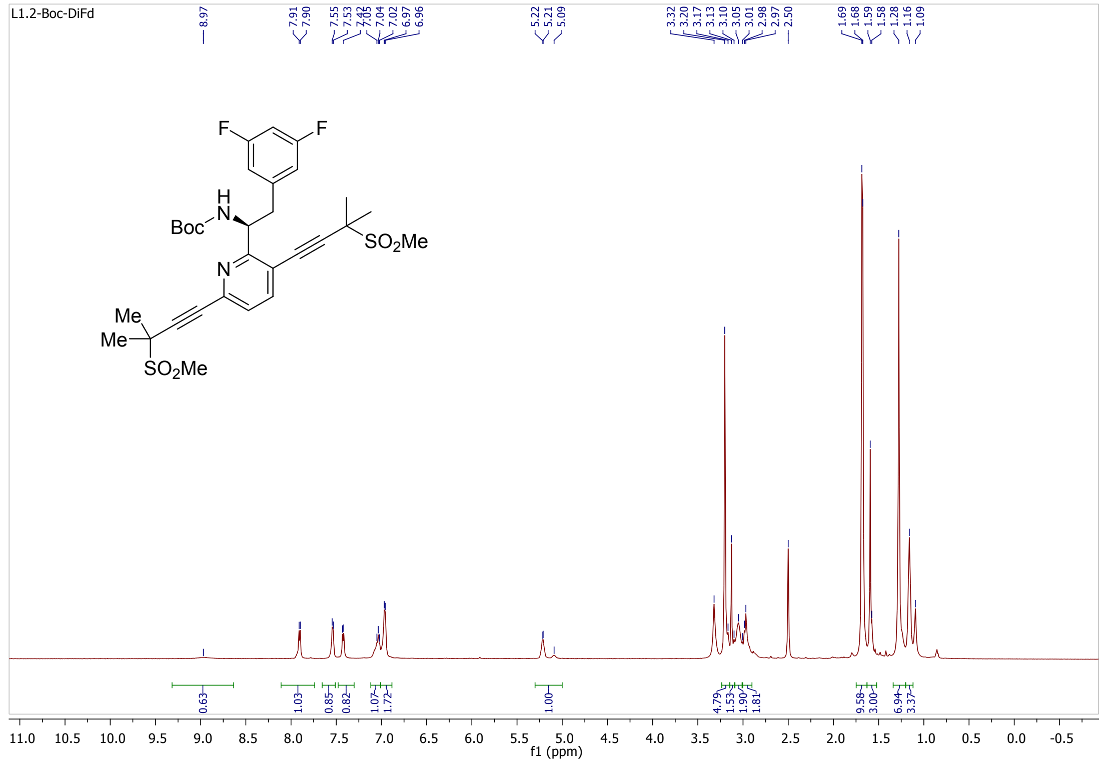

Figure S47. ^{1}H (600MHz, DMSO-d$_6$) of **11.**

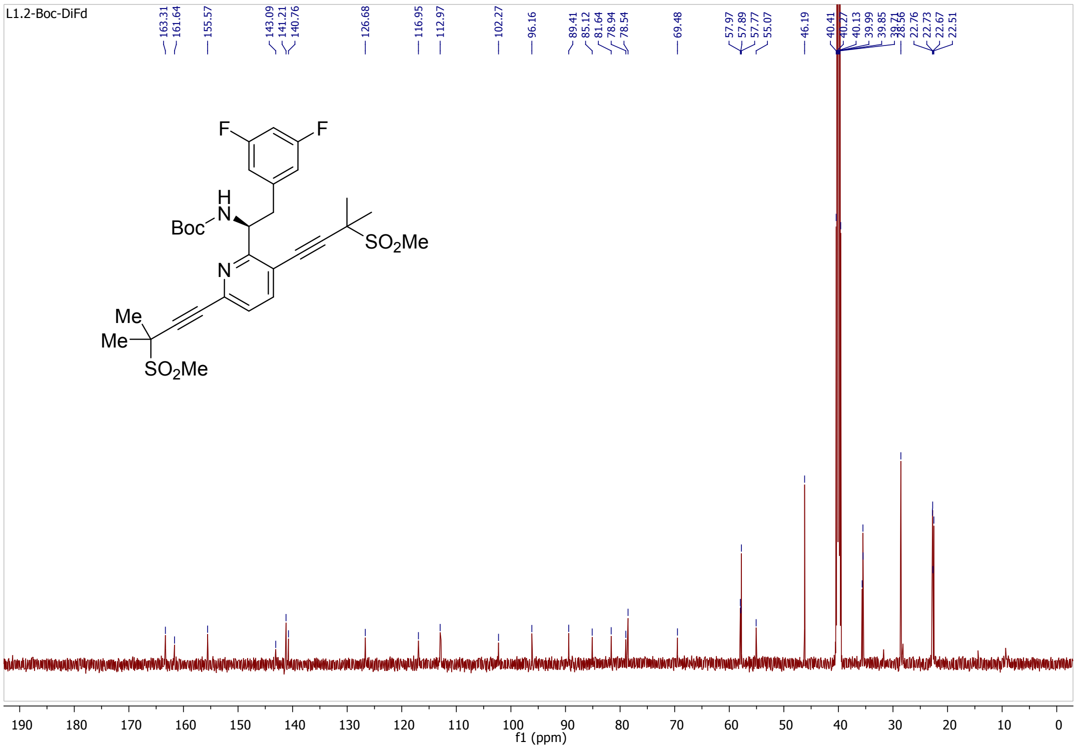

Figure S48. ^{13}C (151MHz, DMSO-d_6) of **11.**

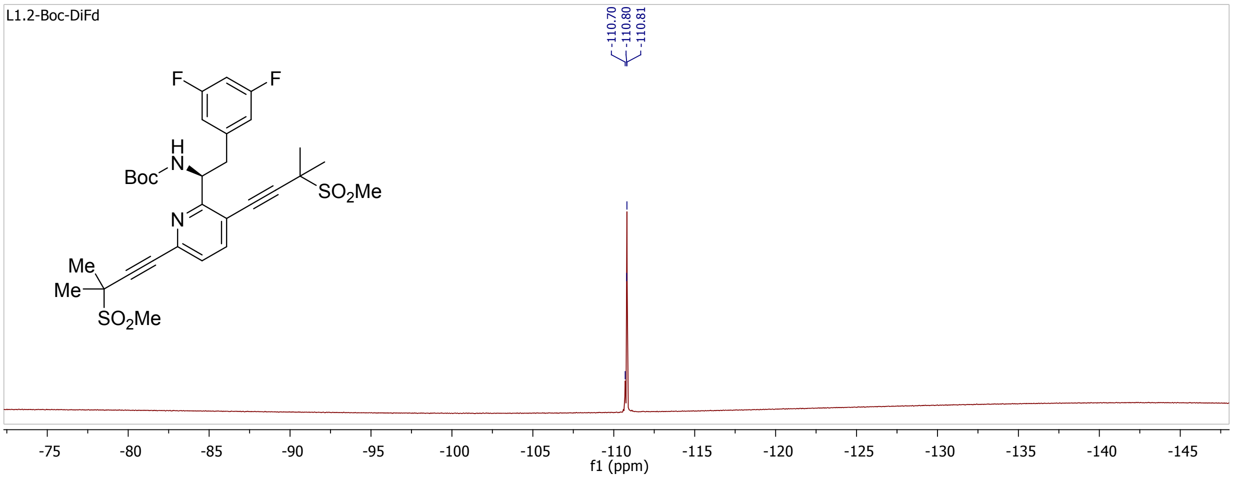

Figure S49. ^{19}F (565MHz, DMSO-d_6) of **11.**

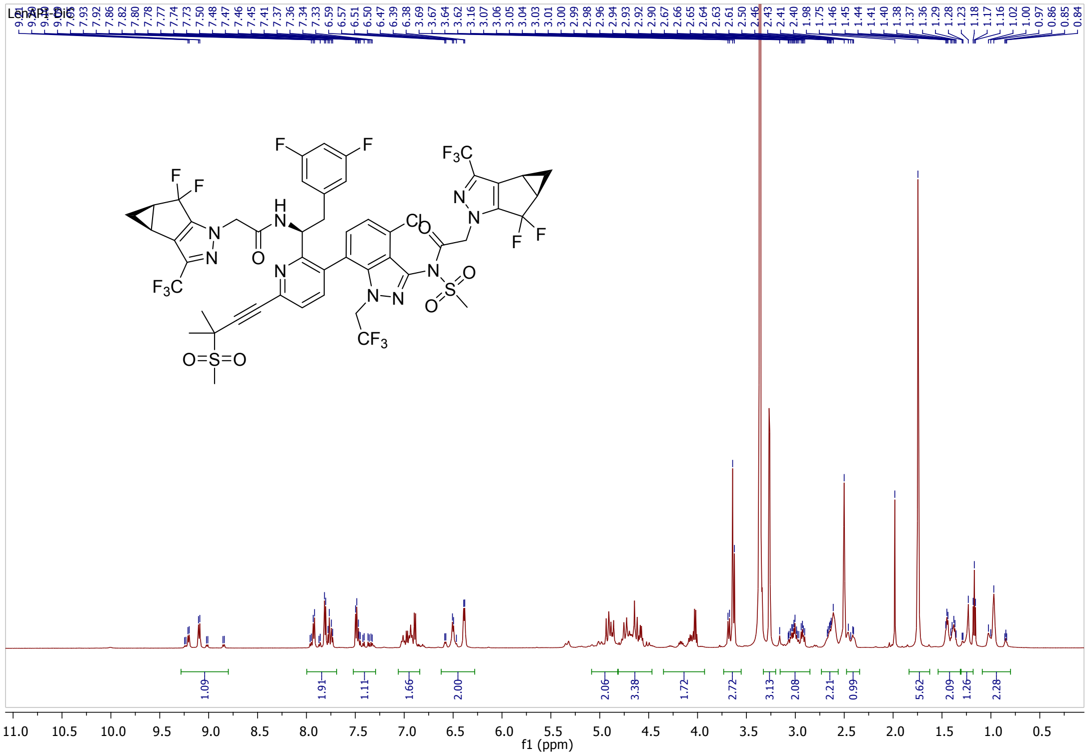

Figure S50. ^{1}H NMR (600MHz, DMSO-d_6) of **13.**

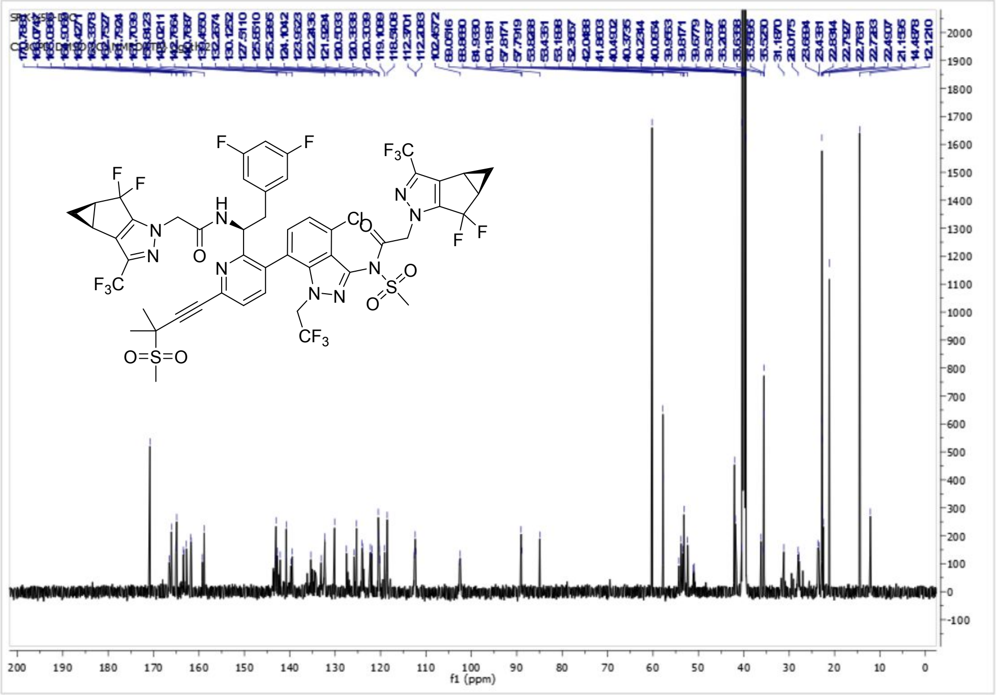

Figure S51. ^{13}C NMR (151MHz, DMSO-d_6) of **13.**

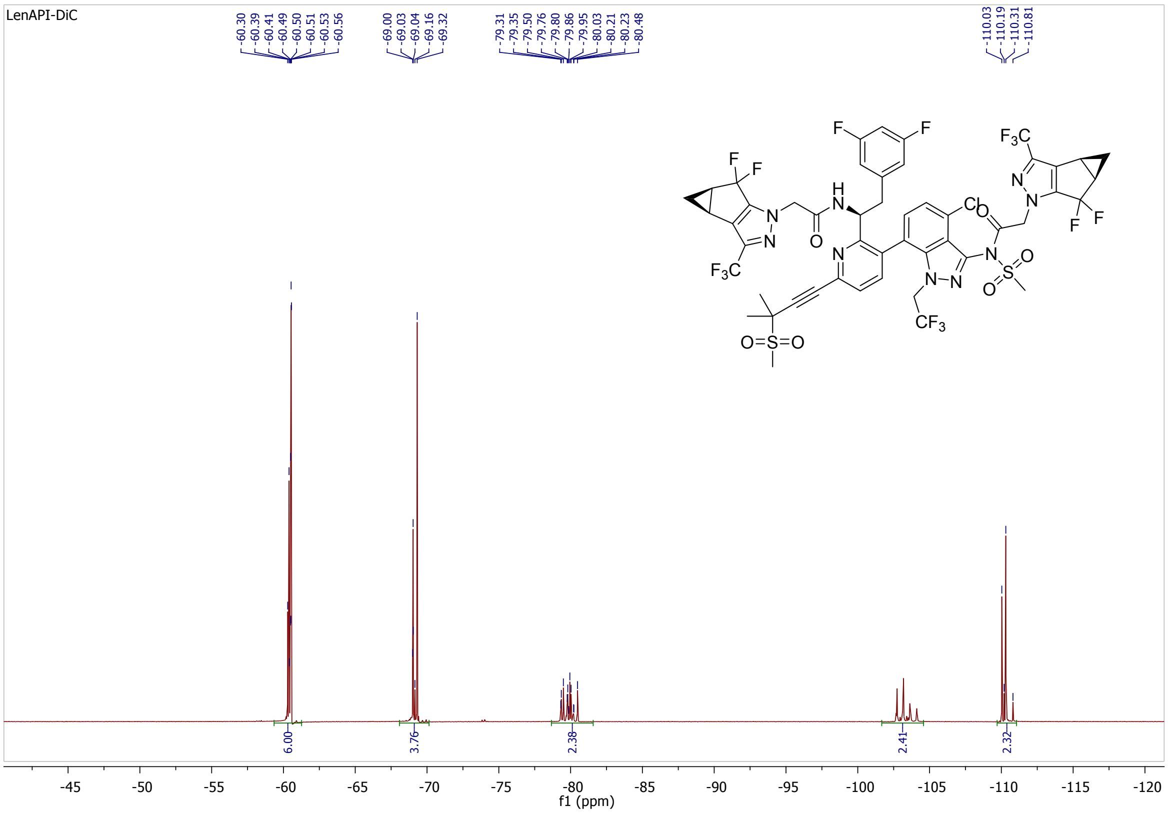

Figure S52. ^{19}F NMR (565MHz, DMSO-d$_6$) of **13.**

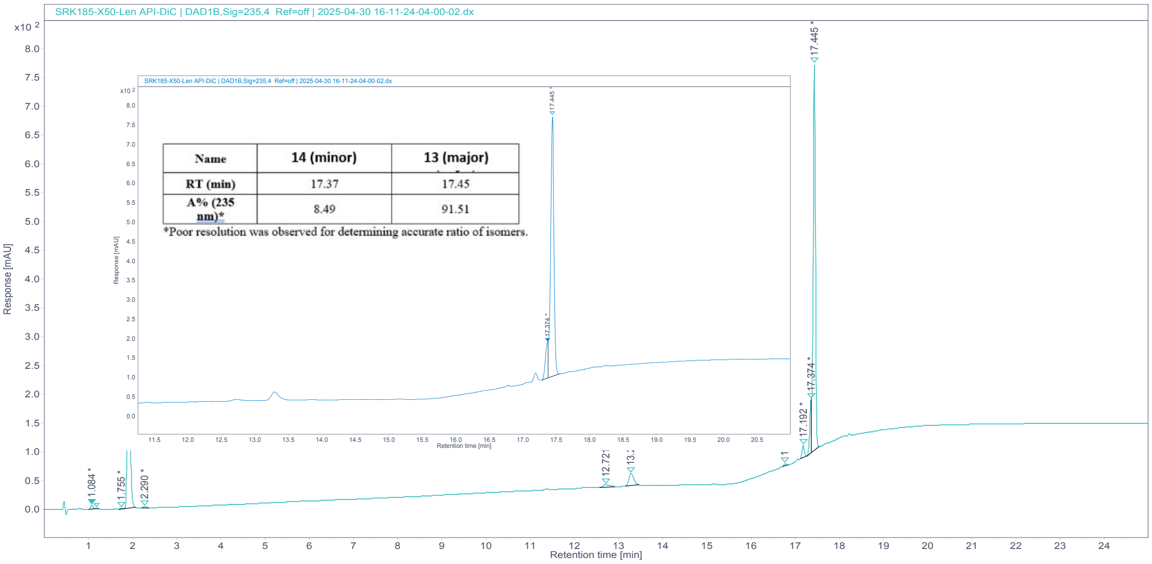

Figure S53. HPLC spectrum of **13** and atropoisomeric ratio based on A% (235 nm).

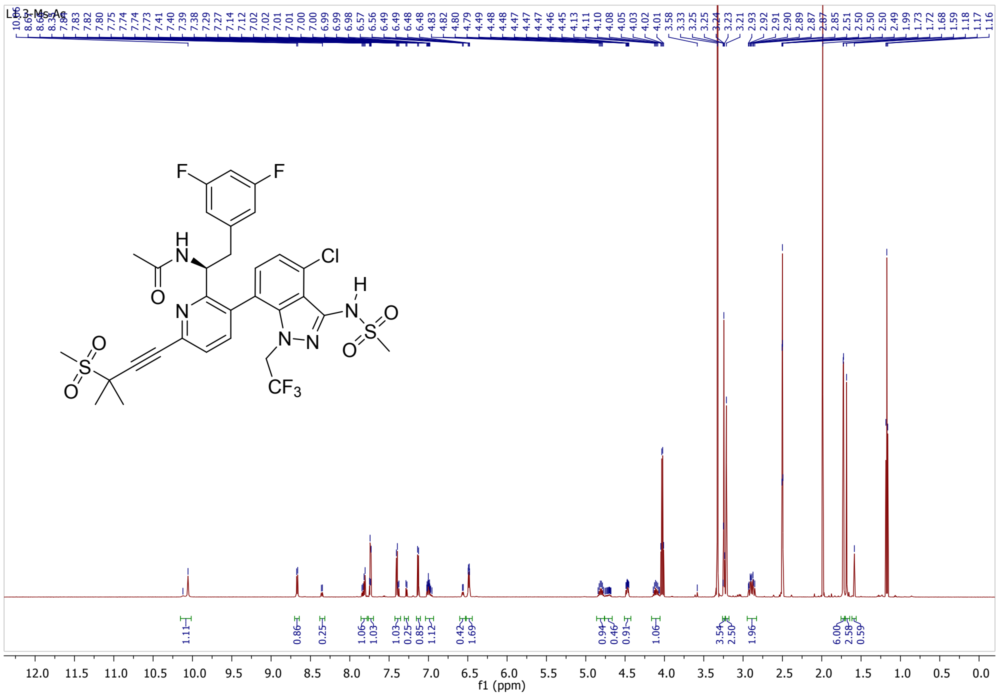

Figure S54. ^{1}H NMR (600 MHz, DMSO-d_6) of **14.**

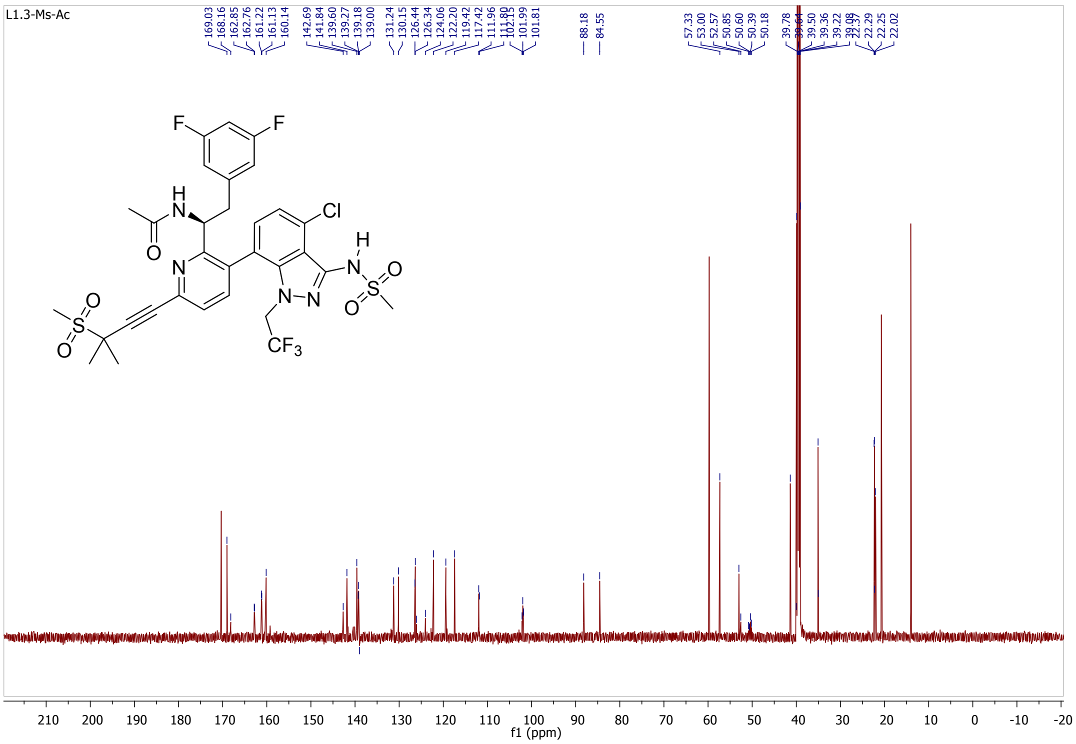

Figure S55. ^{13}C NMR (151 MHz, DMSO-d_6) of **14.**

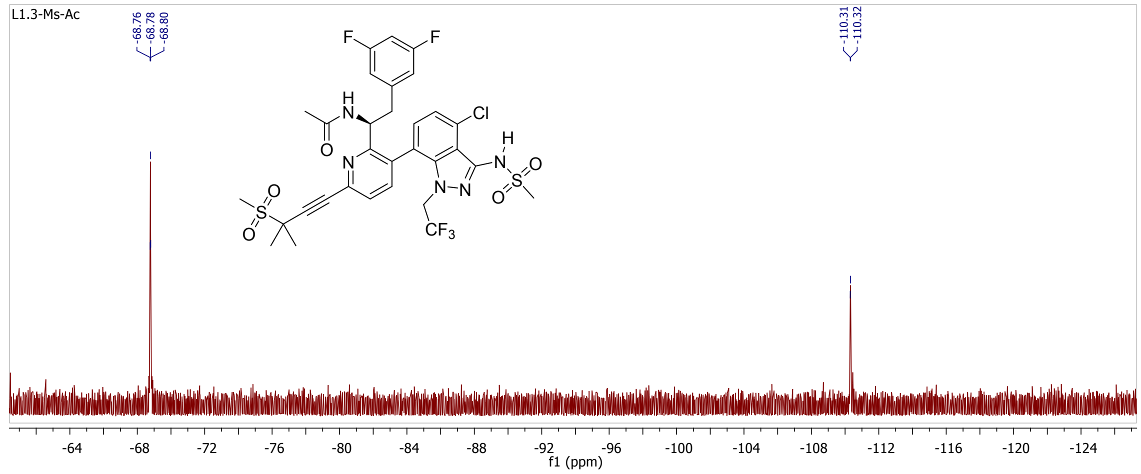

Figure S56. ^{19}F NMR (565 MHz, DMSO-d_6) of **14.**

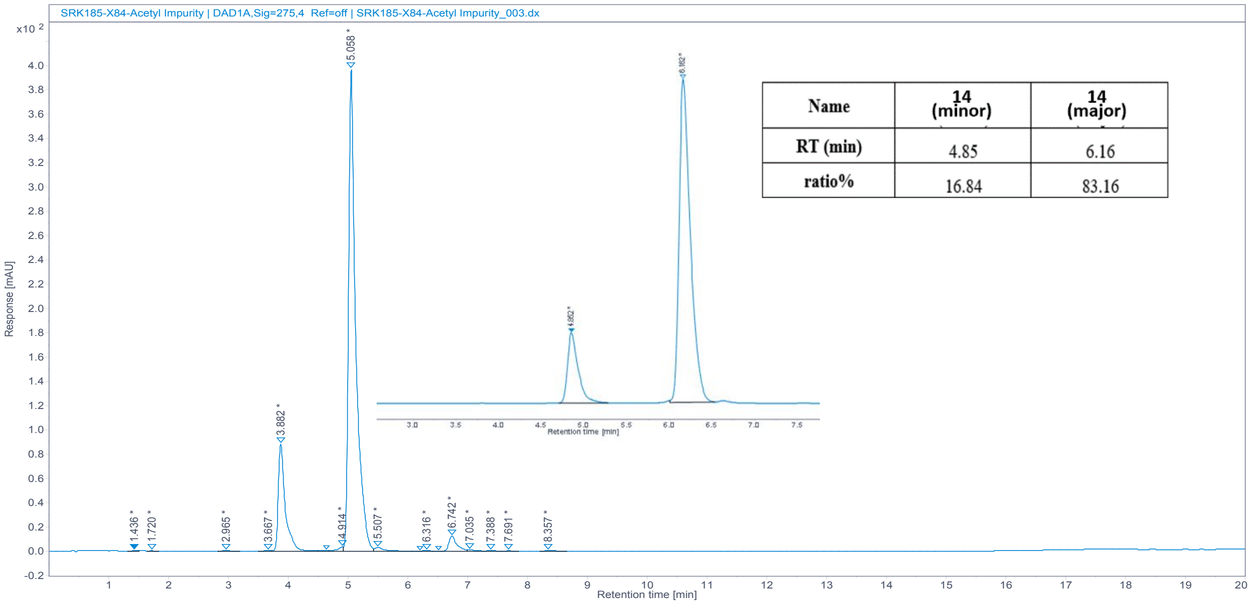

Figure S57. HPLC spectrum of **14** and atropoisomeric ratio based on A% (275 nm).

References

(1) PDR for Synthesis of Frag C of Lenacapavir, 2024. https://medicines4all.vcu.edu/media/medicines4all/assets/documents/Summary%20of%20Process%20Development%20Work%20on%20Asymmetric%20Synthesis%20of%20the%20Frag%20C%20of%20Lenacapavir,%20pp.%201-88.pdf.

(2) PDR for Synthesis of Frag B of Lenacapavir. https://doi.org/chrome-extehttps://medicines4all.vcu.edu/media/medicines4all/assets/documents/Len%252520Frag%252520B%252520PDR.pdf.

(3) PDR for Synthesis of Frag A of Lenacapavir. **2024**. https://medicines4all.vcu.edu/media/medicines4all/assets/documents/Len%20Frag%20A%20PDR.pdf.
(4) Allan, K. M.; Batten, A. L.; Brizgys, G.; Dhar, S.; Doxsee, I. J.; Goldberg, A.; Heumann, L. V.; Huang, Z.; Kadunce, N. T.; Kazerani, S.; Lew, W.; Ngo, V. X.; O'keefe, B. M.; Rainey, T. J.; Roberts, B. J.; Shi, B.; Steinhuebel, D. P.; Tse, W. C.; Wagner, A. M.; Wang, X.; Wolckenhauer, S. A.; Wong, C. Y.; Zhang, J. R. Methods and Intermediates for Preparing a Therapeutic Compound Useful in the Treatment of Retroviridae Viral Infection. WO2019161280A1, August 22, 2019.
(5) Bester, S. M.; Wei, G.; Zhao, H.; Adu-Ampratwum, D.; Iqbal, N.; Courouble, V. V.; Francis, A. C.; Annamalai, A. S.; Singh, P. K.; Shkriabai, N.; Van Blerkom, P.; Morrison, J.; Poeschla, E. M.; Engelman, A. N.; Melikyan, G. B.; Griffin, P. R.; Fuchs, J. R.; Asturias, F. J.; Kvaratskhelia, M. Structural and Mechanistic Bases for a Potent HIV-1 Capsid Inhibitor. *Science* **2020**, *370* (6514), 360–364. https://doi.org/10.1126/science.abb4808.
